# Calculation of the Surface Tension of Ordinary Organic and Ionic Liquids by Means of a Generally Applicable Computer Algorithm Based on the Group-Additivity Method

**DOI:** 10.3390/molecules23051224

**Published:** 2018-05-20

**Authors:** Rudolf Naef, William E. Acree

**Affiliations:** 1 Department of Chemistry, University of Basel, 4003 Basel, Switzerland; 2 Department of Chemistry, University of North Texas, Denton, TX 76203, USA; acree@unt.edu

**Keywords:** group-additivity method, surface tension

## Abstract

The calculation of the surface tension of ordinary organic and ionic liquids, based on a computer algorithm applying a refined group-additivity method, is presented. The refinement consists of the complete breakdown of the molecules into their constituting atoms, further distinguishing them by their immediate neighbour atoms and bond constitution. The evaluation of the atom-groups’ contributions was carried out by means of a fast Gauss-Seidel fitting method, founded upon the experimental data of 1893 compounds from literature. The result has been tested for plausibility using a 10-fold cross-validation (cv) procedure. The direct calculation and the cv test proved the applicability of the present method by the close similarity and excellent goodness of fit R^2^ and Q^2^ of 0.9039 and 0.8823, respectively. The respective standard deviations are ±1.99 and ±2.16 dyn/cm. Some correlation peculiarities have been observed in a series of ordinary and ionic liquids with homologous alkyl chains, as well as with di- and trihydroxy-groups-containing liquids, which have been discussed in detail, exhibiting the limit of the present method.

## 1. Introduction

Surface tension has received increasing interest in recent years due to its significance in material and environmental science, as well as in chemical separation processes, where it plays a key role in the dispersion and emulsion of immiscible solvent compositions, and adsorption at solid surfaces. A detailed discussion of the forces acting on the “interfacial region” (as it was named in order to include contributions of the second or third layer below the actual surface layer) was given by Fowkes [1]. He explained the surface tension as a result of the attractive forces from the underlying molecules net perpendicular to the surface, which causes a reduction in the number of molecules on the surface, which again increases their intermolecular distance. This increase requires work, which is the intrinsic reason for the tension, and is expressed as surface free energy upon its relaxation. The intermolecular attractive forces are separable into the London dispersive, polar, and Lewis acid-base forces, of which the former two are additive and the latter is non-additive, as has been outlined by van Oss et al. [2]. Based on these findings, Freitas et al. [3] developed a linear free energy relationship (LSER) equation, which—besides a constant—encompasses parameters representing the dipolarity, the excess molar refraction, the hydrogen bond acidity and basicity, and the molar volume of an organic liquid. They concluded from the weights of these parameters in the LSER equation that the dominant factors contributing to the surface tension are the dipolarity, the excess molar refraction, and the constant. The latter was interpreted as representing the loss of the dispersive interaction which any molecule suffers upon transfer to the surface. A further term, N_c_, was introduced, restricted to n-alkanes, in order to take account of the anisotropic properties found with linear alkanes larger than n-hexane, which was termed by Fowkes [4] as correlated molecular orientation (CMO), leading to enhanced adhesion on the liquid surface. The final result of the LSER equation for 299 compounds yielded a correlation coefficient of 0.936 and a standard error of 2.16 dyn/cm. A recent publication [5] has shown, with a homologous series of n-alkyl-substituted ionic liquids, that this CMO behaviour is not limited to the n-alkanes alone; in fact, the present paper will reveal further examples of normal compounds exhibiting anisotropism on the surface.

A number of further concepts have been developed, targeting a simple method for the reliable prediction of the surface tension and its temperature dependence. They essentially have in common that they do not claim to provide an explanation as to what kinds of intermolecular interactions are effective, and what influences the molecular orientations may exhibit at the surface of the liquids. In 1923, MacLeod [6], and later Sugden [7], observed that the fourth root of the experimental values of the surface tension correlated well with the product of the density difference between the liquid and the vapour state and a term called parachor, which has a nearly constant ratio to the critical volume. The fact that the parachor of a compound can be approximated as the sum of its constituting atomic and structural parachors enabled a direct estimation of the surface tension from the molecular structure. Sastri and Rao [8] found a correlation of the surface tension at the compound’s boiling point with the critical pressure, critical temperature, and the reduced boiling point, and extended this value to other temperatures. While in the previous examples the surface tension was evaluated from other known physical descriptors, a molecular mechanics force-field approach simulating the liquid state was applied, based on the molecule’s partial charges [9], yielding—besides numerous further properties—for 155 compounds (in the case of the surface tension) a root mean square deviation of 7.3 dyn/cm and a correlation coefficient of 0.89. An interesting concept applied a combination of an artificial neural network (ANN) with a group contribution method, in that 151 pre-defined chemical functional groups have been used as input and experimental surface-tension data as target on the output side in a three-layer neural network [10]. After training the ANN with 752 compounds at various temperatures and pressures, the result for the test set was surprisingly good, as it yielded an absolute average deviation of only 1.7% and a correlation coefficient of 0.995. In another approach, a multiple linear regression model was used to predict the surface tension of a total of 166 of alkanes, esters, and alcohols [11], founded upon a set of 10 descriptors with the highest correlation coefficients with the experimental surface tension, selected from of a total of 145 topological, geometrical, and electronic molecular descriptors. Some descriptors which exhibited a nonlinear relationship on a graphical plot were “linearized” by means of a suitable mathematical transform. An interesting point to be mentioned in view of the approach of the present paper is that 6 of the 10 descriptors are of topologic, 2 of electronic, and 2 of H-bonding nature. The final result for the 146 “training” compounds yielded a correlation coefficient of 0.983 and a standard deviation of 0.4 dyn/cm. An analogous concept to the one being presented here rested upon the well-known linear atom-group contribution method [12]. Due to the small number of only 12 special functional groups, some of them representing extended fragments, it was limited to a fairly narrow structural range of compounds.

The availability of a large number of experimental surface-tension data from many sources made it appealing to try to apply the highly versatile atom-group contribution method described in [13] for the calculation of this molecular descriptor, as this method has proven its outstanding success in the prediction of numerous thermodynamic [13,14], as well as solubility- [13,14,15], optics- [13], charge- [13] and environment-related [13], and physical [15] descriptors of an enormously diverse scope of molecular structures, without the requirement of any modification of the basic calculation algorithm. The goal was to provide a simple yet reliable means to predict the surface tension of a molecule, easily extendable to any kind of compound, e.g., also including the ionic liquids, of which this property is of particular importance in connection with their extraction and solvation capability for a large range of solutes.

## 2. General Procedure

The experimental surface-tension data are stored, together with the molecules in their 3D-geometry-optimized structure and further experimental and calculated descriptors, in an object-oriented knowledge database, at present encompassing more than 31,000 records of pharmaceuticals, plant protection products, dyes, ionic liquids, liquid crystals, metal-organics, lab intermediates, and more.

The details of the present atom-groups additivity method has been outlined in [13]. While the definitions and meanings of the atom groups in the following group-parameters table (Table 2) are to be interpreted in the same way as exemplified in Table 1 of [13], the inclusion of the ionic liquids required the addition of a number of further atom groups in order to represent their charged moieties, analogous to those given for the calculation of their viscosity in [15]. The exemplary list of these additional atom groups is collected in Table 1. These groups are treated just like the remaining ones by the computer algorithm.

For practical reasons—and following chemical conventions—the ion charges of the ionic liquids are centred on the atom types of the atom groups in Table 1 and Table 2. A certain deviation from this convention has been made for the imidazolium cations, where the conventional notation would imply an asymmetrcal charge distribution which, as e.g., the EHMO calculations indicate (visualized in Figure 1 in [15]), is not the case. Therefore, in this case, the positive charge has been positioned onto the carbon atom at position 2 between the two nitrogen atoms, which are bound to this central carbon atom by aromatic bonds. Accordingly, the carbon atom at position 2 and the nitrogen atoms in the imidazolium ions are represented in Table 1 by the atom groups 7 and 14, respectively. Atom types representing atoms that are immediate neighbours of charged atoms are distinguishable from those without charged neighbours by the added sign (in brackets) in their associated “Neigbours” definition (see examples 4, 6, 8–11, 14, 18, 19, 23, and 24 in Table 1).

The computer algorithm evaluating the atom-group parameters first collects from the database those molecules which fulfil the conditions for their inclusion into the parameters calculation, i.e., it checks the availability of an experimental surface-tension value and ensures that all atom groups in the molecule are present in the group-parameters table, and then carries out the parameters calculation using a fast Gauss-Seidel matrix-diagonalization procedure. Details of this entire algorithm have been outlined in [13]. Once the group parameters have been generated and stored in the parameters table, an immediate test of its predictive quality is carried out, first including all the compounds in the parameters evaluation, followed by a 10-fold cross-validation plausibility test, ensuring that each of the compounds has been introduced alternatively as both a test or training sample, as has been described in detail in Section 2.4 of [13]. These cross-validation calculations—and all the subsequent predictive descriptor calculations—are carried out using Equation (1), where *ST* is the surface-tension value, *a_i_* and *b_j_* are the contributions, *A_i_* is the number of occurrences of the *i*th atom group, and *B_j_* is the number of occurrences of the *j*th special group, and *C* is a constant. Yet, there is one further restriction beyond the ones mentioned above, in that for the predictive calculations of the surface tension of the training and test compounds, only those atom groups in the parameters table are considered valid which have been represented in the preceding parametrization process by at least three independent compounds with a known experimental surface-tension value.

(1) $$ST=\sum_{i}a_{i}A_{i}+\sum_{j}b_{j}B_{j}+C$$

The results of the parameters evaluations and cross-validation calculations are summed up at the bottom of Table 2 (rows A to H). The rightmost column lists the number of compounds representing the respective atom group. For several atom groups, this number falls short of the required number to render the group valid. Although these atom groups are not applicable for surface-tension predictions, they have been left in the parameters table for potential future use in this continuous project (and may motivate scientists working in this area to focus on compounds carrying the corresponding atom groups). The calculations are generally restricted to molecules containing the elements H, B, C, N, O, P, S, Si, and/or halogen.

The simple example of anisole (methyl phenyl ether) may help in understanding the use of equation 1 and Table 2: Anisole contains the following atom groups (n × “atom type/neighbours”: Contribution): 1 × “C sp3/H3O”: 3.14; 1 × “O/C2(pi)”: −1.00; 1 × “C aromatic/:C2O”: 3.7; 5 × “C aromatic/H:C2”: 1.01. The sum of the contributions of these atom groups is added to that of the constant “Const” (24.34): 3.14 − 1.00 + 3.7 + (5 × 1.01) + 24.34 = 35.23 dyn/cm. The experimental value was published in [16] as 35.7 dyn/cm.

## 3. Results

Since the value of the surface tension is highly sensitive to the experimental temperature conditions, and since several authors applied different temperatures as their own standard, an overall temperature standard was required in order to ensure comparability. The decision to choose 293.15 K as standard resulted from the observation that the majority of the authors referred to this temperature, and that measurements of another molecular physical property, the liquid viscosity (see [15]), also rested upon this standard. Where possible, e.g., if experiments at a series of temperatures have been published, the experimental surface-tension value was either linearly inter- or extrapolated if necessary, provided that the experimental temperature conditions did not deviate too much from this standard. The most productive source for experimental surface-tension data for ordinary liquid compounds, Jasper’s comprehensive paper [16], collecting some 2200 data from the year 1874 until 1969, stated that besides the temperature, other aspects, such as the method of measurement, the purity of the compounds, and even the experience of the investigator, had a major influence on the accuracy of the data. Unfortunately, he did not elaborate on the extent of the data uncertainty resulting from these aspects. This collection has been complemented—and its data compared—by the more recent collective papers [11,12,17,18]. Additionally, surface-tension data have been provided for various alkanes [19,20,21,22,23,24], alkylbenzenes [25], haloalkanes [26,27], halogenated esters and ethers [28], sulfoxides [29], and siloxanes [30,31]. Of particular interest are surface-tension data for ionic liquids. A recent comprehensive collection of publications in the supplement of [32], accumulated for the development of a further method for the prediction of the surface tension, based on the density, molar mass, and anion type, provided the source of data for 222 ionic liquids which have been included in the present studies.

In Table 2, the result of the atom-group parameters calculations, based on 1895 molecules, has been collected, together with a summary of the statistics data at the bottom (rows A to H). Attempts to further improve the result, e.g., by the exclusion of one or both of the special groups “Alkane” and “Unsaturated HC” (olefins and aromatics), yielded slightly lower correlation coefficients and higher standard deviations.

According to the entries A to H in Table 2, 165 (of 221) atom and special groups are valid for predictive calculations, as they are based on at least three independent training molecules. Therefore, the result of the goodness of fit R^2^ of 0.9039 was based on 1833 of the 1893 training compounds, with a standard deviation σ of 1.99 dyn/cm. The average statistics data of the ten 10-fold cross-validation calculations (entries F–H) rested on a total of 1769 compounds, resulting in a cross-validated goodness of fit Q^2^ of 0.8823 and a standard deviation S of 2.16 dyn/cm. The standard deviations σ and S (entries D and H) have been calculated from the training set and the combined test sets of the cross-validation calculations, respectively, using the well-known Equation (2), where *SD* is the respective standard deviation, *x* the experimental, $\bar{x}$ the calculated surface tension of each molecule, and *N* the number of molecules. (The corresponding average deviations — entries C and G — are the sum of the absolute differences between the experimental and calculated surface tension of all involved compounds, divided by the number of these compounds. Since the standard deviation is more widely used in the examination of the reliability of predictive calculations, corresponding discussions in this paper refer to this value.)

(2) $$SD=\sqrt[{}]{\frac{\sum_{N}{\left(x-\bar{x}\right)}^{2}}{N-1}}$$

**Figure 1:**
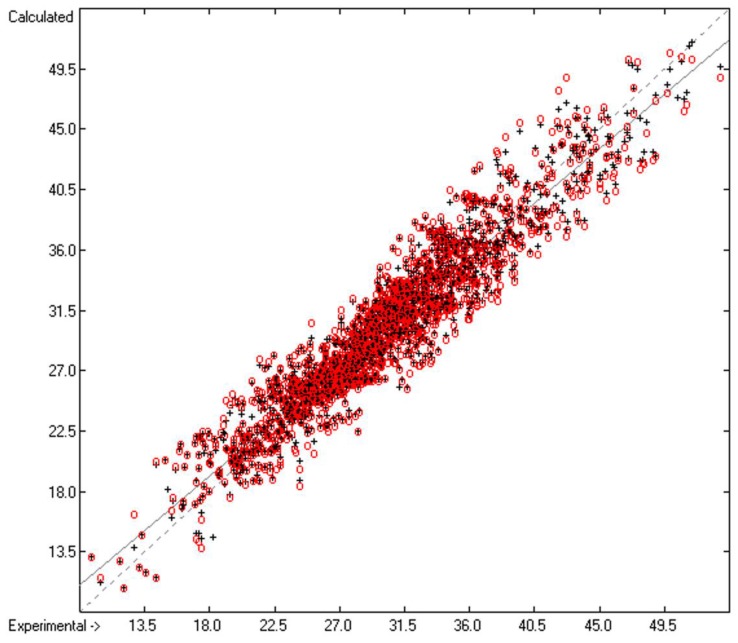
Correlation diagram of the surface-tension data (in dyn/cm). Cross-validation data are added as red circles. (*N* = 1833; R^2^ = 0.9039; Q^2^ = 0.8823; regression line: intercept = 2.8653; slope = 0.9049).

**Figure 2:**
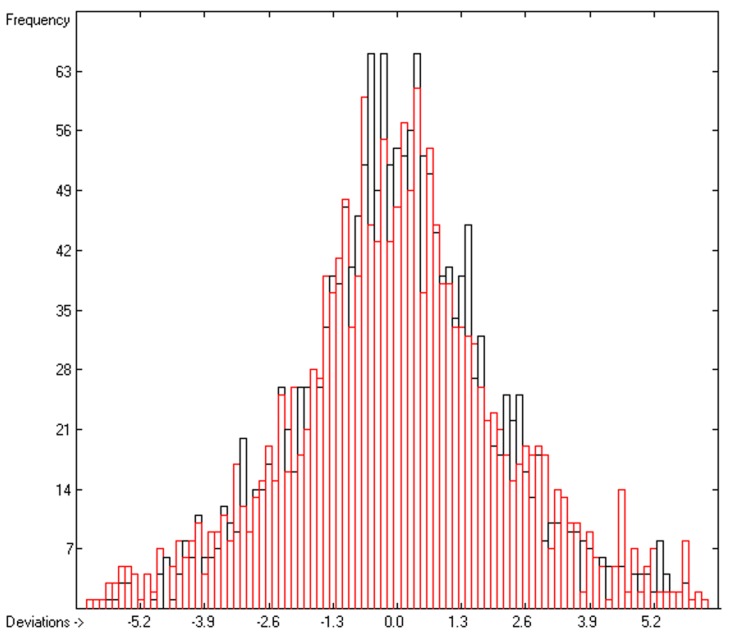
Histogram of the surface-tension data. Deviations are in dyn/cm. Cross-validation data are superpositioned as red bars. (S = 2.16; experimental values range: 9.89—53.5 dyn/cm).

The excellent compliance, in most cases, between the black crosses of the training set with the affiliated red circles of the cross-validated set in Figure 1, as well as the close similarity of standard deviations R^2^ and Q^2^, confirm the applicability of the present surface-tension prediction method. The corresponding histogram in Figure 2 exhibits a fairly even Gaussian distribution for both the direct and the cross-validated deviations. A list of all the compounds used in this study, their experimental and calculated data and their 3D structures is available online in the supplementary material.

**Figure 3:**
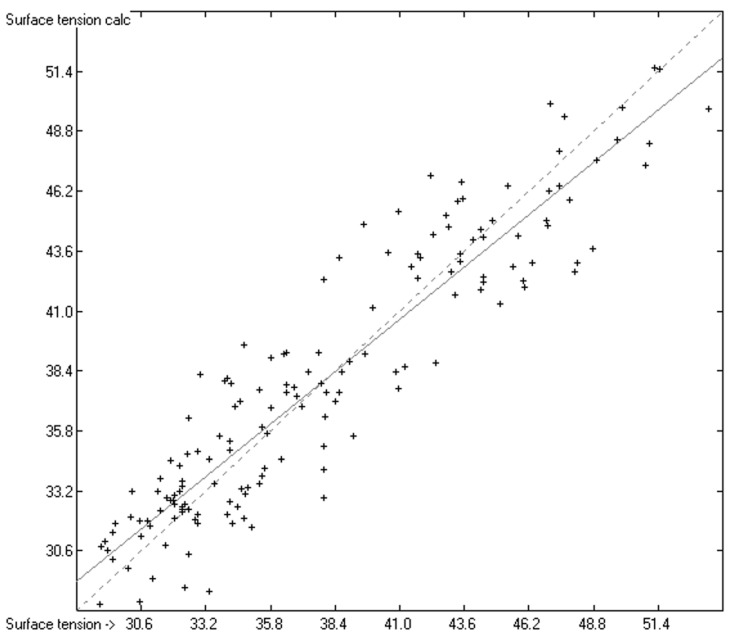
Correlation diagram of the surface-tension data of the ionic liquids (in dyn/cm). (*N* = 154; R^2^ = 0.8579).

The relatively large standard deviation in relation to the overall data range, however, obscures the otherwise bright picture of the good correlation between the experimental and predicted surface-tension values, in that it hides three important observations. The first observation concerns the reliability of the experimental data for the ionic liquids, a point that has already been referred to in [32]. A typical example is 1,3-dimethylimidazolium bis(trifluoromethylsulfonyl)amide, for which in [5] a surface tension of 39 dyn/cm was given at 298.3 K, whereas in [33] a value 36.3 dyn/cm at the same temperature was published. In a further example, the surface tensions of each of the complete series of 1-alkyl-3-methylimidazolium bis(trifluoromethylsulfonyl)amide (with alkyl being ethyl to decyl) varied by ca. 1.5 to 2.1 dyn/cm at 298.15 K between the two publications [33] and [34]. Due to their hygroscopicity and high viscosity, a higher uncertainty, and thus scatter, of the experimental values should be expected, as is reflected in Figure 3. As a further consequence, the number of ionic liquid outliers, i.e., compounds for which the values exceed three times the cross-validated standard deviation, are disproportionately higher (26.6%) than the 4.8% for the normal compounds (see the outliers list in the supplementary material). The small number of ionic liquids compared with the complete set of compounds, however, did not impede them from remaining included in the parameters calculations without undue deterioration of the result.

**Figure 4:**
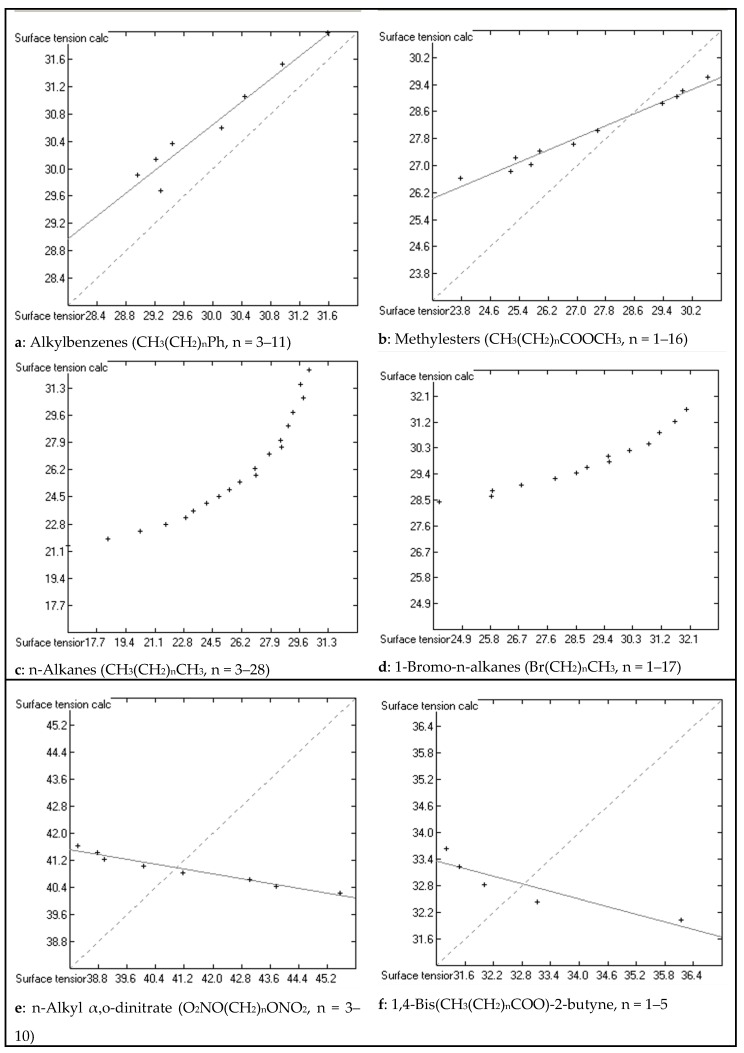
Correlation diagrams of the surface tension (in dyn/cm) of ordinary organic liquids with linear n-alkyl chains.

**Figure 5:**
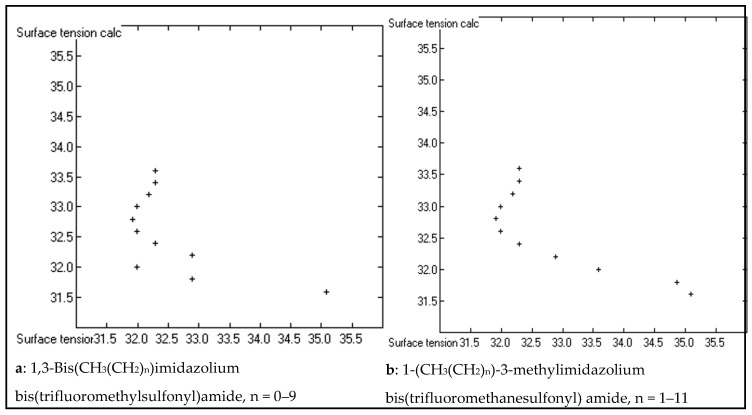
Correlation diagrams of the surface tension (in dyn/cm) of ionic liquids with linear n-alkyl chains.

The second observation, disguised behind the range of the standard deviation, reveals an important shortcoming of the present prediction method. A small set of compound classes, characterized by the common feature of carrying a homologous sequence of linear methylene chains, exhibits an unexpected deviation of the experimental sequence of surface-tension data from the calculated values, whereas other analogous classes of homologues show fairly normal correlation between experiment and prediction. Typical examples of the latter normal correlation sequence are *n*-alkylbenzenes [3] (chart a in Figure 4), methylesters of long-chain carboxylic acids [35] (chart 4b), 1-alkanols [36], and 1-alkylthiols [37], which only deviate from the ideal correlation by slightly differing slopes. In contrast to this, the sequence of the experimental surface-tensions in the homologous n-alkane series [38] (chart 4c) is nonlinear and seems to aim at a constant maximum with increasing chain length. Analogous nonlinearity with increasing chain length was found for 1-alkenes [38] and 1-bromoalkanes [39] (chart 4d). A nearly linear but inverse correlation was found for a methylene chain homologue substituted at both ends by a nitrate group [40] (chart 4e). This characteristic feature was also found for the homologues of α,o-dibromo-*n*-alkane [41] and 1,4-Bis(*n*-alkylcarbonyloxy)-2-butyne [42] (chart 4f). Quite a bizarre surface-tension sequence was revealed by the symmetrical (chart a in Figure 5) and asymmetrical (chart 5b) homologues of the ionic liquids 1,3-Bis(*n*-alkyl)imidazolium and 1-*n*-alkyl-3-methylimidazolium bis(trifluoromethylsulfonyl)amide [5,33,34], respectively. It is obvious that the present atom-group additivity approach is not able to treat these highly heterogeneous sequences. The reason behind these deviations has been described by Fowkes [4] as a result of anisotropism on the liquid surface caused by the extensive molecular directional orientation of these compounds, leading to a correlated molecular orientation (CMO). However, Fowkes only related his CMO thesis to linear n-alkanes; its extension to compounds with various substitutions inside or at the end of the methylene chains remains open to further studies. Since the CMO effect is generally small in relation to the other attractive forces on the liquid surface—Fowkes evaluated a range of between 0 for hexane and 2.89 dyn/cm for hexadecane, i.e., ca. 10% of the total force for the largest n-alkane in the series—the maximum range of the surface tension of all these homologous series remained within the deviation limits to allow all of their members to stay included in the parameters calculation. As a consequence, however, the present atom-group additivity method at best provides an average value for the surface tension of these homologues.

The third observation, another form of special intermolecular association, was apparent on comparing the experimental surface tension of di- and tri-hydroxy-group-containing compounds with their calculated value, as these systematically by far underestimated the measured values. (An analogous observation was made for hydrazine, ethanolamine, propanolamine, 2-(isopropylamino)ethanol, and ethylenediamine.) Evidently, the excessive increase of the experimental surface tension is caused by an effect that is not captured by the ordinary hydroxy-group parameter (entry 157 in Table 2) and is most probably best described as additional associative intermolecular H-O bond forces. Therefore, a special group (entry 219 in Table 2) has been introduced to take account of the surplus effect of each additional hydroxy group, which indeed improved conformance with the experimental values. Nevertheless, due to the large scatter of the experimental values, which did not indicate any systematic correlation with the corresponding molecular structure—compare, e.g., the experimental surface tensions of the two closely related outliers 1,2,3-propanetriol and 1,2,6-hexanetriol showing values of 63.3 and 44.14 dyn/cm [12], respectively, and on the other hand those of the two structurally very different compounds ethylene glycol and heptaethylene glycol exhibiting experimental values of 48.43 and 48.39 dyn/cm [16], respectively—11 of the 21 examples with available data still exceeded the deviation limits and had to remain in the outliers list.

Barring these special cases, the overwhelming majority of surface tension data have shown a normal statistical pattern in relation to the predictions, clearly proving the applicability of the present group-additivity approach. But how well does it compare with other published methods? Since to the best of our knowledge the present calculations are founded on the largest set of compounds with experimental surface-tension data, a direct comparison of their reliability with earlier papers, often focusing on only a limited number of closely related compounds, seems of little use. For instance, the most similar concept to the present group-additivity method, published in 2000 [12], yet only applying 12 functional groups, yielded a correlation coefficient R^2^ of 0.754, based on a training set of only 349 compounds of structurally limited extent. The correlation coefficient of 0.995 and average deviation of 1.7% of the ANN method [10] mentioned earlier, on the other hand, are surprisingly good—and questionable—insofar as for a number of compound examples the experimental values, which have been measured by various scientific groups, scatter by far more than 1.7%, as has been demonstrated in the comprehensive paper [16]. Beyond this, any prediction of a property by means of the ANN method is inevitably bound to the computer incorporating the trained artificial network. By contrast, the greatest advantage of the present approach lies in the fact that no computer is required: The prediction of the surface tension of a compound takes only a simple 2D drawing on a sheet of paper to help to find all the atom groups—and the parameters of Table 2 to sum up their contributions as exemplified at the bottom of Section 2. The large number of presently 165 valid atom groups in Table 2 enables the surface-tension prediction of a wide range of structurally varying molecules, which is evidenced by the surface-tension calculability of 55% of the currently 31,212 compounds in ChemBrain’s database, which can be viewed as representative for the entire structural coverage of chemicals.

## 4. Conclusions

The present results prove the reliable applicability of the atom-group additivity approach on the molecular surface-tension prediction by simply extending, by a few further lines of control code, the common computer algorithm outlined in [13], which has already demonstrated its extraordinary versatility with the trustworthy prediction of 13 further descriptors described in the previous papers [13,14,15] in a split second in one single sweep on a desktop computer: The heats of combustion, formation (indirectly), solvation, sublimation and vaporization, the entropy of fusion, the partition coefficient logP_o/w_, the solubility logS_water_, the refractivity, the polarizability, the toxicity against the protozoan *Tetrahymena pyriformis*, the liquid viscosity, and the activity coefficient at infinite dilution. In addition, the present method has the advantage of enabling an easily generalizable computer algorithm for the definition of the atom groups, i.e., the atom types and their neighbours. The present work is part of an ongoing project called ChemBrain IXL available from Neuronix Software (www.neuronix.ch, Rudolf Naef, Lupsingen, Switzerland).

## Figures and Tables

**Table 1:** Atom-group examples for ionic liquids and their meaning.

No	Atom Type	Neighbours	Meaning	Example
1	B(−)	C4		B^−^ in tetracyanoborate
2	B(−)	CF3	C**B**F_3_^−^	alkyltrifluoroborate
3	B(−)	F4	**B**F_4_^−^	tetrafluoroborate
4	C sp3	H3B(−)	H_3_CB^−^	C in methyltrifluoroborate
5	C(−) sp3	C3		central C^−^ in tricyanocarbeniate
6	C aromatic	H:C:N(+)	C:**C**H:N^+^	C2 in pyridinium
7	C(+) aromatic	C:N2	N:**C**(C):N	C2 in 2-alkylimidazolium
8	C sp	C#N(−)	C^−^(**C**#N)	cyano-C in tricyanocarbeniate
9	C sp	N#N(−)	N^−^(**C**#N)	C in dicyanoamide
10	C sp	B#N(−)	B^−^(**C**#N)	C in tetracyanoborate
11	C sp	=N=S(−)	N=**C**=S^−^	thiocyanate
12	N(+) sp3	C4	**N**^+^C_4_	tetraalkylammonium
13	N(+) sp2	O2=O(−)	**N**O_3_^−^	nitrate
14	N aromatic	C2:C(+)	C-**N**(C):C^+^	N1 in 1-alkylimidazolium
15	N(+) aromatic	C:C2	C:**N**^+^(C):C	N in 1-alkylpyridinium
16	N(−)	C2	C-**N**^−^-C	N^−^ in dicyanoamide
17	N(−)	S2	S-**N**^−^-S	bis(trifluoromethanesulfonyl)amide
18	P4	CO2=O(−)	C**P**O3^−^	alkylphosphonate
19	P4	O3=O(−)	O=**P**O_3_^−^	dialkylphosphate
20	P(+)	C4	**P**C_4_^+^	tetraalkylphosphonium
21	P(−)	C3F3	F_3_**P**^−^C_3_	tris(pentafluoroethyl)trifluorophosphate
22	P(−)	F6	**P**F_6_^−^	hexafluorophosphate
23	S4	CO=O2(−)	C**S**O_3_^−^	alkylsulfonate
24	S4	O2=O2(−)	**S**O_4_^−^	alkylsulfate

**Table 2:** Atom groups and their contributions for surface-tension calculations.

Entry	Atom Type	Neighbours	Contribution	Occurrences	Molecules
1	Const		24.34	1893	1893
2	B	C3	−4.5	1	1
3	B	O3	0.6	6	6
4	B(−)	C4	17.49	5	5
5	B(−)	CF3	1.11	5	5
6	B(−)	F4	1.67	10	10
7	C sp3	H3B(−)	1.21	1	1
8	C sp3	H3C	−2.28	3048	1529
9	C sp3	H3C(+)	29.45	3	3
10	C sp3	H3N	7.1	111	104
11	C sp3	H3N(+)	8.27	34	23
12	C sp3	H3O	3.14	194	155
13	C sp3	H3S	4.71	13	11
14	C sp3	H3S(+)	4.66	1	1
15	C sp3	H3P	5.46	2	2
16	C sp3	H3Si	−0.65	113	18
17	C sp3	H2BC	2.28	3	1
18	C sp3	H2BC(−)	−6.78	3	3
19	C sp3	H2C2	0.2	6359	1366
20	C sp3	H2C2(+)	2.04	7	7
21	C sp3	H2CN	6.38	271	175
22	C sp3	H2CN(+)	6.13	74	46
23	C sp3	H2CO	2.92	1277	673
24	C sp3	H2CP	7.04	16	15
25	C sp3	H2CP(+)	2.87	24	6
26	C sp3	H2CS	4.57	76	55
27	C sp3	H2CS(+)	6.64	5	2
28	C sp3	H2CSi	3.91	12	5
29	C sp3	H2CF	−1.65	5	5
30	C sp3	H2CCl	4.93	51	39
31	C sp3	H2CBr	6.41	41	33
32	C sp3	H2CJ	9.03	28	21
33	C sp3	H2O2	6.18	7	7
34	C sp3	HC3	1.56	428	304
35	C sp3	HC2N	7.61	15	12
36	C sp3	HC2N(+)	8.4	6	6
37	C sp3	HC2O	5.08	150	116
38	C sp3	HC2S	5.65	8	5
39	C sp3	HC2Cl	4.68	12	12
40	C sp3	HC2Br	6.37	10	10
41	C sp3	HC2J	9.46	4	4
42	C sp3	HCO2	7.1	15	13
43	C sp3	HCF2	−1.62	29	17
44	C sp3	HCCl2	5.35	12	11
45	C sp3	HCBr2	12.05	3	2
46	C sp3	HO3	9.87	3	3
47	C sp3	C4	3.1	111	104
48	C sp3	C3N	4.53	2	2
49	C sp3	C3O	6.42	36	36
50	C sp3	C3S	4.75	3	3
51	C sp3	C3F	2.23	21	11
52	C sp3	C3Cl	7.32	42	33
53	C sp3	C3Br	3.73	1	1
54	C sp3	C2F2	−0.4	333	57
55	C sp3	C2Cl2	−0.14	2	2
56	C sp3	CNF2	8.26	9	3
57	C sp3	COF2	2.99	34	18
58	C sp3	CF3	−4.98	111	49
59	C sp3	CSF2	0.05	2	1
60	C sp3	CF2Cl	−0.7	2	1
61	C sp3	CPF2(−)	2.75	33	11
62	C sp3	CFCl2	−0.74	1	1
63	C sp3	CCl3	4.6	10	9
64	C sp3	N3F(+)	−4.3	1	1
65	C sp3	SF3	−2.31	110	57
66	C(−) sp3	C3	9.33	3	3
67	C sp2	H2=C	−2.43	78	77
68	C sp2	HB=C(−)	2.74	1	1
69	C sp2	HC=C	1	266	174
70	C sp2	HC=O	2.74	13	13
71	C sp2	H=CN	2.75	216	108
72	C sp2	H=CO	0.25	9	9
73	C sp2	H=CS	4.09	32	30
74	C sp2	H=CCl	0.8	5	3
75	C sp2	H=CBr	−1.85	1	1
76	C sp2	HN=O	10.39	2	2
77	C sp2	HO=O	1.28	13	13
78	C sp2	C2=C	3.15	67	56
79	C sp2	C2=N	5.54	35	29
80	C sp2	C2=O	6.2	73	72
81	C sp2	C=CO	1.71	3	3
82	C sp2	C=CS	5.29	25	24
83	C sp2	C=CCl	3.33	9	5
84	C sp2	C=CBr	7.47	3	3
85	C sp2	CN=O	7.25	2	2
86	C sp2	CO=O	2.25	737	528
87	C sp2	CO=O(−)	−2.99	23	23
88	C sp2	=COS	7.31	2	2
89	C sp2	C=OCl	7.53	1	1
90	C sp2	C=OBr	11.42	1	1
91	C sp2	=CSCl	7.43	3	2
92	C sp2	=CSBr	10.37	3	2
93	C sp2	=CSJ	15.69	1	1
94	C sp2	=CCl2	2.74	6	4
95	C sp2	NO=O	6.06	7	4
96	C sp2	O2=O	2.64	12	12
97	C sp2	OS=S	9.18	5	5
98	C aromatic	H:C2	1.01	1614	344
99	C aromatic	H:C:N	4.01	106	63
100	C aromatic	H:C:N(+)	8.32	33	18
101	C aromatic	H:N2	2.23	1	1
102	C aromatic	:C3	1.65	119	60
103	C aromatic	C:C2	2.25	313	254
104	C aromatic	C:C:N	5.49	21	20
105	C aromatic	C:C:N(+)	13.44	3	3
106	C aromatic	:C2N	6.23	19	19
107	C aromatic	:C2N(+)	10.03	10	10
108	C aromatic	:C2:N	9.45	1	1
109	C aromatic	:C2O	3.7	32	29
110	C aromatic	:C2S	6.74	9	9
111	C aromatic	:C2Si	3.98	4	3
112	C aromatic	:C2F	−0.4	9	8
113	C aromatic	:C2Cl	3.95	21	17
114	C aromatic	:C2Br	7.09	4	4
115	C aromatic	:C2J	9.69	3	3
116	C(+) aromatic	H:N2	0.96	104	104
117	C(+) aromatic	C:N2	−22.73	10	10
118	C sp	H#C	1.5	24	24
119	C sp	B#N(−)	−4.57	20	5
120	C sp	C#C	1.74	56	40
121	C sp	C#N	5.9	63	62
122	C sp	C#N(−)	−2.15	9	3
123	C sp	N#N(−)	0.69	16	8
124	C sp	#NS	9.65	4	4
125	C sp	=N=S	4.87	4	4
126	C sp	=N=S(−)	4.68	7	7
127	N sp3	H2C	−3.52	28	28
128	N sp3	H2C(pi)	4.36	6	6
129	N sp3	H2N	0.56	5	5
130	N sp3	HC2	−9.48	20	20
131	N sp3	HC2(pi)	−4.7	7	7
132	N sp3	HC2(2pi)	5.79	1	1
133	N sp3	HCN(pi)	9.39	2	2
134	N sp3	HSi2	−2.29	1	1
135	N sp3	C3	−14.79	18	18
136	N sp3	C3(pi)	−11.8	7	7
137	N sp3	C2N	−7.92	4	4
138	N sp3	C2N(pi)	0.56	6	6
139	N sp3	C2N(2pi)	4.42	2	2
140	N(+) sp3	HC3	−5.95	1	1
141	N(+) sp3	C4	−5.84	21	21
142	N aromatic	HC:C(+)	9.3	3	3
143	N aromatic	:C2	−2.38	64	63
144	N aromatic	C2:C(+)	0.45	225	114
145	N aromatic	:C:N	7.05	2	1
146	N(+) aromatic	C:C2	−2.46	18	18
147	N sp2	C=C	0	4	4
148	N sp2	=CN	−0.22	12	6
149	N sp2	C=N	−1.94	6	3
150	N sp2	=CO	0.08	23	23
151	N sp2	N=O	−2.47	9	9
152	N sp2	O=O	3.03	3	3
153	N(+) sp2	CO=O(−)	1.7	22	20
154	N(+) sp2	O2=O(−)	5.7	23	13
155	N(−)	C2	−0.36	8	8
156	N(−)	S2	−6.68	54	54
157	O	HC	0.58	161	150
158	O	HC(pi)	1.07	90	90
159	O	HN(pi)	3.2	6	6
160	O	HO	25.77	2	1
161	O	HP	0.31	13	7
162	O	HS	9	3	3
163	O	BC	−1.36	18	6
164	O	C2	−4.02	288	181
165	O	C2(pi)	−1	725	561
166	O	C2(2pi)	4.81	5	5
167	O	CN(pi)	−2.67	16	16
168	O	CN(+)(pi)	−0.78	22	12
169	O	CN(2pi)	5.88	4	4
170	O	CP	−0.41	168	75
171	O	CP(pi)	−3.25	3	1
172	O	CS	1.66	35	23
173	O	CSi	−3.09	23	5
174	O	Si2	1.66	30	9
175	P3	O3	−1.63	20	20
176	P4	HO2=O	2.98	16	16
177	P4	C2O=O(−)	−9.24	1	1
178	P4	CO2=O	−2.1	15	15
179	P4	CO2=O(−)	0.34	1	1
180	P4	O3=O	3.17	13	13
181	P4	O3=O(−)	−6.46	6	6
182	P4	O2=OF	0.52	2	2
183	P4	O2=OCl	5.47	2	2
184	P4	O=OCl2	8.03	2	2
185	P(−)	C3F3	−4.02	11	11
186	P(−)	F6	−4.92	6	6
187	P(+)	C4	0.32	6	6
188	S2	HC	−1.34	11	11
189	S2	HC(pi)	2.89	1	1
190	S2	C2	−2.44	20	20
191	S2	C2(pi)	−3.02	15	15
192	S2	C2(2pi)	−3.49	33	33
193	S2	CS	0.42	20	12
194	S4	C2=O	5.9	3	3
195	S4	CN=O2(−)	0.07	108	54
196	S4	CO=O2	9.34	3	3
197	S4	CO=O2(−)	−5.01	5	5
198	S4	C=O2F	1.52	1	1
199	S4	C=O2Cl	7.24	5	5
200	S4	O2=O	−0.65	8	8
201	S4	O2=O2	3.42	4	4
202	S4	O2=O2(−)	−6.44	7	7
203	S4	O=O2S	−0.4	4	4
204	S(+)	C3	8.96	2	2
205	Si	HC3	−8.53	1	1
206	Si	HC2Cl	−5.31	1	1
207	Si	HCCl2	−4.27	1	1
208	Si	HO3	4.24	1	1
209	Si	C4	−7.91	4	4
210	Si	C3N	0	2	1
211	Si	C3O	−3.02	14	7
212	Si	C3Cl	−4.63	1	1
213	Si	C3Br	−2.81	3	3
214	Si	C2O2	−0.04	21	6
215	Si	C2Cl2	−2.94	1	1
216	Si	C2Br2	1.98	1	1
217	Si	CCl3	−3.39	1	1
218	Si	O4	7.84	6	4
219	(COH)n	COH groups: n > 1	3.26	11	10
220	Alkane	No of C atoms	0.22	1263	125
221	Unsaturated HC	No of C atoms	0.02	1314	125
A	Based on	Valid groups	165		1893
B	Goodness of fit	R^2^	0.9039		1833
C	Deviation	Average	1.53		1833
D	Deviation	Standard	1.99		1833
E	K-fold cv	K	10		1769
F	Goodness of fit	Q^2^	0.8823		1769
G	Deviation	Average (cv)	1.66		1769
H	Deviation	Standard (cv)	2.16		1769

## Supplementary Materials

Supplementary materials are available online at http://www.mdpi.com/1420-3049/23/5/1224/s1. The list of compounds, their experimental and calculated data and 3D structures of the surface-tension calculations are available online under the names of “S1. Experimental and Calculated Surface-Tension Data Table.doc” and “S2. Compounds List of Surface-Tension Calculations.sdf”. A list of their outliers has been added under the name of “S3. Compounds List of Surface-Tension Outliers.xls”. The figures are available as tif files and the tables as doc files under the names given in the text.
