# Supplementary material for: Calculation of the Surface Tension of Ordinary Organic and Ionic Liquids by Means of a Generally Applicable Computer Algorithm Based on the Group-Additivity Method
Source: Molecules. 2018 May 20;23(5):1224. doi: 10.3390/molecules23051224 (PMC6099982; doi:10.3390/molecules23051224)
Supplement: Supplementary file 1 [file molecules-23-01224-s001.zip › S1. Experimental and Calculated Surface-Tension Data Table.doc]

|  | **Molecules sorted by deviation** |  |  |  |  |
| --- | --- | --- | --- | --- | --- |
| **Entry** | **Molecule name** | **Surface tension exp** | **Surface tension calc** | **Surface tension test** | **Deviation** |
|  |  |  |  |  |  |
| 1 | Diisobutyl maleate | 28.79 | 28.68 | 28.79 | 0.00 |
| 2 | Methyl 9-decynoate | 33.10 | 33.37 | 33.10 | 0.00 |
| 3 | Methyl isoamyl ketone | 25.57 | 25.66 | 25.56 | 0.01 |
| 4 | 1-Ethyl-3-methylimidazolium butylsulfate | 39.64 | 39.16 | 39.65 | -0.01 |
| 5 | n-Butyl acetate | 24.35 | 24.35 | 24.34 | 0.01 |
| 6 | 1-Tridecyne | 27.56 | 27.56 | 27.57 | -0.01 |
| 7 | Di-n-propylsulfite | 28.69 | 28.69 | 28.68 | 0.01 |
| 8 | Ethyl amyl ether | 22.20 | 22.20 | 22.21 | -0.01 |
| 9 | Ethyl 2-acetylpentanoate | 29.86 | 29.83 | 29.87 | -0.01 |
| 10 | 2,6-Dimethylheptane | 21.00 | 20.92 | 21.01 | -0.01 |
| 11 | beta-Ethyl-beta-methylstyrene | 31.80 | 31.65 | 31.81 | -0.01 |
| 12 | Diethyl phthalate | 36.54 | 36.66 | 36.55 | -0.01 |
| 13 | Dimethyl chloromaleate | 37.54 | 37.45 | 37.52 | 0.02 |
| 14 | Dibutoxymethane | 24.57 | 24.56 | 24.55 | 0.02 |
| 15 | 3-Ethyl-2-methylpentane | 20.59 | 20.50 | 20.57 | 0.02 |
| 16 | Diethylene glycol dimethyl ether | 30.14 | 30.24 | 30.16 | -0.02 |
| 17 | 3-Methyl-1-pentanol | 25.30 | 25.24 | 25.32 | -0.02 |
| 18 | Ethyl 2-cyano-3-ethylvalerate | 30.96 | 31.09 | 30.98 | -0.02 |
| 19 | Diethylcarbonate | 26.44 | 26.26 | 26.46 | -0.02 |
| 20 | 2-Propoxy-4-methyl-1,3,2-dioxaphospholane | 28.06 | 28.04 | 28.08 | -0.02 |
| 21 | Dipentyl carbonate | 27.47 | 27.46 | 27.49 | -0.02 |
| 22 | Methyl diethylmalonate | 31.04 | 30.99 | 31.06 | -0.02 |
| 23 | Propyl 3-butynoate | 29.88 | 29.87 | 29.85 | 0.03 |
| 24 | 3-Pentanone O-ethyloxime | 23.78 | 23.77 | 23.75 | 0.03 |
| 25 | n-Butyl cinnamate | 35.75 | 35.93 | 35.78 | -0.03 |
| 26 | 3-Pentanone ketazine | 26.66 | 26.66 | 26.69 | -0.03 |
| 27 | Ethyl trichloroacetate | 30.82 | 30.83 | 30.85 | -0.03 |
| 28 | 2-Nitroanisol | 45.70 | 45.95 | 45.66 | 0.04 |
| 29 | Bis(2-hydroxyethyl)methylamine | 40.15 | 39.67 | 40.19 | -0.04 |
| 30 | Dipropyl pentylmalonate | 28.69 | 28.60 | 28.65 | 0.04 |
| 31 | Ethyl 2-allylacetoacetate | 30.30 | 30.28 | 30.26 | 0.04 |
| 32 | 3,3-Dimethylheptane | 21.13 | 21.10 | 21.09 | 0.04 |
| 33 | Dodecamethylpentasiloxane | 17.08 | 17.02 | 17.12 | -0.04 |
| 34 | Dibutyl phosphite | 28.24 | 28.58 | 28.28 | -0.04 |
| 35 | Hexadecamethylheptasiloxane | 17.61 | 17.66 | 17.65 | -0.04 |
| 36 | Ethyl N-methylcarbamate | 32.48 | 32.44 |  | 0.04 |
| 37 | Ethyl 2-bromopropionate | 30.36 | 30.32 | 30.31 | 0.05 |
| 38 | Propyl propylxanthate | 32.86 | 32.83 | 32.81 | 0.05 |
| 39 | 2-Chloroanisole | 38.60 | 38.17 | 38.65 | -0.05 |
| 40 | 1-Cyanooctadecane | 31.60 | 31.36 | 31.55 | 0.05 |
| 41 | Methyl cyclobutanecarboxylate | 30.79 | 30.89 | 30.84 | -0.05 |
| 42 | Diisopentyl acetylenedicarboxylate | 30.68 | 30.56 | 30.63 | 0.05 |
| 43 | Methylsalicylate | 39.80 | 39.79 | 39.85 | -0.05 |
| 44 | Octadecamethyloctasiloxane | 18.03 | 17.98 | 18.08 | -0.05 |
| 45 | 1-Hexyl-3-methylimidazolium L-alanine | 41.80 | 42.52 | 41.74 | 0.06 |
| 46 | Propyl 1-naphthylketone | 41.45 | 41.28 | 41.39 | 0.06 |
| 47 | Butyl 3-butoxypropanoate | 26.76 | 26.77 | 26.82 | -0.06 |
| 48 | Butyl butanoate | 24.75 | 24.75 | 24.81 | -0.06 |
| 49 | 3-(6-Hydroxyhexyl)-imidazolium chloride | 51.30 | 51.23 |  | 0.07 |
| 50 | n-Hexylamine | 26.22 | 25.72 | 26.15 | 0.07 |
| 51 | Phenylisothiocyanate | 40.56 | 40.49 |  | 0.07 |
| 52 | Tetradecamethylhexasiloxane | 17.42 | 17.34 | 17.35 | 0.07 |
| 53 | Isobutyl acetate | 23.06 | 23.23 | 23.13 | -0.07 |
| 54 | 1-Bromoundecane | 30.22 | 30.27 | 30.29 | -0.07 |
| 55 | 2,2,5-Trimethylhexane | 20.03 | 19.98 | 19.96 | 0.07 |
| 56 | 1-Heptanol | 26.71 | 26.56 | 26.64 | 0.07 |
| 57 | Butyl ethyl carbonate | 26.59 | 26.66 | 26.66 | -0.07 |
| 58 | 1,4-Diethylbenzene | 29.00 | 28.92 | 28.93 | 0.07 |
| 59 | 1-Methylcyclopentanol | 29.75 | 29.86 | 29.82 | -0.07 |
| 60 | 1-Bromononane | 29.57 | 29.87 | 29.64 | -0.07 |
| 61 | Butanal | 24.82 | 25.20 | 24.89 | -0.07 |
| 62 | 1,2,3,4-Tetrahydronaphthalene | 33.64 | 33.88 | 33.71 | -0.07 |
| 63 | 4,4-Dimethylheptane | 21.13 | 21.10 | 21.06 | 0.07 |
| 64 | Dibutyl carbonate | 27.19 | 27.06 | 27.26 | -0.07 |
| 65 | Propyl hydrocinnamate | 34.17 | 34.13 | 34.09 | 0.08 |
| 66 | 2-Butoxy-4-methyl-1,3,2-dioxaphospholane | 28.32 | 28.24 | 28.24 | 0.08 |
| 67 | Acetone O-propyloxime | 23.65 | 23.57 | 23.57 | 0.08 |
| 68 | 2-Methyl-2-heptanol | 25.24 | 25.30 | 25.32 | -0.08 |
| 69 | 6-Undecanone | 27.60 | 27.58 | 27.52 | 0.08 |
| 70 | 2-Chlorotoluene | 32.44 | 32.30 | 32.36 | 0.08 |
| 71 | 1,4-Dimethylbenzene | 28.55 | 28.48 | 28.47 | 0.08 |
| 72 | 1-Butyl-3-methylimidazolium acetate | 37.87 | 38.03 | 37.95 | -0.08 |
| 73 | Ethyl 2-cyano-3-propyl-2-pentenoate | 34.37 | 34.47 | 34.45 | -0.08 |
| 74 | 2-Butylnaphthalene | 35.32 | 35.56 | 35.23 | 0.09 |
| 75 | Ethyl butylxanthate | 32.90 | 32.83 | 32.81 | 0.09 |
| 76 | 4-Hydroxy-4-methyl-2-pentanone | 31.00 | 30.90 | 30.91 | 0.09 |
| 77 | Propyl isovalerate | 23.75 | 23.63 | 23.66 | 0.09 |
| 78 | Butyl propyl carbonate | 26.93 | 26.86 | 26.84 | 0.09 |
| 79 | Dipropyl butylphosphonate | 28.02 | 28.26 | 27.93 | 0.09 |
| 80 | 5-Dodecanol | 27.45 | 27.24 | 27.36 | 0.09 |
| 81 | Ethyl 2-cyano-3-methylvalerate | 30.81 | 30.89 | 30.72 | 0.09 |
| 82 | 3-Nitrotoluene | 40.99 | 40.08 | 40.90 | 0.09 |
| 83 | 3,5-Dimethylheptane | 20.90 | 20.92 | 20.99 | -0.09 |
| 84 | 2-Methyl-1-pentanol | 25.34 | 25.24 | 25.24 | 0.10 |
| 85 | Methyl cyclopropanecarboxylate | 30.79 | 30.69 | 30.69 | 0.10 |
| 86 | Propyl valerate | 24.79 | 24.75 | 24.69 | 0.10 |
| 87 | 4-(2,2,6-Trimethyl-1-cyclohexenyl)-2-butanol acetate | 29.67 | 29.67 | 29.77 | -0.10 |
| 88 | 3-(Diethylamino)propionitrile | 30.18 | 30.23 | 30.28 | -0.10 |
| 89 | 2-Ethoxy-4-methyl-1,3,2-dioxaphospholane | 27.75 | 27.84 | 27.85 | -0.10 |
| 90 | 2-Methyl-1-butanol | 25.10 | 25.04 | 24.99 | 0.11 |
| 91 | 2-Heptanol | 26.34 | 26.24 | 26.23 | 0.11 |
| 92 | 2-Octanol | 26.32 | 26.44 | 26.43 | -0.11 |
| 93 | Amyl acetate | 24.68 | 24.55 | 24.57 | 0.11 |
| 94 | (2-Iodoethyl)benzene | 40.71 | 40.87 | 40.82 | -0.11 |
| 95 | Tetraethylsilane | 23.06 | 22.95 |  | 0.11 |
| 96 | Diisopropyl succinate | 28.24 | 28.28 | 28.13 | 0.11 |
| 97 | Ethyl-n-hexylether | 22.08 | 22.40 | 22.19 | -0.11 |
| 98 | 2,3-Dimethylpentane | 19.97 | 20.08 | 20.08 | -0.11 |
| 99 | Dipropyl azodicarboxylate | 32.14 | 32.26 |  | -0.12 |
| 100 | p-Cymene | 28.10 | 27.80 | 27.98 | 0.12 |
| 101 | 1-Nitropropane | 29.85 | 30.09 | 29.73 | 0.12 |
| 102 | 3-Ethylhexane | 21.54 | 21.62 | 21.66 | -0.12 |
| 103 | Methyl (Z) 9-octadecenoate | 31.30 | 31.25 | 31.18 | 0.12 |
| 104 | 1-Pentyl-3-methylimidazolium tris(pentafluoroethyl)trifluorophosphate | 32.40 | 32.79 | 32.52 | -0.12 |
| 105 | Methyl 3-ethoxypropanoate | 28.71 | 28.47 | 28.58 | 0.13 |
| 106 | Tripropoxymethane | 24.80 | 24.67 |  | 0.13 |
| 107 | 2,5,8,11,14-Pentaoxopentadecane | 33.91 | 33.88 | 34.04 | -0.13 |
| 108 | Butyl 3-propoxypropanoate | 26.79 | 26.57 | 26.66 | 0.13 |
| 109 | Dipropyl propylphosphonate | 27.97 | 28.06 | 28.10 | -0.13 |
| 110 | 1-Butyl-3-methylimidazolium lactate | 44.00 | 43.69 | 43.87 | 0.13 |
| 111 | Ethyl hexanoate | 24.83 | 24.75 | 24.69 | 0.14 |
| 112 | Methyl 3-butenoate | 27.38 | 27.50 | 27.52 | -0.14 |
| 113 | Methyl beta-bromocinnamate | 44.36 | 44.50 |  | -0.14 |
| 114 | Dibutyl propylmalonate | 28.39 | 28.60 | 28.53 | -0.14 |
| 115 | 4-Ethyl-2-methylhexane | 20.85 | 20.92 | 20.99 | -0.14 |
| 116 | Butyl glycolate | 30.43 | 30.13 | 30.28 | 0.15 |
| 117 | Pentachloroethane | 34.14 | 34.29 | 34.29 | -0.15 |
| 118 | 2-(2-Pentyloxyethoxy)ethanol | 29.90 | 29.80 | 29.75 | 0.15 |
| 119 | 3,4-Dimethylhexane | 20.76 | 20.50 | 20.61 | 0.15 |
| 120 | Diethyl cyclopropane-1,1-dicarboxylate | 31.76 | 31.62 | 31.61 | 0.15 |
| 121 | 2-Chloro-2-methyloctane | 25.85 | 25.82 | 25.70 | 0.15 |
| 122 | 4-Propylpyridine | 32.08 | 32.37 | 32.23 | -0.15 |
| 123 | Allyl iodide | 31.75 | 31.94 | 31.91 | -0.16 |
| 124 | Ethyl 1,1-cyclopropanedicarboxylate | 32.91 | 33.05 | 33.07 | -0.16 |
| 125 | Ethyl mesaconate | 31.54 | 31.42 | 31.38 | 0.16 |
| 126 | 4-Ethyl-4-heptanol | 25.63 | 25.50 | 25.47 | 0.16 |
| 127 | 1,6-Hexanediol bis(perfluorobutyrate) | 22.20 | 21.92 | 22.03 | 0.17 |
| 128 | 3-Phenylpropene | 30.37 | 30.59 | 30.54 | -0.17 |
| 129 | 1,3-Diethylbenzene | 29.17 | 28.92 | 29.00 | 0.17 |
| 130 | Bromodiethylmethylsilane | 23.97 | 24.14 |  | -0.17 |
| 131 | Undecane | 24.21 | 24.00 | 24.04 | 0.17 |
| 132 | 4-Propyl-4-heptanol | 25.45 | 25.70 | 25.62 | -0.17 |
| 133 | 1-Chlorononane | 28.34 | 28.39 | 28.51 | -0.17 |
| 134 | 4-Methylanisole | 34.06 | 34.19 | 34.23 | -0.17 |
| 135 | 3-Ethyltoluene | 29.08 | 28.70 | 28.90 | 0.18 |
| 136 | 2-Pentanone ketazine | 27.01 | 26.66 | 26.83 | 0.18 |
| 137 | Carbitol | 29.53 | 29.20 | 29.35 | 0.18 |
| 138 | Methyl allylmalonate | 32.57 | 32.38 | 32.39 | 0.18 |
| 139 | Lauronitrile | 29.82 | 29.96 | 30.00 | -0.18 |
| 140 | Butyl propanoate | 24.39 | 24.55 | 24.57 | -0.18 |
| 141 | 2-(2-Octyloxyethoxy)ethanol | 30.70 | 30.40 | 30.52 | 0.18 |
| 142 | t-Butylbenzene | 28.13 | 28.10 | 27.95 | 0.18 |
| 143 | Ethyl allylmalonate | 29.65 | 29.88 | 29.83 | -0.18 |
| 144 | 3-Propylpyridine | 32.48 | 32.37 | 32.30 | 0.18 |
| 145 | 7-H-Perfluoroheptyl methyl ether | 22.10 | 22.83 | 22.29 | -0.19 |
| 146 | 1,1-Dibutylhydrazine | 26.17 | 25.98 |  | 0.19 |
| 147 | 1-Bromotetradecane | 31.17 | 30.87 | 30.98 | 0.19 |
| 148 | 3-Methyl-3-hexanol | 24.90 | 25.10 | 25.09 | -0.19 |
| 149 | 1-Butylnaphthalene | 35.69 | 35.56 | 35.50 | 0.19 |
| 150 | 2-Pentanone O-ethyloxime | 23.56 | 23.77 | 23.75 | -0.19 |
| 151 | Ethyl pentyl carbonate | 27.03 | 26.86 | 26.84 | 0.19 |
| 152 | 1,3-Dimethylbenzene | 28.47 | 28.48 | 28.66 | -0.19 |
| 153 | 3-Pentyloxypropanenitrile | 30.36 | 30.58 | 30.55 | -0.19 |
| 154 | Isopentyl formate | 24.89 | 24.74 | 24.70 | 0.19 |
| 155 | 2-Butanone O-ethyloxime | 23.75 | 23.57 | 23.56 | 0.19 |
| 156 | 1-Hexanol | 26.21 | 26.36 | 26.40 | -0.19 |
| 157 | Chlorobenzene | 32.99 | 33.34 | 33.19 | -0.20 |
| 158 | 1-Hexyl-3-methylimidazolium bis(trifluoromethanesulfonyl) amide | 32.31 | 32.54 | 32.51 | -0.20 |
| 159 | 1-Methyl-1-propylpyrrolidinium bis(trifluoromethanesulfonyl) amide | 32.30 | 32.32 | 32.10 | 0.20 |
| 160 | Diisopropyl oxalate | 28.43 | 27.88 | 28.23 | 0.20 |
| 161 | 2,2,3-Trimethylpentane | 19.77 | 19.56 | 19.57 | 0.20 |
| 162 | 2-Butanone O-propyloxime | 23.95 | 23.77 | 23.75 | 0.20 |
| 163 | Tetrahydrofuran | 26.40 | 26.56 | 26.60 | -0.20 |
| 164 | 1-Hexyl-3-methylimidazolium iodide | 43.50 | 43.70 | 43.70 | -0.20 |
| 165 | 1-Butyl-1-methylpyrrolidinium bis(trifluoromethylsulfonyl)amide | 32.30 | 32.52 | 32.51 | -0.21 |
| 166 | Aconitic acid triethylester | 34.56 | 34.36 | 34.35 | 0.21 |
| 167 | 4-Methyl-4-heptanol | 25.17 | 25.30 | 25.38 | -0.21 |
| 168 | (trans-1-Butenyl)benzene | 32.01 | 31.76 | 31.80 | 0.21 |
| 169 | Ethyl propyl carbonate | 26.60 | 26.46 | 26.39 | 0.21 |
| 170 | Butyl isobutanoate | 23.50 | 23.63 | 23.71 | -0.21 |
| 171 | Ethyl acrylate | 24.66 | 24.80 | 24.87 | -0.21 |
| 172 | Triisopropyl phosphate | 27.96 | 27.84 | 28.17 | -0.21 |
| 173 | 5-Methyl-5-decanol | 26.14 | 25.90 | 25.92 | 0.22 |
| 174 | 3-Ethylheptane | 21.89 | 22.04 | 22.11 | -0.22 |
| 175 | Ethyl fumarate | 31.79 | 31.55 | 31.57 | 0.22 |
| 176 | Diethylsulfoxide | 34.60 | 34.82 |  | -0.22 |
| 177 | 3-Methyl-4-(2,6,6-trimethyl-2-cyclohexen-1-yl)-3-buten-2-one | 32.17 | 32.50 | 32.40 | -0.23 |
| 178 | 2-Methyloctane | 21.88 | 22.04 | 22.11 | -0.23 |
| 179 | 2-Nitropropane | 29.87 | 29.88 | 29.64 | 0.23 |
| 180 | cis-1,2-Dimethylcyclohexane | 25.20 | 25.46 | 25.43 | -0.23 |
| 181 | 4-Methyloctane | 22.34 | 22.04 | 22.11 | 0.23 |
| 182 | Dibutyl ethylphosphonate | 28.05 | 28.26 | 28.28 | -0.23 |
| 183 | Propyl 3-methoxypropanoate | 28.42 | 28.67 | 28.65 | -0.23 |
| 184 | alpha-Ionone | 32.20 | 32.63 | 32.44 | -0.24 |
| 185 | Hexyl 3-propoxypropanoate | 27.32 | 26.97 | 27.08 | 0.24 |
| 186 | 1-Fluorohexane | 21.41 | 21.21 | 21.17 | 0.24 |
| 187 | Decane | 23.39 | 23.58 | 23.63 | -0.24 |
| 188 | Hexylbenzene | 30.14 | 30.60 | 30.38 | -0.24 |
| 189 | Dipentyl sulfide | 27.80 | 27.68 | 28.04 | -0.24 |
| 190 | 2,11-Dichloro-2,11-dimethyldodecane | 31.19 | 31.46 | 31.43 | -0.24 |
| 191 | 2-Pentoxy-4-methyl-1,3,2-dioxaphospholane | 28.24 | 28.44 | 28.48 | -0.24 |
| 192 | Methyl crotonate | 28.62 | 28.45 | 28.38 | 0.24 |
| 193 | Tetracosane | 29.29 | 29.46 | 29.53 | -0.24 |
| 194 | Methyl 3-methyl-3-propylglutarate | 31.20 | 31.39 | 31.44 | -0.24 |
| 195 | Dibutyl maleate | 30.73 | 30.92 | 30.97 | -0.24 |
| 196 | Propyl 2-thiophenecarboxylate | 34.06 | 34.32 | 34.30 | -0.24 |
| 197 | 1-Ethyl-3-methylimidazolium diethylphosphate | 37.10 | 36.90 | 36.85 | 0.25 |
| 198 | 1-Chlorodecane | 28.89 | 28.59 | 28.64 | 0.25 |
| 199 | Allyl cyanide | 29.23 | 29.01 | 28.98 | 0.25 |
| 200 | Dipropyl carbonate | 26.91 | 26.66 | 26.66 | 0.25 |
| 201 | Propyl phenylpropiolate | 37.07 | 37.21 | 37.32 | -0.25 |
| 202 | 4-Ethylheptane | 21.89 | 22.04 | 22.14 | -0.25 |
| 203 | Nonane | 22.96 | 23.16 | 23.21 | -0.25 |
| 204 | 1-Butyl-3-methylimidazolium tris(pentafluoroethyl)trifluorophosphate | 32.58 | 32.59 | 32.33 | 0.25 |
| 205 | Triethyl nitrilotriacetate | 34.67 | 34.36 | 34.41 | 0.26 |
| 206 | Isobutylcyclohexane | 26.51 | 26.30 | 26.25 | 0.26 |
| 207 | 1,4-Dichlorobutane | 34.80 | 34.60 | 34.54 | 0.26 |
| 208 | 2-Hexoxy-4-methyl-1,3,2-dioxaphospholane | 28.39 | 28.64 | 28.65 | -0.26 |
| 209 | Dibutyldisulfide | 30.55 | 30.56 | 30.81 | -0.26 |
| 210 | N-Butyl-3-methylpyridinium bis(trifluoromethylsulfonyl)amide | 33.60 | 33.60 | 33.87 | -0.27 |
| 211 | Isoamyl acetate | 23.78 | 23.43 | 23.51 | 0.27 |
| 212 | 2-Ethylthiophene | 29.86 | 30.15 | 30.13 | -0.27 |
| 213 | n-Decyl alcohol | 27.32 | 27.16 | 27.05 | 0.27 |
| 214 | 3-Nonanone | 27.43 | 27.18 | 27.16 | 0.27 |
| 215 | Ethyl propanoate | 23.80 | 24.15 | 24.07 | -0.27 |
| 216 | Bis(7-H-perfluoroheptyl) adipate | 26.10 | 26.38 | 26.37 | -0.27 |
| 217 | Diethylene glycol monohexyl ether | 29.60 | 30.00 | 29.87 | -0.27 |
| 218 | Methyl 3-ethyl-3-propylglutarate | 31.38 | 31.59 | 31.65 | -0.27 |
| 219 | alpha-Methylbenzenepropanol | 35.73 | 35.42 | 35.45 | 0.28 |
| 220 | Dimethyldiphenylsilane | 32.91 | 33.19 |  | -0.28 |
| 221 | Butyl pentanoate | 25.22 | 24.95 | 24.94 | 0.28 |
| 222 | 1-Butyl-2,3-dimethylimidazolium hexafluorophosphate | 44.42 | 44.14 |  | 0.28 |
| 223 | 1-Octanol | 27.10 | 26.76 | 26.82 | 0.28 |
| 224 | 2,7-Dimethyloctane | 21.60 | 21.34 | 21.32 | 0.28 |
| 225 | Pentyl 3-propoxypropanoate | 27.10 | 26.77 | 26.82 | 0.28 |
| 226 | Heptadecanonitrile | 31.37 | 30.96 | 31.09 | 0.28 |
| 227 | Bis(5-H-perfluoropentyl) adipate | 27.70 | 27.98 | 27.98 | -0.28 |
| 228 | Amylamine | 25.20 | 25.52 | 25.48 | -0.28 |
| 229 | Ethyl 2-cyano-3-methyl-2-pentenoate | 33.86 | 34.07 | 34.14 | -0.28 |
| 230 | 1-Butyl-3-methylimidazolium glycine | 43.50 | 43.17 | 43.22 | 0.28 |
| 231 | 1,1-Dipropoxyethane | 23.09 | 22.80 | 22.81 | 0.28 |
| 232 | n-Hexylformate | 25.89 | 26.06 | 26.17 | -0.28 |
| 233 | Bis(7-H-perfluoroheptyl) 3-methylglutarate | 25.60 | 25.26 | 25.31 | 0.29 |
| 234 | Ethyl isovalerate | 23.78 | 23.43 | 23.49 | 0.29 |
| 235 | 3-Octanone | 26.68 | 26.98 | 26.97 | -0.29 |
| 236 | Diisobutylsulfide | 24.91 | 25.04 | 24.62 | 0.29 |
| 237 | Propyl 3-pentyloxypropanoate | 27.00 | 26.77 | 26.71 | 0.29 |
| 238 | 3-Methyloctane | 22.34 | 22.04 | 22.05 | 0.29 |
| 239 | Cyclohexyl ethyl ether | 27.29 | 27.04 | 27.00 | 0.29 |
| 240 | Triheptyl borate | 26.15 | 25.78 | 25.86 | 0.29 |
| 241 | 1-Butyl-1-methylpyrrolidinium trifluoroacetate | 35.70 | 35.71 | 35.99 | -0.29 |
| 242 | Dipropyl octylphosphonate | 28.77 | 29.06 | 29.06 | -0.29 |
| 243 | beta-Ionone | 33.46 | 33.42 | 33.16 | 0.30 |
| 244 | Trimethylisoxazole | 33.56 | 33.86 |  | -0.30 |
| 245 | Diisobutyl disulfide | 27.97 | 28.32 | 28.27 | -0.30 |
| 246 | Methyl 1,1-cyclohexanedicarboxylate | 35.80 | 36.15 | 36.10 | -0.30 |
| 247 | 4-Butylpyridine | 32.37 | 32.57 | 32.67 | -0.30 |
| 248 | 2-Pentyloxyethanol | 27.60 | 27.98 | 27.90 | -0.30 |
| 249 | Glycerol trinitrate | 50.73 | 50.02 | 50.42 | 0.31 |
| 250 | Octadecanenitrile | 31.50 | 31.16 | 31.19 | 0.31 |
| 251 | Diisopentyl disulfide | 28.46 | 28.72 | 28.77 | -0.31 |
| 252 | Phenyl n-propyl sulfide | 35.37 | 35.60 | 35.68 | -0.31 |
| 253 | Butyl isovalerate | 24.22 | 23.83 | 23.91 | 0.31 |
| 254 | Docosane | 28.97 | 28.62 | 28.66 | 0.31 |
| 255 | Dihexyl carbonate | 28.06 | 27.86 | 27.75 | 0.31 |
| 256 | Methyl 3-methoxypropanoate | 30.83 | 30.97 | 31.14 | -0.31 |
| 257 | 2-Dodecyloxyethanol | 29.60 | 29.38 | 29.29 | 0.31 |
| 258 | 1-Bromohexadecane | 31.65 | 31.27 | 31.33 | 0.32 |
| 259 | 3-Cyclohexylpropyne | 29.81 | 30.30 | 30.13 | -0.32 |
| 260 | Ethyl 2-cyano-3-methylbutyrate | 30.47 | 30.69 | 30.79 | -0.32 |
| 261 | 2-Propyn-1-butyrate | 30.04 | 29.87 | 29.72 | 0.32 |
| 262 | Butylcyclohexane | 27.03 | 27.42 | 27.35 | -0.32 |
| 263 | n-Butylbenzoate | 33.67 | 33.93 | 33.99 | -0.32 |
| 264 | Dimethyl chlorofumarate | 37.52 | 37.45 | 37.84 | -0.32 |
| 265 | Methacrylic acid | 26.50 | 26.10 | 26.17 | 0.33 |
| 266 | 2,2-Dimethylheptane | 20.89 | 21.10 | 21.22 | -0.33 |
| 267 | Pentyl butyrate | 25.20 | 24.95 | 24.87 | 0.33 |
| 268 | Ethyl butanoate | 23.94 | 24.35 | 24.27 | -0.33 |
| 269 | 2-Heptanone | 26.65 | 26.78 | 26.98 | -0.33 |
| 270 | Dipropyl thiosulfate | 29.60 | 29.35 | 29.27 | 0.33 |
| 271 | Decanenitrile | 29.36 | 29.56 | 29.69 | -0.33 |
| 272 | Propyl 2-naphthoate | 38.59 | 39.05 | 38.92 | -0.33 |
| 273 | Isopropylcyclohexane | 25.50 | 25.88 | 25.83 | -0.33 |
| 274 | 1-Hexyl-3-methylimidazolium hexafluorophosphate | 39.02 | 38.78 | 38.69 | 0.33 |
| 275 | 3-Ethyl-3-methylpentane | 21.08 | 20.68 | 20.74 | 0.34 |
| 276 | Tripropyl phosphate | 29.08 | 28.80 | 28.74 | 0.34 |
| 277 | Ethyl 3-hydroxyatropate | 37.67 | 38.01 | 38.01 | -0.34 |
| 278 | Butyrophenone | 35.49 | 35.96 | 35.83 | -0.34 |
| 279 | N,N-Diisobutylaniline | 30.75 | 30.58 | 30.41 | 0.34 |
| 280 | Dipropyl propylmalonate | 28.68 | 28.20 | 28.34 | 0.34 |
| 281 | Ethyl pentanoate | 24.14 | 24.55 | 24.48 | -0.34 |
| 282 | 1-Phenyl-3-pentanone | 36.52 | 36.16 | 36.17 | 0.35 |
| 283 | 2-Ethylpyridine | 32.01 | 32.41 | 32.36 | -0.35 |
| 284 | 1-Bromooctadecane | 32.01 | 31.67 | 31.66 | 0.35 |
| 285 | Tributoxymethane | 25.62 | 25.27 |  | 0.35 |
| 286 | Tripropylamine | 22.82 | 22.45 | 22.46 | 0.36 |
| 287 | Butyl cyclopropanecarboxylate | 28.91 | 28.59 | 28.55 | 0.36 |
| 288 | 1,2-Dibromopropane | 34.50 | 34.84 | 34.86 | -0.36 |
| 289 | 9-H-Perfluorononyl butyrate | 22.70 | 22.28 | 22.34 | 0.36 |
| 290 | Dibutyl ethylmalonate | 28.79 | 28.40 | 28.43 | 0.36 |
| 291 | 2,3-Dimethyl-2-butanol | 24.24 | 23.78 | 23.88 | 0.36 |
| 292 | Methyl 3,3-diethylglutarate | 31.74 | 31.39 | 31.38 | 0.36 |
| 293 | 2-Butanone ketazine | 26.58 | 26.26 | 26.22 | 0.36 |
| 294 | 3-Methylheptane | 21.31 | 21.62 | 21.67 | -0.36 |
| 295 | Dipropyl heptylphosphonate | 28.54 | 28.86 | 28.90 | -0.36 |
| 296 | Butyl ethylxanthate | 32.78 | 32.83 | 32.41 | 0.37 |
| 297 | Ethyl 3-butynoate | 30.43 | 29.67 | 30.06 | 0.37 |
| 298 | Iodobenzene | 38.71 | 39.08 |  | -0.37 |
| 299 | 2,4,4-Trimethylhexane | 20.33 | 19.98 | 19.96 | 0.37 |
| 300 | 1-Bromododecane | 30.83 | 30.47 | 30.46 | 0.37 |
| 301 | Pentyl cyclopropanecarboxylate | 29.12 | 28.79 | 28.75 | 0.37 |
| 302 | Dipropyl pentylphosphonate | 28.20 | 28.46 | 28.57 | -0.37 |
| 303 | Decylcyclohexane | 29.77 | 29.94 | 30.14 | -0.37 |
| 304 | 1-Bromo-4-fluorobenzene | 34.68 | 35.07 | 35.05 | -0.37 |
| 305 | 1,3,5-Trimethylbenzene | 28.00 | 27.46 | 27.62 | 0.38 |
| 306 | Pentyl cyclobutanecarboxylate | 29.34 | 28.99 | 28.96 | 0.38 |
| 307 | beta-Isobutyl-alpha-methylstyrene | 31.38 | 30.97 | 31.00 | 0.38 |
| 308 | N,N-Dipropylaniline | 32.92 | 32.42 | 32.54 | 0.38 |
| 309 | Methyl 3,3-dimethylglutarate | 31.42 | 30.99 | 31.04 | 0.38 |
| 310 | 1-Butanethiol | 25.22 | 25.69 | 25.60 | -0.38 |
| 311 | 2-Propylnaphthalene | 35.70 | 35.34 | 35.31 | 0.39 |
| 312 | 2,2,4-Trimethylhexane | 19.67 | 19.98 | 20.06 | -0.39 |
| 313 | 1,5-Pentanediol bis(perfluorooctanoate) | 19.10 | 18.52 | 18.71 | 0.39 |
| 314 | Isobutyl valerate | 23.40 | 23.83 | 23.79 | -0.39 |
| 315 | 2-Octanone | 26.79 | 26.98 | 27.18 | -0.39 |
| 316 | 2,3-Dimethylhexane | 20.09 | 20.50 | 20.48 | -0.39 |
| 317 | 2-Methyl-2-octanol | 25.84 | 25.50 | 25.45 | 0.39 |
| 318 | 4-(2,2,6-Trimethyl-1-cyclohexenyl)-2-butanol | 31.68 | 31.28 | 31.29 | 0.39 |
| 319 | 6-Methyl-6-dodecanol | 26.07 | 26.30 | 26.46 | -0.39 |
| 320 | Pentyl 3-pentyloxypropanoate | 27.68 | 27.17 | 27.29 | 0.39 |
| 321 | N-Propyl-2-methylpyridinium bis(trifluoromethylsulfonyl)amide | 36.89 | 37.28 |  | -0.39 |
| 322 | Dimethyl ethylmalonate | 32.38 | 32.60 | 32.77 | -0.39 |
| 323 | Anisole | 35.70 | 35.23 | 35.30 | 0.40 |
| 324 | Dimethyl phthalate | 41.85 | 41.66 | 41.45 | 0.40 |
| 325 | 1,10-Decanediol bis(perfluorobutyrate) | 23.00 | 22.72 | 22.60 | 0.40 |
| 326 | n-Propyl acetate | 23.80 | 24.15 | 24.20 | -0.40 |
| 327 | Propyl phenylacetate | 34.29 | 33.93 | 33.89 | 0.40 |
| 328 | 4-Fluorotoluene | 28.22 | 27.95 | 27.82 | 0.40 |
| 329 | t-Butylmercaptan | 20.51 | 20.91 |  | -0.40 |
| 330 | Diethyl 1,1-cyclobutanedicarboxylate | 31.54 | 31.82 | 31.94 | -0.40 |
| 331 | 1,1,2-Trichloroethane | 34.02 | 34.62 | 34.42 | -0.40 |
| 332 | 4-(2-Thienyl)-1-butanol | 36.27 | 36.33 | 36.67 | -0.40 |
| 333 | 3-Tolunitrile | 36.82 | 36.50 | 36.41 | 0.41 |
| 334 | Butyl 3-pentyloxypropanoate | 27.35 | 26.97 | 26.94 | 0.41 |
| 335 | Diethyl pentylmalonate | 28.53 | 28.20 | 28.12 | 0.41 |
| 336 | 1-Butyl-3-methylimidazolium tetrafluoroborate | 44.80 | 44.97 | 44.39 | 0.41 |
| 337 | 2-n-Hexoxyethanol | 27.70 | 28.18 | 28.11 | -0.41 |
| 338 | 4-(2,2,6-Trimethyl-2-cyclohexenyl)-2-butanol | 30.89 | 30.49 | 30.48 | 0.41 |
| 339 | 5-Nonanone | 26.77 | 27.18 | 27.18 | -0.41 |
| 340 | Dipropyl ethylphosphonate | 28.25 | 27.86 | 27.84 | 0.41 |
| 341 | Methylacetoacetate | 33.09 | 32.85 | 32.68 | 0.41 |
| 342 | Thiophene | 31.34 | 31.03 | 30.93 | 0.41 |
| 343 | Di-n-butylsulfide | 27.23 | 27.28 | 27.64 | -0.41 |
| 344 | 2-Propyn-1-valerate | 29.80 | 30.07 | 30.21 | -0.41 |
| 345 | Tridecanonitrile | 30.70 | 30.16 | 30.29 | 0.41 |
| 346 | Methyl butoxyacetate | 28.12 | 28.67 | 28.53 | -0.41 |
| 347 | N-Propylaniline | 34.79 | 35.22 | 35.20 | -0.41 |
| 348 | 4-Pentylpyridine | 32.41 | 32.77 | 32.83 | -0.42 |
| 349 | 2,6-Dimethylheptan-4-one | 25.54 | 24.94 | 25.12 | 0.42 |
| 350 | 1,1-Diethylhydrazine | 24.87 | 25.18 | 25.29 | -0.42 |
| 351 | Methyl 3-propoxypropanoate | 28.26 | 28.67 | 28.68 | -0.42 |
| 352 | Propyl 1-naphthoate | 39.60 | 39.05 | 39.18 | 0.42 |
| 353 | Di-n-pentyl oxalate | 29.67 | 29.32 | 29.24 | 0.43 |
| 354 | 1-Pentanethiol | 26.35 | 25.89 | 25.92 | 0.43 |
| 355 | 1-Pentanol | 25.79 | 26.16 | 26.22 | -0.43 |
| 356 | Di-n-butylether | 22.91 | 22.40 | 22.48 | 0.43 |
| 357 | 3-Methyl-3-heptanol | 25.68 | 25.30 | 25.25 | 0.43 |
| 358 | 3-Chloro-3-ethylhexane | 26.02 | 25.62 | 25.59 | 0.43 |
| 359 | Diisopentyl maleate | 28.60 | 29.08 | 29.03 | -0.43 |
| 360 | 3-Hexyloxypropanenitrile | 30.33 | 30.78 | 30.76 | -0.43 |
| 361 | Methyl trichloroacetate | 33.13 | 33.33 | 33.56 | -0.43 |
| 362 | 4-Methylheptane | 21.12 | 21.62 | 21.56 | -0.44 |
| 363 | Dipropyl disulfide | 30.53 | 30.16 | 30.09 | 0.44 |
| 364 | 1-Chloroundecane | 29.30 | 28.79 | 28.86 | 0.44 |
| 365 | 3-Hexanone O-ethyloxime | 24.25 | 23.97 | 23.81 | 0.44 |
| 366 | 2,4-Dimethylhexane | 20.05 | 20.50 | 20.49 | -0.44 |
| 367 | Butyl 3-ethoxypropanoate | 26.78 | 26.37 | 26.33 | 0.45 |
| 368 | 5-Propyl-5-decanol | 26.70 | 26.30 | 26.25 | 0.45 |
| 369 | Propyl butyrate | 25.06 | 24.55 | 24.61 | 0.45 |
| 370 | beta-Isopropylstyrene | 31.40 | 30.86 | 30.95 | 0.45 |
| 371 | 1-Butoxy-1-ethoxyethane | 23.26 | 22.80 | 22.81 | 0.45 |
| 372 | 1,8-Diiodooctane | 43.25 | 43.60 | 43.70 | -0.45 |
| 373 | cis-1,2-Dichloroethylene | 26.40 | 25.94 |  | 0.46 |
| 374 | 3-Butoxypropanenitrile | 29.90 | 30.38 | 30.36 | -0.46 |
| 375 | Di-n-butylsulfite | 28.81 | 29.09 | 29.27 | -0.46 |
| 376 | 4-Methylcyclohexanone | 30.96 | 30.62 | 30.50 | 0.46 |
| 377 | Propyl nicotinate | 36.11 | 36.34 | 36.57 | -0.46 |
| 378 | 3-Heptanone | 26.30 | 26.78 | 26.76 | -0.46 |
| 379 | Propyl 2-naphthaleneacetate | 38.81 | 39.25 | 39.27 | -0.46 |
| 380 | Bicyclohexyl | 32.74 | 32.10 | 32.27 | 0.47 |
| 381 | 5-Methyl-5-undecanol | 26.54 | 26.10 | 26.07 | 0.47 |
| 382 | Dibutyl methylmalonate | 28.81 | 28.20 | 28.34 | 0.47 |
| 383 | Ethyl orthoformate | 23.60 | 24.07 |  | -0.47 |
| 384 | Hexyleneglycol | 33.10 | 33.62 | 33.57 | -0.47 |
| 385 | 3,3-Dimethylhexane | 20.17 | 20.68 | 20.64 | -0.47 |
| 386 | n-Propylchloroacetate | 30.74 | 31.36 | 31.21 | -0.47 |
| 387 | 2-Butyrylthiophene | 36.78 | 36.55 | 36.30 | 0.48 |
| 388 | 4-Methyl-4-nonanol | 26.15 | 25.70 | 25.67 | 0.48 |
| 389 | Hexyl 3-butoxypropanoate | 27.74 | 27.17 | 27.26 | 0.48 |
| 390 | Bis(perfluorobutyl) sebacate | 22.40 | 22.86 | 22.88 | -0.48 |
| 391 | 4-Chloro-4-methylheptane | 25.22 | 25.62 | 25.70 | -0.48 |
| 392 | 4-Chloro-4-ethylheptane | 26.30 | 25.82 | 25.82 | 0.48 |
| 393 | Butyl 3-methoxypropanoate | 28.48 | 28.87 | 28.96 | -0.48 |
| 394 | Methylenecyclohexane | 25.69 | 26.20 | 26.17 | -0.48 |
| 395 | Hexachlorobutadiene | 36.00 | 36.48 |  | -0.48 |
| 396 | 1,2-Dimethoxycyclohexane | 34.20 | 33.54 | 33.71 | 0.49 |
| 397 | Isovaleraldehyde | 23.69 | 24.28 | 24.18 | -0.49 |
| 398 | 1,3-Dipentylimidazolium bis(trifluoromethylsulfonyl) amide | 30.10 | 29.94 | 29.61 | 0.49 |
| 399 | Methyl decanoate | 27.59 | 28.05 | 28.08 | -0.49 |
| 400 | Decylcyclopentane | 29.34 | 29.52 | 29.83 | -0.49 |
| 401 | Pentyl 3-methoxypropanoate | 28.59 | 29.07 | 29.08 | -0.49 |
| 402 | 4-Chloro-4-propylheptane | 26.40 | 26.02 | 25.90 | 0.50 |
| 403 | 1-Phenyl-2-butanone | 36.57 | 35.96 | 36.07 | 0.50 |
| 404 | 3-Decanone | 27.82 | 27.38 | 27.32 | 0.50 |
| 405 | Triptane | 18.75 | 19.14 | 19.25 | -0.50 |
| 406 | Dodecane | 24.93 | 24.42 | 24.43 | 0.50 |
| 407 | (2-Methyl-1-propenyl)benzene | 31.86 | 31.43 | 31.36 | 0.50 |
| 408 | Tris(2-propoxyethyl) phosphite | 28.94 | 29.46 | 29.44 | -0.50 |
| 409 | Dipentyl phosphite | 28.51 | 28.98 | 29.01 | -0.50 |
| 410 | Diisopropyl acetylenedicarboxylate | 30.67 | 31.36 | 31.17 | -0.50 |
| 411 | beta,beta-Diethylstyrene | 32.22 | 31.87 | 31.71 | 0.51 |
| 412 | Isopentylamine | 23.81 | 24.40 | 24.32 | -0.51 |
| 413 | 1,1-Di(2-chloroethoxy)ethane | 36.22 | 36.82 | 36.73 | -0.51 |
| 414 | Di-n-hexylether | 23.78 | 23.20 | 23.26 | 0.52 |
| 415 | Butylmethylketone | 26.00 | 26.58 | 26.52 | -0.52 |
| 416 | Hexacosane | 29.89 | 30.30 | 30.41 | -0.52 |
| 417 | 4-Nonanone | 26.76 | 27.18 | 27.28 | -0.52 |
| 418 | Hexyl 3-methoxypropanoate | 28.76 | 29.27 | 29.28 | -0.52 |
| 419 | 1-Pentyl-3-methylimidazolium tetracyanoborate | 43.10 | 42.71 | 42.58 | 0.52 |
| 420 | 2-Pentanone oxime | 28.90 | 29.00 | 29.42 | -0.52 |
| 421 | Dihexyloxymethane | 27.02 | 25.36 | 26.49 | 0.53 |
| 422 | 2-Methyl-3-pentanol | 24.60 | 24.92 | 25.13 | -0.53 |
| 423 | 2,5-Dimethylheptane | 20.42 | 20.92 | 20.95 | -0.53 |
| 424 | 1,3-Dichloropropane | 33.93 | 34.40 | 34.46 | -0.53 |
| 425 | 1-Phenyldodecane | 31.60 | 31.92 | 32.13 | -0.53 |
| 426 | Methyl dimethylmalonate | 31.04 | 30.59 | 30.50 | 0.54 |
| 427 | 2,10-Dichloro-2,10-dimethylundecane | 30.78 | 31.26 | 31.32 | -0.54 |
| 428 | 2,6-Lutidine | 31.65 | 31.41 | 31.11 | 0.54 |
| 429 | Allyl phenyl ether | 33.90 | 33.58 | 33.36 | 0.54 |
| 430 | Butyl 3-Heptyloxypropanoate | 28.01 | 27.37 | 27.47 | 0.54 |
| 431 | Amyl formate | 25.02 | 25.86 | 25.56 | -0.54 |
| 432 | 4-Methyl-4-decanol | 26.47 | 25.90 | 25.92 | 0.55 |
| 433 | Hexyl 3-pentyloxypropanoate | 27.94 | 27.37 | 27.39 | 0.55 |
| 434 | 2,3-Dimethylheptane | 21.46 | 20.92 | 20.91 | 0.55 |
| 435 | 1-Nitrobutane | 29.77 | 30.29 | 30.32 | -0.55 |
| 436 | s-Butylbenzene | 28.52 | 29.04 | 29.07 | -0.55 |
| 437 | Pentyl 3-butoxypropanoate | 27.41 | 26.97 | 26.86 | 0.55 |
| 438 | Bis(perfluorobutyl) 3-methylglutarate | 20.50 | 20.94 | 21.05 | -0.55 |
| 439 | 1,1-Dipropylhydrazine | 25.16 | 25.58 | 25.71 | -0.55 |
| 440 | 3-(2-Thienyl)-1-propanol | 36.77 | 36.13 | 36.22 | 0.55 |
| 441 | Ethyl acetate | 23.39 | 23.95 | 23.94 | -0.55 |
| 442 | 1-Methylcyclohexene | 26.60 | 27.15 | 27.15 | -0.55 |
| 443 | Heptyl butoxyacetate | 27.75 | 27.17 | 27.19 | 0.56 |
| 444 | Di-n-pentyl succinate | 30.27 | 29.72 | 29.71 | 0.56 |
| 445 | 1-Dodecyne | 27.70 | 27.34 | 27.14 | 0.56 |
| 446 | Caprylene | 21.28 | 21.79 | 21.84 | -0.56 |
| 447 | Dibutyl pentylmalonate | 28.44 | 29.00 | 29.00 | -0.56 |
| 448 | 4-Methylvaleronitrile | 27.06 | 27.84 | 27.62 | -0.56 |
| 449 | Dipropyl sulfide | 26.29 | 26.88 | 26.85 | -0.56 |
| 450 | 4-Chlorotoluene | 32.77 | 32.30 | 32.20 | 0.57 |
| 451 | Propyl 3-propoxypropanoate | 26.99 | 26.37 | 26.42 | 0.57 |
| 452 | Butyl ethyl sulfide | 26.47 | 26.88 | 27.04 | -0.57 |
| 453 | Hexyl butoxyacetate | 27.59 | 26.97 | 27.02 | 0.57 |
| 454 | 1,1-Dipentylhydrazine | 26.95 | 26.38 |  | 0.57 |
| 455 | 1-m-tolyl-1-o-tolylethane | 36.10 | 36.46 | 36.67 | -0.57 |
| 456 | Pentyl 3-ethoxypropanoate | 27.23 | 26.57 | 26.65 | 0.58 |
| 457 | Ethyl 3-Heptyloxypropanoate | 27.57 | 26.97 | 26.99 | 0.58 |
| 458 | Butyl cyclobutanecarboxylate | 29.35 | 28.79 | 28.77 | 0.58 |
| 459 | 4-Heptanone O-ethyloxime | 24.76 | 24.17 | 24.17 | 0.59 |
| 460 | n-Valeraldehyde | 25.94 | 25.40 | 25.35 | 0.59 |
| 461 | Ethylbenzene | 29.29 | 29.72 | 29.88 | -0.59 |
| 462 | Methyl 3-butoxypropanoate | 28.27 | 28.87 | 28.86 | -0.59 |
| 463 | 1,3-Dihexylimidazolium bis(trifluoromethylsulfonyl) amide | 29.50 | 30.34 | 30.09 | -0.59 |
| 464 | 1-Butanol | 25.39 | 25.96 | 25.98 | -0.59 |
| 465 | 2,3,5-Trimethylhexane | 20.38 | 19.80 | 19.79 | 0.59 |
| 466 | Butyl butoxyacetate | 27.13 | 26.57 | 26.54 | 0.59 |
| 467 | Propyl fumarate | 31.01 | 31.75 | 31.60 | -0.59 |
| 468 | 2,4-Dimethylheptane | 20.42 | 20.92 | 21.01 | -0.59 |
| 469 | Butyl 3-butynoate | 29.42 | 30.07 | 30.01 | -0.59 |
| 470 | 3-Chloro-3-methylhexane | 24.83 | 25.42 | 25.42 | -0.59 |
| 471 | Ethyl 3,3-dimethylcyclopropane-trans-1,2-dicarboxylate | 30.65 | 31.21 | 31.24 | -0.59 |
| 472 | 1-Bromo-2-ethoxyethane | 29.72 | 30.29 | 30.31 | -0.59 |
| 473 | 1-Hexyl-3-methylimidazolium tetracyanoborate | 41.50 | 42.91 | 42.09 | -0.59 |
| 474 | 4-Methyl-3-penten-2-one oxime | 30.84 | 30.47 | 30.24 | 0.60 |
| 475 | Propyl 3-Hexyloxypropanoate | 27.59 | 26.97 | 26.99 | 0.60 |
| 476 | 5-Chloro-5-methylnonane | 26.70 | 26.02 | 26.10 | 0.60 |
| 477 | Triethyl phosphite | 23.95 | 23.40 | 23.35 | 0.60 |
| 478 | 3-Methylisoxazole | 35.13 | 34.81 | 34.53 | 0.60 |
| 479 | Methyl dichloroacetate | 34.56 | 34.08 | 33.96 | 0.60 |
| 480 | 4-Methylpent-3-en-2-one | 28.40 | 27.85 | 27.80 | 0.60 |
| 481 | 2-Methoxy-4-methyl-1,3,2-dioxaphospholane | 29.65 | 30.34 | 30.25 | -0.60 |
| 482 | 4-Ethyltoluene | 29.44 | 28.70 | 28.84 | 0.60 |
| 483 | 3-Methylcyclohexanone | 31.21 | 30.62 | 30.61 | 0.60 |
| 484 | 3-Hexanone oxime | 28.70 | 29.20 | 29.30 | -0.60 |
| 485 | 3-Propoxypropanenitrile | 29.75 | 30.18 | 30.35 | -0.60 |
| 486 | 1,7-Diiodoheptane | 43.93 | 43.40 | 43.33 | 0.60 |
| 487 | Bromodimethylphenylsilane | 28.65 | 29.26 |  | -0.61 |
| 488 | Bis(2-propoxyethyl)phosphite | 30.97 | 31.82 | 31.58 | -0.61 |
| 489 | 1-Heptyl-3-methylimidazolium bis(trifluoromethanesulfonyl) amide | 32.00 | 32.74 | 32.62 | -0.62 |
| 490 | 1-Pentyl-3-methylimidazolium bis(trifluoromethanesulfonyl) amide | 32.90 | 32.34 | 32.28 | 0.62 |
| 491 | 3-Methylbutanoic acid | 25.51 | 24.86 | 24.89 | 0.62 |
| 492 | Diisobutylsulfite | 27.10 | 26.85 | 26.48 | 0.62 |
| 493 | Cumene | 28.20 | 28.82 | 28.82 | -0.62 |
| 494 | 3-Hexanone ketazine | 26.61 | 27.06 | 27.23 | -0.62 |
| 495 | Dipropylamine | 22.82 | 23.46 | 23.44 | -0.62 |
| 496 | Dibutyl thiosulfate | 29.28 | 29.75 | 29.90 | -0.62 |
| 497 | 1-Pentylnaphthalene | 35.18 | 35.78 | 35.80 | -0.62 |
| 498 | 1-Butyl-3-methylimidazolium thiocyanate | 47.45 | 47.98 | 48.08 | -0.63 |
| 499 | 2-Pentylthiophene | 30.09 | 30.75 | 30.72 | -0.63 |
| 500 | Ethyl heptanoate | 25.50 | 24.95 | 24.87 | 0.63 |
| 501 | Triethylmethanol | 25.68 | 25.10 | 25.05 | 0.63 |
| 502 | 1-Bromodecane | 29.55 | 30.07 | 30.18 | -0.63 |
| 503 | 2-Methyl-1-propanethiol | 24.01 | 24.57 | 24.64 | -0.63 |
| 504 | Ethyl 2-cyano-3,3-diethylacrylate | 33.51 | 34.27 | 34.14 | -0.63 |
| 505 | Bis(5-H-perfluoropentyl) sebacate | 28.20 | 28.78 | 28.83 | -0.63 |
| 506 | Diisobutyl carbonate | 25.31 | 24.82 | 24.67 | 0.64 |
| 507 | Methyl tetradecanoate | 29.40 | 28.85 | 28.76 | 0.64 |
| 508 | Tributyl borate | 24.42 | 23.98 | 23.78 | 0.64 |
| 509 | 2-Chloro-2-methylheptane | 24.97 | 25.62 | 25.61 | -0.64 |
| 510 | 1,7-Heptanediol dinitrate | 40.10 | 41.02 | 40.74 | -0.64 |
| 511 | Dihexyl sulfide | 28.41 | 28.08 | 27.76 | 0.65 |
| 512 | Propyl cyclopropanecarboxylate | 29.00 | 28.39 | 28.35 | 0.65 |
| 513 | Propyl 3-butoxypropanoate | 27.11 | 26.57 | 26.46 | 0.65 |
| 514 | 4-Methyl-4-octanol | 26.10 | 25.50 | 25.45 | 0.65 |
| 515 | Decylbenzene | 30.97 | 31.48 | 31.62 | -0.65 |
| 516 | 1,8-Dichlorooctane | 36.11 | 35.40 | 35.45 | 0.66 |
| 517 | Allyl isothiocyanate | 34.61 | 34.16 | 33.95 | 0.66 |
| 518 | Bis(perfluorohexyl) 3-methylglutarate | 19.90 | 19.34 | 19.24 | 0.66 |
| 519 | Ethyl 1,1-cyclohexanedicarboxylate | 32.99 | 33.65 | 33.65 | -0.66 |
| 520 | 1-Nonanol | 27.51 | 26.96 | 26.85 | 0.66 |
| 521 | 3-Methylcyclopentanone | 29.72 | 30.42 | 30.38 | -0.66 |
| 522 | Ethanethiol | 24.70 | 25.29 | 25.36 | -0.66 |
| 523 | Tridecane | 25.55 | 24.84 | 24.89 | 0.66 |
| 524 | Pentyl 3-Heptyloxypropanoate | 28.32 | 27.57 | 27.66 | 0.66 |
| 525 | Pentyl butoxyacetate | 27.41 | 26.77 | 26.75 | 0.66 |
| 526 | Dipentyl maleate | 30.81 | 31.32 | 31.47 | -0.66 |
| 527 | Ethoxybenzene | 33.46 | 32.73 | 32.80 | 0.66 |
| 528 | Propyl 2-picolinate | 36.98 | 36.58 | 36.31 | 0.67 |
| 529 | Ethyl hexyl carbonate | 27.66 | 27.06 | 26.99 | 0.67 |
| 530 | beta-Ethyl-alpha-methylstyrene | 32.25 | 31.65 | 31.58 | 0.67 |
| 531 | Phenylpropylether | 32.16 | 32.93 | 32.83 | -0.67 |
| 532 | Heptyl 3-Heptyloxypropanoate | 28.77 | 27.97 | 28.10 | 0.67 |
| 533 | N-(3-Hydroxypropyl)pyridinium tris(pentafluoroethyl)trifluorophosphate | 39.98 | 40.67 | 40.65 | -0.67 |
| 534 | 1,6-Hexanediol dinitrate | 41.20 | 40.82 | 40.53 | 0.67 |
| 535 | m-Toluidine | 38.37 | 38.94 | 39.04 | -0.67 |
| 536 | Heptyl 3-methoxypropanoate | 28.86 | 29.47 | 29.53 | -0.67 |
| 537 | 1-Undecyne | 26.59 | 27.12 | 27.27 | -0.68 |
| 538 | Acetonitrile | 28.66 | 27.96 | 27.98 | 0.68 |
| 539 | Heptyl 3-pentyloxypropanoate | 28.34 | 27.57 | 27.66 | 0.68 |
| 540 | 2,2,3,3,4,4,5,5,6,6,7,7-Dodecafluoro-1-heptanol | 23.80 | 24.22 | 24.48 | -0.68 |
| 541 | Methyl pentadecanoate | 29.79 | 29.05 | 29.11 | 0.68 |
| 542 | 4-(1-Propybutyl)pyridine | 31.46 | 32.05 | 32.14 | -0.68 |
| 543 | 4-Chloro-4-methyloctane | 26.47 | 25.82 | 25.79 | 0.68 |
| 544 | n-Butylformate | 25.03 | 25.66 | 25.71 | -0.68 |
| 545 | 1,6-Hexanediol bis(6-H-perfluoroheptanoate) | 25.90 | 26.24 | 26.58 | -0.68 |
| 546 | Dipropyl acetylenedicarboxylate | 32.18 | 32.00 | 31.49 | 0.69 |
| 547 | Propylcyclohexane | 26.33 | 27.00 | 27.02 | -0.69 |
| 548 | Hexyl 3-Hexyloxypropanoate | 28.35 | 27.57 | 27.66 | 0.69 |
| 549 | Dipropyl hexylphosphonate | 28.06 | 28.66 | 28.75 | -0.69 |
| 550 | Dipentyl ethylphosphonate | 27.97 | 28.66 | 28.66 | -0.69 |
| 551 | Butyl perfluorooctanoate | 18.70 | 19.25 | 19.39 | -0.69 |
| 552 | Decamethyltetrasiloxane | 16.20 | 16.70 | 16.89 | -0.69 |
| 553 | Isopentyl butanoate | 24.48 | 23.83 | 23.79 | 0.69 |
| 554 | n-Propyl benzoate | 34.41 | 33.73 | 33.72 | 0.69 |
| 555 | 2,2,3,3,4,4,5,5-Octafluoro-1-pentanol | 24.50 | 25.02 | 25.19 | -0.69 |
| 556 | 1-Ethyl-3-methylimidazolium tetrafluoroborate | 44.30 | 44.57 | 44.99 | -0.69 |
| 557 | Perfluorotributylamine | 16.30 | 16.99 |  | -0.69 |
| 558 | Octylbenzene | 30.46 | 31.04 | 31.15 | -0.69 |
| 559 | 4-Chloro-4-methyldecane | 26.93 | 26.22 | 26.23 | 0.70 |
| 560 | Propyl cyclobutanecarboxylate | 29.15 | 28.59 | 28.45 | 0.70 |
| 561 | 2,3,4-Trimethylpentane | 20.24 | 19.38 | 19.54 | 0.70 |
| 562 | Diisopropyl adipate | 29.33 | 28.68 | 28.63 | 0.70 |
| 563 | 3-Butylpyridine | 31.90 | 32.57 | 32.60 | -0.70 |
| 564 | Isobutyl formate | 23.90 | 24.54 | 24.60 | -0.70 |
| 565 | 2-Isopropylnaphthalene | 35.01 | 34.22 | 34.30 | 0.71 |
| 566 | Isopropyl hydrocinnamate | 33.09 | 33.81 | 33.80 | -0.71 |
| 567 | t-Pentylbenzene | 29.02 | 28.32 | 28.31 | 0.71 |
| 568 | 3-(Propylamino)propionitrile | 31.37 | 31.64 | 32.08 | -0.71 |
| 569 | 3-(6-Hydroxyhexyl)-1-methylimidazolium chloride | 50.00 | 49.48 | 50.71 | -0.71 |
| 570 | Trihexyl tetradecyl phosphonium decanoate | 29.00 | 28.15 | 28.29 | 0.71 |
| 571 | cis-2-Octene | 21.91 | 22.74 | 22.62 | -0.71 |
| 572 | 3-(Butylamino)propionitrile | 30.80 | 31.84 | 31.51 | -0.71 |
| 573 | Methyl ethylpropylmalonate | 30.53 | 31.19 | 31.24 | -0.71 |
| 574 | 1-Butyl-3-methylimidazolium trifluoromethylsulfonate | 35.52 | 35.98 | 36.23 | -0.71 |
| 575 | Dibutyl acetylenedicarboxylate | 31.72 | 32.40 | 32.44 | -0.72 |
| 576 | Cyclopentyl ethyl ether | 26.12 | 26.84 | 26.84 | -0.72 |
| 577 | 3,3-Dimethylpentane | 19.60 | 20.26 | 20.32 | -0.72 |
| 578 | 3-Ethyl-3-hexanol | 25.99 | 25.30 | 25.27 | 0.72 |
| 579 | Propyl 1-naphthaleneacetate | 38.88 | 39.25 | 39.60 | -0.72 |
| 580 | Perfluoro(butylcyclohexane) | 17.70 | 18.39 | 18.42 | -0.72 |
| 581 | Butanoic acid | 26.51 | 25.78 | 25.78 | 0.73 |
| 582 | 1-Decyl-1,8-diazabicyclo(5,4,0)undec-7-enium dicyanamide | 30.65 | 31.21 | 31.38 | -0.73 |
| 583 | Propyl 3-Heptyloxypropanoate | 27.87 | 27.17 | 27.14 | 0.73 |
| 584 | Propyl propanoate | 23.68 | 24.35 | 24.41 | -0.73 |
| 585 | 1-Propanethiol | 24.84 | 25.49 | 25.57 | -0.73 |
| 586 | Propyl isobutanoate | 22.78 | 23.43 | 23.51 | -0.73 |
| 587 | Methyl cyclobutane-1,1-dicarboxylate | 35.11 | 35.75 | 35.84 | -0.73 |
| 588 | 4-(2,2,6-Trimethyl-2-cyclohexenyl)-2-butanol acetate | 29.59 | 28.88 | 28.85 | 0.74 |
| 589 | Dihexyl phosphite | 28.73 | 29.38 | 29.47 | -0.74 |
| 590 | Heptyl 3-propoxypropanoate | 27.88 | 27.17 | 27.14 | 0.74 |
| 591 | Ethyl citraconate | 32.15 | 31.42 | 31.41 | 0.74 |
| 592 | Tris(2-butoxyethyl) phosphite | 29.33 | 30.06 | 30.07 | -0.74 |
| 593 | 5-Decanone | 26.63 | 27.38 | 27.37 | -0.74 |
| 594 | Methyl 3-Hexyloxypropanoate | 28.54 | 29.27 | 29.28 | -0.74 |
| 595 | 4-Dodecanol | 27.83 | 27.24 | 27.08 | 0.75 |
| 596 | 4-Heptanone O-propyloxime | 24.96 | 24.37 | 24.21 | 0.75 |
| 597 | 2,5-Dimethylhexane | 19.86 | 20.50 | 20.61 | -0.75 |
| 598 | Heptyl 3-Hexyloxypropanoate | 28.41 | 27.77 | 27.66 | 0.75 |
| 599 | 1,4-Difluorobenzene | 27.00 | 27.58 | 27.75 | -0.75 |
| 600 | Propanoic acid | 26.20 | 25.58 | 25.45 | 0.75 |
| 601 | 2,4-Xylidine | 37.35 | 37.90 | 38.10 | -0.75 |
| 602 | Triethanolamine | 45.24 | 45.71 | 45.99 | -0.75 |
| 603 | 1-Octyl-1,8-diazabicyclo(5,4,0)undec-7-enium dicyanamide | 31.61 | 30.81 | 30.85 | 0.76 |
| 604 | 2-Phenyl-3-methyl-2-butene | 32.04 | 31.32 | 31.28 | 0.76 |
| 605 | Ethyl 3-Hexyloxypropanoate | 27.63 | 26.77 | 26.87 | 0.76 |
| 606 | 2-(2-Dodecyloxyethoxy)ethanol | 30.60 | 31.20 | 31.36 | -0.76 |
| 607 | 1,4-Bis(butanoyloxy)-2-butyne | 33.15 | 32.40 | 32.39 | 0.76 |
| 608 | 3-Methylmethylenecyclohexane | 24.70 | 25.30 | 25.46 | -0.76 |
| 609 | Allyl alcohol | 25.73 | 26.41 | 26.49 | -0.76 |
| 610 | Ethyl 3-butoxypropanoate | 27.23 | 26.37 | 26.46 | 0.77 |
| 611 | 1,4,7,10-Tetraoxahexadecane | 31.00 | 31.82 | 31.77 | -0.77 |
| 612 | Dipentyl sulfite | 28.92 | 29.49 | 29.69 | -0.77 |
| 613 | 4-Methyl-1-methylenecyclohexane | 24.72 | 25.30 | 25.49 | -0.77 |
| 614 | Butyl 3-Hexyloxypropanoate | 27.84 | 27.17 | 27.06 | 0.78 |
| 615 | Dibutyl oxalate | 29.63 | 28.92 | 28.85 | 0.78 |
| 616 | 4-Heptanone | 25.99 | 26.78 | 26.77 | -0.78 |
| 617 | 2-Butylthiophene | 29.75 | 30.55 | 30.53 | -0.78 |
| 618 | 2-Nonanone | 26.39 | 27.18 | 27.17 | -0.78 |
| 619 | Methyl 3-pentyloxypropanoate | 28.37 | 29.07 | 29.15 | -0.78 |
| 620 | Butyl phenylacetate | 33.33 | 34.13 | 34.12 | -0.79 |
| 621 | Methyl hexadecanoate | 29.95 | 29.25 | 29.16 | 0.79 |
| 622 | Tetrapropyl silicate | 23.58 | 23.18 | 22.79 | 0.79 |
| 623 | 5-Butyl-5-decanol | 27.29 | 26.50 | 26.49 | 0.80 |
| 624 | n-Propylbromoacetate | 31.96 | 32.84 | 32.76 | -0.80 |
| 625 | Bromotrimethylsilane | 20.38 | 19.58 |  | 0.80 |
| 626 | Ethyl cyclopropanecarboxylate | 29.08 | 28.19 | 28.28 | 0.80 |
| 627 | Di-n-butylamine | 24.60 | 23.86 | 23.80 | 0.80 |
| 628 | 2,2,4,4-Tetramethylpentane | 19.91 | 19.04 | 19.11 | 0.80 |
| 629 | Diethyleneglycol-di-n-butylether | 27.00 | 26.04 | 26.20 | 0.80 |
| 630 | Isocumene | 28.98 | 29.94 | 29.78 | -0.80 |
| 631 | 1-Octyl-3-methylimidazolium bis(trifluoromethanesulfonyl) amide | 31.93 | 32.94 | 32.73 | -0.80 |
| 632 | Dihexyl oxalate | 30.45 | 29.72 | 29.64 | 0.81 |
| 633 | 1,7-Dichloroheptane | 36.03 | 35.20 | 35.22 | 0.81 |
| 634 | trans-Decalin | 30.50 | 31.26 | 31.31 | -0.81 |
| 635 | Butyl hydrocinnamate | 33.49 | 34.33 | 34.30 | -0.81 |
| 636 | Propyl 9-decynoate | 31.74 | 31.07 | 30.92 | 0.82 |
| 637 | Acetone oxime | 29.26 | 28.60 | 28.44 | 0.82 |
| 638 | 4-Octanone | 26.15 | 26.98 | 26.97 | -0.82 |
| 639 | Dimethyl azelate | 35.32 | 34.52 | 34.50 | 0.82 |
| 640 | 3-Hexanone O-propyloxime | 25.00 | 24.17 | 24.18 | 0.82 |
| 641 | 2-Pentanone O-propyloxime | 24.63 | 23.97 | 23.81 | 0.82 |
| 642 | Benzyl alcohol | 35.97 | 35.14 | 35.15 | 0.82 |
| 643 | 3-Pentanone O-propyloxime | 24.73 | 23.97 | 23.90 | 0.83 |
| 644 | Methyl methacrylate | 28.00 | 27.17 | 27.17 | 0.83 |
| 645 | 2-t-Butylnaphthalene | 34.18 | 33.50 | 33.35 | 0.83 |
| 646 | 3-Ethylpentane | 20.46 | 21.20 | 21.29 | -0.83 |
| 647 | Chloroacetic acid ethylester | 31.83 | 31.16 | 31.00 | 0.83 |
| 648 | 1,1,2,2-Tetrachloroethane | 35.58 | 35.04 | 34.75 | 0.83 |
| 649 | 2,3-Butyleneglycol | 34.06 | 34.36 | 34.89 | -0.83 |
| 650 | Perfluoroheptane | 13.19 | 12.38 | 12.36 | 0.83 |
| 651 | 3-Chloro-3-methyloctane | 26.69 | 25.82 | 25.86 | 0.83 |
| 652 | 1,4-Bis(valeroyloxy)-2-butyne | 32.03 | 32.80 | 32.87 | -0.84 |
| 653 | Chlorocyclopentane | 30.40 | 29.82 | 29.56 | 0.84 |
| 654 | 3,12-Dichloro-3,12-diethyltetradecane | 33.33 | 32.26 | 32.49 | 0.84 |
| 655 | n-Hexylacetate | 25.53 | 24.75 | 24.69 | 0.84 |
| 656 | 3-Ethyl-2-methylhexane | 21.87 | 20.92 | 21.03 | 0.84 |
| 657 | 3,6-Dichloro-3,6-diethyloctane | 31.80 | 31.06 | 30.96 | 0.84 |
| 658 | n-Propyl cinnamate | 36.59 | 35.73 | 35.75 | 0.84 |
| 659 | 2-Hexylthiophene | 30.08 | 30.95 | 30.92 | -0.84 |
| 660 | 2-Chloroaniline | 43.66 | 42.92 | 42.81 | 0.85 |
| 661 | 2,2,3-Trimethylhexane | 20.97 | 19.98 | 20.12 | 0.85 |
| 662 | 4-Methyl-1-pentanol | 24.44 | 25.24 | 25.29 | -0.85 |
| 663 | Methyl octanoate | 26.92 | 27.65 | 27.77 | -0.85 |
| 664 | 2-Pentanoylthiophene | 35.65 | 36.75 | 36.50 | -0.85 |
| 665 | 5-Chloro-5-propyldecane | 27.44 | 26.62 | 26.59 | 0.85 |
| 666 | Propyl mesaconate | 30.91 | 31.62 | 31.76 | -0.85 |
| 667 | 2-Propyn-1-hexanoate | 29.46 | 30.27 | 30.31 | -0.85 |
| 668 | Heptyl 3-butoxypropanoate | 28.17 | 27.37 | 27.31 | 0.86 |
| 669 | Ethyl 3-methoxypropanoate | 29.26 | 28.47 | 28.40 | 0.86 |
| 670 | 3-Pentylpyridine | 31.61 | 32.77 | 32.47 | -0.86 |
| 671 | Isooctane | 18.77 | 19.56 | 19.63 | -0.86 |
| 672 | 2-n-Propylthiophene | 29.68 | 30.35 | 30.54 | -0.86 |
| 673 | 5-Ethyl-5-decanol | 27.02 | 26.10 | 26.16 | 0.86 |
| 674 | Pentyl 3-Hexyloxypropanoate | 28.20 | 27.37 | 27.34 | 0.86 |
| 675 | 1-Butyl-3-methylimidazolium tricyanomethanide | 47.04 | 46.18 |  | 0.86 |
| 676 | Propyl citraconate | 31.08 | 31.62 | 31.94 | -0.86 |
| 677 | Dimethyl sulfite | 33.97 | 33.29 | 33.10 | 0.87 |
| 678 | 1,1,1,3,3,3-Hexachloro-2,2-difluoropropane | 32.60 | 33.14 | 33.47 | -0.87 |
| 679 | Ethanesulfonic acid | 44.09 | 44.97 |  | -0.88 |
| 680 | Methyl cellosolve | 30.84 | 29.88 | 29.96 | 0.88 |
| 681 | Diisopentyl oxalate | 28.09 | 27.08 | 27.21 | 0.88 |
| 682 | 3-Chloro-3-methylnonane | 26.83 | 26.02 | 25.95 | 0.88 |
| 683 | Propyl trichloroacetate | 30.43 | 31.03 | 31.31 | -0.88 |
| 684 | Methyl 1,1-cyclopentanedicarboxylate | 35.21 | 35.95 | 36.09 | -0.88 |
| 685 | 3-Methyl-3-nonanol | 26.55 | 25.70 | 25.65 | 0.90 |
| 686 | Butyl 2-picolinate | 35.86 | 36.78 | 36.76 | -0.90 |
| 687 | trans-2-Methylcyclohexanol | 30.91 | 30.08 | 30.01 | 0.90 |
| 688 | Ethyl 3-pentyloxypropanoate | 27.36 | 26.57 | 26.46 | 0.90 |
| 689 | Isobutyl butanoate | 22.78 | 23.63 | 23.68 | -0.90 |
| 690 | Octadecane | 27.87 | 26.94 | 26.97 | 0.90 |
| 691 | Isopentylbenzene | 28.34 | 29.26 | 29.24 | -0.90 |
| 692 | Methyl 1,1-cyclopropanedicarboxylate | 36.41 | 35.55 | 35.51 | 0.90 |
| 693 | Diethyl chlorofumarate | 33.20 | 32.45 | 32.30 | 0.90 |
| 694 | Methyl octadecanoate | 30.65 | 29.65 | 29.74 | 0.91 |
| 695 | Ethyl 2-cyano-3-ethylhexanoate | 30.47 | 31.29 | 31.38 | -0.91 |
| 696 | 1-Chlorododecane | 29.87 | 28.99 | 28.96 | 0.91 |
| 697 | Cetane | 27.05 | 26.10 | 26.14 | 0.91 |
| 698 | 2-Nitrobutane | 29.53 | 30.08 | 30.44 | -0.91 |
| 699 | Butyl cyanoacetate | 32.00 | 32.73 | 32.91 | -0.91 |
| 700 | 5-Methyl-5-nonanol | 26.70 | 25.70 | 25.78 | 0.92 |
| 701 | Eicosane | 28.56 | 27.78 | 27.64 | 0.92 |
| 702 | 1,6-Dichlorohexane | 35.99 | 35.00 | 35.07 | 0.92 |
| 703 | trans-1,3-Dimethylcyclohexane | 24.67 | 25.46 | 25.59 | -0.92 |
| 704 | Hexyl 3-Heptyloxypropanoate | 28.71 | 27.77 | 27.79 | 0.92 |
| 705 | Trichloroethylene | 28.80 | 27.88 |  | 0.92 |
| 706 | (2-Bromoethyl)benzene | 38.92 | 38.25 | 37.99 | 0.93 |
| 707 | 1-Nonyl-3-methylimidazolium bis(trifluoromethanesulfonyl) amide | 32.00 | 33.14 | 32.93 | -0.93 |
| 708 | Diisopentyl sulfide | 25.97 | 25.44 | 25.04 | 0.93 |
| 709 | Ethyl lactate | 28.90 | 29.61 | 29.83 | -0.93 |
| 710 | Triisobutyl phosphate | 26.40 | 26.04 | 27.33 | -0.93 |
| 711 | Heptyl bromide | 28.56 | 29.47 | 29.49 | -0.93 |
| 712 | Methyl hydrocinnamate | 37.30 | 36.43 | 36.37 | 0.93 |
| 713 | Methyl 3-ethyl-3-methylglutarate | 32.04 | 31.19 | 31.10 | 0.94 |
| 714 | Perfluorohexane | 11.91 | 12.78 | 12.85 | -0.94 |
| 715 | 3-Methyl-1-butanethiol | 25.60 | 24.77 | 24.66 | 0.94 |
| 716 | trans-1,2-Dichloroethylene | 25.00 | 25.94 |  | -0.94 |
| 717 | Acetone O-ethyloxime | 22.48 | 23.37 | 23.42 | -0.94 |
| 718 | 9-H-Perfluorononyl 2-ethylhexanoate | 23.10 | 21.96 | 22.15 | 0.95 |
| 719 | Methyl 3-butynoate | 32.94 | 32.17 | 31.99 | 0.95 |
| 720 | N-Nitrosodipropylamine | 31.64 | 31.03 | 30.69 | 0.95 |
| 721 | Methylbenzene | 28.52 | 29.50 | 29.47 | -0.95 |
| 722 | Nopinene | 26.82 | 27.52 | 27.77 | -0.95 |
| 723 | 1-Bromooctane | 28.89 | 29.67 | 29.84 | -0.95 |
| 724 | Propyl allylmalonate | 29.29 | 30.08 | 30.24 | -0.95 |
| 725 | 4-Chloro-4-methylnonane | 26.99 | 26.02 | 26.03 | 0.96 |
| 726 | 6-Chloro-6-methyldodecane | 27.46 | 26.62 | 26.50 | 0.96 |
| 727 | Tetradecane | 26.14 | 25.26 | 25.18 | 0.96 |
| 728 | Bis(5-H-perfluoropentyl) glutarate | 27.50 | 27.78 | 28.47 | -0.97 |
| 729 | Allyl butyrate | 26.13 | 25.20 | 25.16 | 0.97 |
| 730 | Hexyl 3-ethoxypropanoate | 27.63 | 26.77 | 26.66 | 0.97 |
| 731 | Di-n-propylsulfate | 32.03 | 32.76 | 33.00 | -0.97 |
| 732 | Dimethylketazine | 26.67 | 25.86 | 25.70 | 0.97 |
| 733 | Propyl thiocyanate | 32.74 | 33.46 | 33.71 | -0.97 |
| 734 | N,N-Dibutylaniline | 32.30 | 32.82 | 33.27 | -0.97 |
| 735 | Ethyl 4-pyridinecarboxylate | 37.15 | 36.14 | 36.17 | 0.98 |
| 736 | 4-Octanol | 25.43 | 26.44 | 26.41 | -0.98 |
| 737 | Ethylcyclohexane | 25.57 | 26.58 | 26.55 | -0.98 |
| 738 | Diisopentyl sulfite | 27.86 | 27.25 | 26.88 | 0.98 |
| 739 | N-Butyl-2-methylpyridinium bis(trifluoromethanesulfonyl) amide | 36.50 | 37.48 |  | -0.98 |
| 740 | 1,9-Dibromononane | 39.55 | 38.56 | 38.56 | 0.99 |
| 741 | Propyl 3-ethoxypropanoate | 27.25 | 26.17 | 26.26 | 0.99 |
| 742 | 3-Chloro-3-methylpentane | 24.23 | 25.22 | 25.22 | -0.99 |
| 743 | Bis(2-ethylhexyl) adipate | 30.20 | 29.08 | 29.21 | 0.99 |
| 744 | o-Toluidine | 40.03 | 38.94 | 39.04 | 0.99 |
| 745 | Butyl 1-naphthoate | 38.18 | 39.25 | 39.17 | -0.99 |
| 746 | Octylcyanide | 28.50 | 29.36 | 29.49 | -0.99 |
| 747 | Butyl 3-butenoate | 26.58 | 25.40 | 25.58 | 1.00 |
| 748 | 1-Heptanethiol | 27.26 | 26.29 | 26.26 | 1.00 |
| 749 | Dimethyl butyl isopropylammonium bis(trifluoromethylsulfonyl)amide | 32.80 | 31.97 | 31.80 | 1.00 |
| 750 | Isobutylyl cyanoacetate | 30.66 | 31.61 | 31.66 | -1.00 |
| 751 | Butylbenzene | 29.23 | 30.16 | 30.23 | -1.00 |
| 752 | 1-Chlorotetradecane | 30.36 | 29.39 | 29.36 | 1.00 |
| 753 | Nitroisobutane | 28.37 | 29.17 | 29.37 | -1.00 |
| 754 | Octane | 21.78 | 22.74 | 22.78 | -1.00 |
| 755 | Caprylonitrile | 28.10 | 29.16 | 29.10 | -1.00 |
| 756 | Dioctyl ethylphosphonate | 28.99 | 29.86 | 29.99 | -1.00 |
| 757 | Diheptyl sulfide | 29.32 | 28.48 | 28.31 | 1.01 |
| 758 | 1,6-Hexamethylene glycol bis(2-ethylhexanoate) | 30.20 | 29.08 | 29.19 | 1.01 |
| 759 | Perfluoro-1,4-dimethylcyclohexane | 16.30 | 17.24 | 17.31 | -1.01 |
| 760 | 2-Chlorooctane | 26.40 | 25.46 | 25.39 | 1.01 |
| 761 | Ethyl 4-methyl-1,1-cyclohexanedicarboxylate | 31.74 | 32.73 | 32.75 | -1.01 |
| 762 | (Chloromethyl)benzene | 37.50 | 36.57 | 36.48 | 1.02 |
| 763 | Dipropyl cyclopropane-1,1-dicarboxylate | 30.99 | 32.02 | 32.01 | -1.02 |
| 764 | cis-3-Methylcyclohexanol | 29.20 | 30.08 | 30.22 | -1.02 |
| 765 | 1-Chlorohexadecane | 30.98 | 29.79 | 29.96 | 1.02 |
| 766 | 2-Methyl-2-hexanol | 24.18 | 25.10 | 25.20 | -1.02 |
| 767 | Methyl alpha-bromo-cis-cinnamate | 43.48 | 44.50 |  | -1.02 |
| 768 | 1,5-Pentanediol bis(trifluoroacetate) | 24.40 | 23.32 | 23.38 | 1.02 |
| 769 | 1-Ethyl-3-methylimidazolium acetate | 38.10 | 37.63 | 37.08 | 1.02 |
| 770 | 4,13-Dichloro-4,13-dipropylhexadecane | 32.00 | 33.06 | 33.02 | -1.02 |
| 771 | Methyl 10-undecenoate | 29.95 | 28.90 | 28.92 | 1.03 |
| 772 | Hexanenitrile | 27.83 | 28.76 | 28.86 | -1.03 |
| 773 | 2-Heptylthiophene | 30.40 | 31.15 | 31.43 | -1.03 |
| 774 | 2,3,3-Trimethylpentane | 20.66 | 19.56 | 19.63 | 1.03 |
| 775 | n-Octanoic acid | 27.50 | 26.58 | 26.47 | 1.03 |
| 776 | 2-Methylpropanoic acid | 25.55 | 24.66 | 24.51 | 1.04 |
| 777 | Dipropyl phosphite | 29.25 | 28.18 | 28.21 | 1.04 |
| 778 | Hexyl glycolate | 29.66 | 30.53 | 30.70 | -1.04 |
| 779 | Methyl t-butyl sulfide | 23.48 | 24.52 |  | -1.04 |
| 780 | Propanone | 25.14 | 25.98 | 26.18 | -1.04 |
| 781 | Anethole | 35.14 | 36.19 | 36.18 | -1.04 |
| 782 | 1-Chloroheptane | 27.02 | 27.99 | 28.06 | -1.04 |
| 783 | 1-Chlorooctane | 27.15 | 28.19 | 28.20 | -1.05 |
| 784 | Ethyl dichloroacetate | 32.57 | 31.58 | 31.52 | 1.05 |
| 785 | 5-Chloro-5-methylundecane | 27.50 | 26.42 | 26.45 | 1.05 |
| 786 | Butyronitrile | 27.44 | 28.36 | 28.49 | -1.05 |
| 787 | 1-Butyl-1-methylpyrrolidinium acetate | 37.40 | 38.41 | 38.45 | -1.05 |
| 788 | Tripropyl borate | 22.48 | 23.38 | 23.54 | -1.06 |
| 789 | 3-Chloro-3-ethylpentane | 26.36 | 25.42 | 25.30 | 1.06 |
| 790 | Propyl butoxyacetate | 27.40 | 26.37 | 26.34 | 1.06 |
| 791 | Diethylketone | 25.26 | 26.38 | 26.32 | -1.06 |
| 792 | Methyl benzoate | 37.17 | 36.03 | 36.11 | 1.06 |
| 793 | Isopropylcyclopentane | 24.38 | 25.46 | 25.44 | -1.06 |
| 794 | Amylbenzene | 29.45 | 30.38 | 30.52 | -1.07 |
| 795 | 2,2-Dimethylhexane | 19.61 | 20.68 | 20.68 | -1.07 |
| 796 | 3,4-Dimethylheptane | 21.91 | 20.92 | 20.84 | 1.07 |
| 797 | 1,5-Dichloropentane | 35.75 | 34.80 | 34.68 | 1.07 |
| 798 | 1-Decyl-3-methylimidazolium bis(trifluoromethanesulfonyl) amide | 32.20 | 33.34 | 33.27 | -1.07 |
| 799 | 3,11-Dichloro-3,11-diethyltridecane | 33.05 | 32.06 | 31.97 | 1.08 |
| 800 | 5-Chloro-5-butyldecane | 27.90 | 26.82 | 26.82 | 1.08 |
| 801 | Dipropoxymethane | 23.26 | 24.16 | 24.34 | -1.08 |
| 802 | Dipropyl ethylmalonate | 28.99 | 28.00 | 27.91 | 1.08 |
| 803 | trans-2-Decalone | 36.12 | 35.06 | 35.04 | 1.08 |
| 804 | cis-1,4-Dimethylcyclohexane | 24.43 | 25.46 | 25.52 | -1.09 |
| 805 | cis-Decalin | 32.18 | 31.26 | 31.09 | 1.09 |
| 806 | Ethyl 3-methyl-1,1-cyclohexanedicarboxylate | 31.73 | 32.73 | 32.82 | -1.09 |
| 807 | Butylcyclopentane | 25.87 | 27.00 | 26.96 | -1.09 |
| 808 | trans-Hexahydro-2-methyleneindan | 28.83 | 29.58 | 29.92 | -1.09 |
| 809 | 4-Heptylpyridine | 32.18 | 33.17 | 33.27 | -1.09 |
| 810 | 3-Methyl-3-octanol | 26.52 | 25.50 | 25.42 | 1.10 |
| 811 | Cyclopentyl formate | 31.79 | 30.50 | 30.69 | 1.10 |
| 812 | Linalool | 26.62 | 27.62 | 27.72 | -1.10 |
| 813 | Dipentyloxymethane | 25.97 | 24.96 | 24.87 | 1.10 |
| 814 | Ethyl isobutyrate | 22.19 | 23.23 | 23.29 | -1.10 |
| 815 | Tripropyl phosphite | 25.06 | 24.00 | 23.96 | 1.10 |
| 816 | 3-Hexanone | 25.58 | 26.58 | 26.68 | -1.10 |
| 817 | Propyl isonicotinate | 35.22 | 36.34 | 36.32 | -1.10 |
| 818 | Diheptyl ethylphosphonate | 28.47 | 29.46 | 29.57 | -1.10 |
| 819 | 1-o-tolyl-1-p-tolylethane | 35.50 | 36.46 | 36.60 | -1.10 |
| 820 | 2-Ethyl-1-butanol | 24.32 | 25.24 | 25.42 | -1.10 |
| 821 | 4-Heptanone ketazine | 26.57 | 27.46 | 27.67 | -1.10 |
| 822 | Dihexyl ethylphosphonate | 28.07 | 29.06 | 29.17 | -1.10 |
| 823 | 3,5-Dimethylisoxazole | 32.89 | 33.99 |  | -1.10 |
| 824 | 2-Bromotoluene | 34.63 | 35.44 | 35.73 | -1.10 |
| 825 | Dimethyl thiosulfate | 32.76 | 33.87 |  | -1.11 |
| 826 | Hexamethyldisiloxane | 15.48 | 16.06 | 16.59 | -1.11 |
| 827 | (Isopropylthio)benzene | 33.46 | 34.20 | 34.57 | -1.11 |
| 828 | Perfluoro-2-methylbutane | 10.48 | 11.23 | 11.60 | -1.12 |
| 829 | N-Methylaniline | 37.38 | 38.02 | 38.50 | -1.12 |
| 830 | Ethyl 2-cyano-3-methylhexanoate | 30.11 | 31.09 | 31.23 | -1.12 |
| 831 | Nonadecane | 28.59 | 27.36 | 27.47 | 1.12 |
| 832 | Isobutylcyclopentane | 24.73 | 25.88 | 25.85 | -1.12 |
| 833 | Dimethyl methylmalonate | 33.51 | 32.40 | 32.38 | 1.13 |
| 834 | Diethyl propylmalonate | 28.93 | 27.80 | 27.80 | 1.13 |
| 835 | Ethyl hydrocinnamate | 35.02 | 33.93 | 33.89 | 1.13 |
| 836 | Tris(2-pentoxyethyl) phosphite | 29.62 | 30.66 | 30.75 | -1.13 |
| 837 | Methyl alpha-bromocinnamate | 45.63 | 44.50 |  | 1.13 |
| 838 | 1,2,3-Tribromopropane | 44.82 | 43.53 | 43.69 | 1.13 |
| 839 | 1-Decyne | 25.93 | 26.90 | 27.06 | -1.13 |
| 840 | Pentanoic acid | 27.13 | 25.98 | 25.99 | 1.14 |
| 841 | Di-n-pentyl adipate | 31.25 | 30.12 | 30.11 | 1.14 |
| 842 | 3-(Trifluoromethyl)benzyl perfluorobutyrate | 24.80 | 26.29 | 25.94 | -1.14 |
| 843 | Dimethyl hexyl isopropylammonium bis(trifluoromethylsulfonyl)amide | 31.40 | 32.37 | 32.55 | -1.15 |
| 844 | 2-Octyloxyethanol | 29.80 | 28.58 | 28.65 | 1.15 |
| 845 | t-Butylcyclohexane | 26.66 | 25.36 | 25.51 | 1.15 |
| 846 | 1-Nonene | 23.02 | 22.01 | 21.87 | 1.15 |
| 847 | Cyclohexene | 26.17 | 27.26 | 27.32 | -1.15 |
| 848 | Propyl cyanoacetate | 33.83 | 32.53 | 32.68 | 1.15 |
| 849 | Methyl 3-Heptyloxypropanoate | 28.33 | 29.47 | 29.48 | -1.15 |
| 850 | 1-Ethyl-3-methylimidazolium butyltrifluoroborate | 34.20 | 35.35 |  | -1.15 |
| 851 | Methyl mesaconate | 35.27 | 33.92 | 34.12 | 1.15 |
| 852 | 1-Propyl-3-methylimidazolium acetate | 36.80 | 37.83 | 37.95 | -1.15 |
| 853 | Ethyl cyclobutane-1,1-dicarboxylate | 31.94 | 33.25 | 33.09 | -1.15 |
| 854 | t-Butyl nicotinate | 33.94 | 35.08 | 35.09 | -1.15 |
| 855 | 2-Pentylnaphthalene | 34.58 | 35.78 | 35.73 | -1.15 |
| 856 | 1-Butoxy-1-t-butoxyethane | 22.65 | 21.74 | 21.49 | 1.16 |
| 857 | 1-Propylnaphthalene | 36.70 | 35.34 | 35.54 | 1.16 |
| 858 | 1-Undecyl-3-methylimidazolium bis(trifluoromethanesulfonyl) amide | 32.30 | 33.54 | 33.46 | -1.16 |
| 859 | n-Propylvinylacetate | 26.29 | 25.20 | 25.13 | 1.16 |
| 860 | Ethyl cyclohexane-1,1-diacetate | 32.98 | 34.05 | 34.15 | -1.17 |
| 861 | n-Propyliodoacetate | 34.44 | 35.46 | 35.61 | -1.17 |
| 862 | 2-Methylthiophene | 30.83 | 29.95 | 29.66 | 1.17 |
| 863 | Propyl dichloroacetate | 30.74 | 31.78 | 31.91 | -1.17 |
| 864 | Tributylamine | 24.39 | 23.05 | 23.22 | 1.17 |
| 865 | Pinacolone | 23.43 | 24.52 | 24.60 | -1.17 |
| 866 | Tris(2-hexoxyethyl) phosphite | 29.91 | 31.26 | 31.08 | -1.17 |
| 867 | Ethyl 2-cyano-3-propylcrotonate | 33.21 | 34.27 | 34.38 | -1.17 |
| 868 | 1-Hexylnaphthalene | 35.06 | 36.00 | 36.23 | -1.17 |
| 869 | Phenyltrimethylsilane | 24.69 | 23.51 |  | 1.18 |
| 870 | Pentyl 3-butenoate | 26.78 | 25.60 | 25.60 | 1.18 |
| 871 | 2-Nitrotoluene | 41.46 | 40.08 | 40.28 | 1.18 |
| 872 | 2-Propyn-1-heptanoate | 29.68 | 30.47 | 30.86 | -1.18 |
| 873 | n-Heptaldehyde | 26.84 | 25.80 | 25.66 | 1.18 |
| 874 | Benzyl ethyl ether | 32.18 | 31.18 | 31.00 | 1.18 |
| 875 | Isohexylamine | 23.39 | 24.60 | 24.57 | -1.18 |
| 876 | Diheptyl oxalate | 31.29 | 30.12 | 30.11 | 1.18 |
| 877 | Ethyl 3,3-dimethylglutarate | 29.65 | 28.49 | 28.47 | 1.18 |
| 878 | 2-Bromobutane | 25.01 | 26.35 | 26.19 | -1.18 |
| 879 | Cyclohexylamine | 31.22 | 29.43 | 30.03 | 1.19 |
| 880 | n-Propylformate | 24.53 | 25.46 | 25.72 | -1.19 |
| 881 | Ethyl 3-propoxypropanoate | 27.33 | 26.17 | 26.14 | 1.19 |
| 882 | 1,9-Dichlorononane | 36.51 | 35.60 | 35.32 | 1.19 |
| 883 | 4-Fluoronitrobenzene | 38.70 | 39.71 | 39.89 | -1.19 |
| 884 | 3-Methyl-3-pentanol | 23.71 | 24.90 | 24.90 | -1.19 |
| 885 | Bis(2-ethylhexyl) sebacate | 31.10 | 29.88 | 29.90 | 1.20 |
| 886 | Diisopropyl sulfide | 23.18 | 24.08 | 24.38 | -1.20 |
| 887 | Dipropyl methylmalonate | 29.11 | 27.80 | 27.91 | 1.20 |
| 888 | Pentanenitrile | 27.44 | 28.56 | 28.64 | -1.20 |
| 889 | n-Butylchloroacetate | 30.22 | 31.56 | 31.42 | -1.20 |
| 890 | 1,6-Diiodohexane | 44.49 | 43.20 | 43.28 | 1.21 |
| 891 | N-Hexylpyridinium bis(trifluoromethanesulfonyl) amide | 34.20 | 35.04 | 35.41 | -1.21 |
| 892 | Diethyl ethylphosphonate | 28.68 | 27.46 | 27.46 | 1.22 |
| 893 | 1-Heptene | 20.30 | 21.57 | 21.52 | -1.22 |
| 894 | 4,5-Dimethyloctane | 22.54 | 21.34 | 21.32 | 1.22 |
| 895 | 1-Phenyl-2-pentanone | 34.91 | 36.16 | 36.13 | -1.22 |
| 896 | s-Butylcyclohexane | 27.46 | 26.30 | 26.23 | 1.23 |
| 897 | 1-Iodohexadecane | 32.73 | 33.89 | 33.96 | -1.23 |
| 898 | Butyl fumarate | 30.71 | 31.95 | 31.94 | -1.23 |
| 899 | 4-Methylvaleronitrile | 26.53 | 27.64 | 27.76 | -1.23 |
| 900 | 3-Pentanol | 24.60 | 25.84 | 25.83 | -1.23 |
| 901 | 4-Isopropylbenzaldehyde | 34.03 | 32.62 | 32.80 | 1.23 |
| 902 | 1-Phenyl-1-propanol | 34.00 | 35.22 | 35.23 | -1.23 |
| 903 | Benzonitrile | 38.79 | 37.54 | 37.55 | 1.24 |
| 904 | Butylcellosolve | 26.55 | 27.78 | 27.79 | -1.24 |
| 905 | Fluorobenzene | 27.26 | 28.99 | 28.50 | -1.24 |
| 906 | 1-Butyl-4-methylpyridinium tetrafluoroborate | 45.40 | 46.43 | 46.64 | -1.24 |
| 907 | 2-Chloro-2-methylhexane | 24.15 | 25.42 | 25.39 | -1.24 |
| 908 | trans-2-Octene | 21.38 | 22.74 | 22.62 | -1.24 |
| 909 | 3-Ethyl-3-methylhexane | 22.30 | 21.10 | 21.06 | 1.24 |
| 910 | 1,3-Dichlorobenzene | 36.01 | 36.28 | 37.26 | -1.25 |
| 911 | 3,4-Diethylhexane | 22.62 | 21.34 | 21.37 | 1.25 |
| 912 | Butyl allylmalonate | 28.98 | 30.28 | 30.23 | -1.25 |
| 913 | Diethyl disulfide | 30.91 | 29.76 | 29.65 | 1.26 |
| 914 | 3,8-Dichloro-3,8-diethyldecane | 32.63 | 31.46 | 31.37 | 1.26 |
| 915 | Ethyl caprylate | 26.47 | 25.15 | 25.21 | 1.26 |
| 916 | 2,5-Lutidine | 32.31 | 31.17 | 31.05 | 1.26 |
| 917 | 1,5-Pentanediol bis(perfluorobutyrate) | 20.60 | 21.72 | 21.86 | -1.26 |
| 918 | Dibutyl succinate | 30.65 | 29.32 | 29.39 | 1.26 |
| 919 | Crotonaldehyde | 25.66 | 26.80 | 26.92 | -1.26 |
| 920 | Butyl bromoacetate | 31.87 | 33.04 | 33.13 | -1.26 |
| 921 | 1-Butylpyridinium nitrate | 51.50 | 51.50 | 50.23 | 1.27 |
| 922 | 1-Hexyl-3-methylimidazolium tris(pentafluoroethyl)trifluorophosphate | 31.81 | 32.99 | 33.08 | -1.27 |
| 923 | Tributyl phosphite | 25.84 | 24.60 | 24.57 | 1.27 |
| 924 | Ethyl 3-methyl-1,1-cyclopentanedicarboxylate | 31.12 | 32.53 | 32.39 | -1.27 |
| 925 | Methyl valerate | 25.76 | 27.05 | 27.03 | -1.27 |
| 926 | Butyl nicotinate | 35.22 | 36.54 | 36.49 | -1.27 |
| 927 | 2-Propylpyridine | 31.36 | 32.61 | 32.63 | -1.27 |
| 928 | Ethyl crotonate | 27.18 | 25.95 | 25.90 | 1.28 |
| 929 | Ethyl 9-decynoate | 31.97 | 30.87 | 30.69 | 1.28 |
| 930 | Ethyl methyl ketone oxime | 29.85 | 28.80 | 28.57 | 1.28 |
| 931 | Triethylamine | 20.72 | 21.85 | 22.00 | -1.28 |
| 932 | Ethyl butoxyacetate | 27.39 | 26.17 | 26.11 | 1.28 |
| 933 | beta-Propylstyrene | 33.23 | 31.98 | 31.95 | 1.28 |
| 934 | Trihexyl tetradecyl phosphonium bromide | 30.90 | 31.82 | 32.19 | -1.29 |
| 935 | Ethyl campholanate | 27.64 | 26.21 | 26.35 | 1.29 |
| 936 | 1-Butyl-3-methylpyridinium tetrafluoroborate | 47.50 | 46.43 | 46.21 | 1.29 |
| 937 | Heptanonitrile | 27.75 | 28.96 | 29.04 | -1.29 |
| 938 | Benzophenone | 44.05 | 45.14 | 45.34 | -1.29 |
| 939 | 1-Decene | 23.54 | 22.23 | 22.24 | 1.30 |
| 940 | Heptyl 3-ethoxypropanoate | 28.26 | 26.97 | 26.96 | 1.30 |
| 941 | 1-Nitrohexane | 29.54 | 30.69 | 30.84 | -1.30 |
| 942 | Methyl cyclohexane-1,1-diacetate | 35.30 | 36.55 | 36.60 | -1.30 |
| 943 | Dibutyl azodicarboxylate | 31.36 | 32.66 |  | -1.30 |
| 944 | Ethyl 3-butenoate | 26.16 | 25.00 | 24.85 | 1.31 |
| 945 | Di-n-pentyl malonate | 28.30 | 29.52 | 29.61 | -1.31 |
| 946 | Diisopropyl phosphonate | 26.40 | 27.54 | 27.71 | -1.31 |
| 947 | Dibutyl acetal | 24.44 | 23.20 | 23.13 | 1.31 |
| 948 | Dimethyl 2-butynedioate | 37.91 | 36.60 | 36.59 | 1.32 |
| 949 | Perfluoro-2-methylpentane | 12.12 | 10.83 | 10.80 | 1.32 |
| 950 | 1-Phenyl-2-butene | 30.48 | 31.76 | 31.80 | -1.32 |
| 951 | Diethyl thiosulfate | 30.27 | 28.95 |  | 1.32 |
| 952 | 4-Hexylpyridine | 31.66 | 32.97 | 32.98 | -1.32 |
| 953 | 1-Butyl-3-methylimidazolium dicyanoamide | 45.81 | 44.32 | 44.48 | 1.33 |
| 954 | Isopentyl alcohol | 23.71 | 25.04 | 25.04 | -1.33 |
| 955 | 1,2-Dimethylbenzene | 29.76 | 28.48 | 28.43 | 1.33 |
| 956 | n-Hexanoic acid | 27.55 | 26.18 | 26.22 | 1.33 |
| 957 | N-Nitrosodibutylamine | 30.36 | 31.43 | 31.69 | -1.33 |
| 958 | n-Butyltrichloroacetate | 30.18 | 31.23 | 31.51 | -1.33 |
| 959 | 4,9-Dichloro-4,9-dipropyldodecane | 31.16 | 32.26 | 32.49 | -1.33 |
| 960 | Dimethyl suberate | 35.65 | 34.32 | 34.31 | 1.34 |
| 961 | 3-Dodecanol | 28.60 | 27.24 | 27.26 | 1.34 |
| 962 | Diisopropylamine | 19.68 | 20.96 | 21.02 | -1.34 |
| 963 | Pentadecane | 27.07 | 25.68 | 25.73 | 1.34 |
| 964 | Hexyl dihydrogen phosphate | 28.05 | 29.16 | 29.39 | -1.34 |
| 965 | Isopropylacetate | 22.30 | 23.83 | 23.64 | -1.34 |
| 966 | Methylcyclohexylether | 28.21 | 29.54 | 29.56 | -1.35 |
| 967 | Dimethyl disulfide | 34.06 | 34.60 | 35.41 | -1.35 |
| 968 | 1-Chlorooctadecane | 31.51 | 30.19 | 30.15 | 1.36 |
| 969 | N-Butylpyridinium bis(trifluoromethanesulfonyl) amide | 33.40 | 34.64 | 34.76 | -1.36 |
| 970 | Acrylic acid | 27.60 | 26.23 | 26.24 | 1.36 |
| 971 | 1-Hexanethiol | 27.50 | 26.09 | 26.14 | 1.36 |
| 972 | 5-Chloro-5-methyldecane | 27.59 | 26.22 | 26.23 | 1.36 |
| 973 | Dibenzylamine | 41.10 | 42.22 | 42.46 | -1.36 |
| 974 | 1-Heptylnaphthalene | 34.59 | 36.22 | 35.95 | -1.36 |
| 975 | 2,2,3,3-Tetramethylbutane | 20.22 | 18.62 | 18.85 | 1.37 |
| 976 | trans-1,2-Dimethylcyclohexane | 24.04 | 25.46 | 25.41 | -1.37 |
| 977 | N-Ethyl-2-methylpyridinium bis(trifluoromethylsulfonyl)amide | 38.45 | 37.08 |  | 1.37 |
| 978 | 1-Octanethiol | 27.77 | 26.49 | 26.39 | 1.38 |
| 979 | 1,2-Diethylbenzene | 30.29 | 28.92 | 28.91 | 1.38 |
| 980 | Trioctyl phosphite | 28.31 | 27.00 | 26.93 | 1.38 |
| 981 | Tetraethoxysilane | 21.67 | 22.38 | 23.05 | -1.38 |
| 982 | Butyl phenyl sulfide | 34.50 | 35.80 | 35.88 | -1.38 |
| 983 | Butyl phenylpropiolate | 36.21 | 37.41 | 37.59 | -1.38 |
| 984 | 3-Methyl-4-(2,2,6-trimethyl-2-cyclohexenyl)-2-butanol acetate | 29.47 | 27.96 | 28.08 | 1.39 |
| 985 | Pyrrolidine | 29.23 | 28.02 | 27.84 | 1.39 |
| 986 | Furfuranol | 38.00 | 36.61 |  | 1.39 |
| 987 | 2-Hexanol | 24.71 | 26.04 | 26.10 | -1.39 |
| 988 | Bis(2-butoxyethyl)phosphite | 30.96 | 32.22 | 32.35 | -1.39 |
| 989 | Trimethyl hexylammonium bis(trifluoromethylsulfonyl)amide | 35.88 | 36.80 | 37.27 | -1.39 |
| 990 | Acetylacetone | 30.90 | 32.38 | 32.30 | -1.40 |
| 991 | 3-Ethyl-4-methylhexane | 22.34 | 20.92 | 20.94 | 1.40 |
| 992 | Ethyl 2-cyano-3-propylhexanoate | 30.10 | 31.49 | 31.50 | -1.40 |
| 993 | Allylamine | 24.92 | 25.77 | 26.32 | -1.40 |
| 994 | Perfluorodibutyl ether | 13.40 | 14.74 | 14.80 | -1.40 |
| 995 | Butyl 2-thiophenecarboxylate | 33.12 | 34.52 | 34.52 | -1.40 |
| 996 | 1-Phenyl-3-hexanone | 34.83 | 36.36 | 36.23 | -1.40 |
| 997 | Allyl propanoate | 26.35 | 25.00 | 24.94 | 1.41 |
| 998 | s-Butylacetate | 22.56 | 24.03 | 23.97 | -1.41 |
| 999 | 3-Methylhexane | 19.79 | 21.20 | 21.20 | -1.41 |
| 1000 | Isobutyl phenylpropiolate | 35.05 | 36.29 | 36.46 | -1.41 |
| 1001 | trans-3-Methylcyclohexanol | 28.80 | 30.08 | 30.22 | -1.42 |
| 1002 | Diethyl sulphide | 25.12 | 26.48 | 26.54 | -1.42 |
| 1003 | Methyl 3,3-dipropylglutarate | 30.49 | 31.79 | 31.91 | -1.42 |
| 1004 | Isopentyl propionate | 25.10 | 23.63 | 23.68 | 1.42 |
| 1005 | Hexyl bromide | 27.88 | 29.27 | 29.30 | -1.42 |
| 1006 | Ethyl salicylate | 38.82 | 37.29 | 37.40 | 1.42 |
| 1007 | Cyclogeraniolane | 23.50 | 24.94 | 24.93 | -1.43 |
| 1008 | Diisobutyl succinate | 28.45 | 27.08 | 27.02 | 1.43 |
| 1009 | 1,1-Diethoxyethane | 21.40 | 22.40 | 22.83 | -1.43 |
| 1010 | Heptyl glycolate | 29.40 | 30.73 | 30.83 | -1.43 |
| 1011 | n-Butylethylether | 20.65 | 22.00 | 22.08 | -1.43 |
| 1012 | 1-Nitropentane | 29.31 | 30.49 | 30.74 | -1.43 |
| 1013 | trans-2-Methylenedecalin | 30.84 | 29.80 | 29.40 | 1.44 |
| 1014 | Ethyl-t-butylsulfide | 23.54 | 22.10 |  | 1.44 |
| 1015 | s-Butyl vinyl ether | 20.76 | 21.88 | 22.20 | -1.44 |
| 1016 | Hemimellitene | 28.83 | 27.46 | 27.39 | 1.44 |
| 1017 | Butyl vinyl ether | 21.99 | 22.20 | 23.43 | -1.44 |
| 1018 | 1,1-Dimethylcyclohexane | 24.13 | 25.64 | 25.57 | -1.44 |
| 1019 | Piperidine | 29.48 | 28.22 | 28.04 | 1.44 |
| 1020 | 2-Tolunitrile | 37.86 | 36.50 | 36.41 | 1.45 |
| 1021 | Dipentyl acetylenedicarboxylate | 31.42 | 32.80 | 32.87 | -1.45 |
| 1022 | 2-Methylcyclohexanone | 32.01 | 30.62 | 30.56 | 1.45 |
| 1023 | Octadecyl perfluorobutyrate | 25.10 | 23.65 | 23.65 | 1.45 |
| 1024 | 1-Ethylimidazolium bis(trifluoromethanesulfonyl) amide | 34.94 | 33.49 |  | 1.45 |
| 1025 | Chlorocyclohexane | 31.09 | 30.02 | 29.64 | 1.45 |
| 1026 | 1-Dodecyl-3-methylimidazolium bis(trifluoromethanesulfonyl) amide | 32.30 | 33.74 | 33.75 | -1.45 |
| 1027 | 3-Methyl-4-(2,2,6-trimethyl-2-cyclohexenyl)-2-butanol | 30.63 | 29.57 | 29.18 | 1.45 |
| 1028 | Methyl cyclopentane-1,1-diacetate | 34.85 | 36.35 | 36.30 | -1.45 |
| 1029 | 2,2-Dimethyl-3-ethylpentane | 21.45 | 19.98 | 19.99 | 1.46 |
| 1030 | 4,7-Dichloro-4,7-dipropyldecane | 30.31 | 31.86 | 31.77 | -1.46 |
| 1031 | Diheptyl phosphite | 28.72 | 29.78 | 30.18 | -1.46 |
| 1032 | 1-(2-(2-Methoxyethoxy)ethyl)-3-methylimidazolium bis(trifluoromethylsulfonyl)amide | 36.50 | 37.88 | 37.96 | -1.46 |
| 1033 | 1-Butylamine | 24.00 | 25.32 | 25.46 | -1.46 |
| 1034 | 3,4-Dichlorotoluene | 36.50 | 35.24 | 35.04 | 1.46 |
| 1035 | Bis(9-H-perfluorononyl) 3-methylglutarate | 25.00 | 23.66 | 23.54 | 1.46 |
| 1036 | 1-Butyl-1-methylpiperidinium bis(trifluoromethylsulfonyl)amide | 34.20 | 32.72 | 32.73 | 1.47 |
| 1037 | 1-Butyl-3-methylimidazolium bis(trifluoromethanesulfonyl) amide | 33.60 | 32.14 | 32.13 | 1.47 |
| 1038 | Dimethyl pimelate | 35.57 | 34.12 | 34.10 | 1.47 |
| 1039 | 1,1-Diisobutoxyethane | 22.40 | 20.96 | 20.93 | 1.47 |
| 1040 | Diethyl azodicarboxylate | 33.33 | 31.86 |  | 1.47 |
| 1041 | t-Butylacetate | 21.38 | 22.89 | 22.85 | -1.47 |
| 1042 | Ethyl phenylacetate | 35.36 | 33.73 | 33.88 | 1.48 |
| 1043 | Ethyl cyclopentane-1,1-diacetate | 32.41 | 33.85 | 33.90 | -1.49 |
| 1044 | Ethyl cyclobutanecarboxylate | 29.86 | 28.39 | 28.37 | 1.49 |
| 1045 | 3-Hexanol | 24.51 | 26.04 | 26.00 | -1.49 |
| 1046 | Bicyclopentyl | 32.74 | 31.26 | 31.25 | 1.49 |
| 1047 | N-Butyl-3-methylpyridinium trifluoromethanesulfonate | 38.60 | 37.44 | 37.11 | 1.49 |
| 1048 | Pentyl vinyl ether | 23.44 | 22.40 | 21.95 | 1.49 |
| 1049 | Propanenitrile | 26.75 | 28.16 | 28.24 | -1.49 |
| 1050 | Ethyl benzoate | 35.04 | 33.53 | 33.55 | 1.49 |
| 1051 | 1-Pentyl-3-methylimidazolium lactate | 42.40 | 43.89 | 43.90 | -1.50 |
| 1052 | Cyclopentanone | 32.80 | 31.34 | 31.30 | 1.50 |
| 1053 | Dipropyl ether | 20.50 | 22.00 | 22.00 | -1.50 |
| 1054 | Hexa(2-ethyl-1-hexoxy)disiloxane | 26.80 | 27.46 | 28.30 | -1.50 |
| 1055 | 1-(2-Pyridinyl)ethanone | 39.65 | 38.41 | 38.15 | 1.50 |
| 1056 | Butanesulfonyl chloride | 35.40 | 34.27 | 33.90 | 1.50 |
| 1057 | Methylenanthate | 25.99 | 27.45 | 27.49 | -1.50 |
| 1058 | 1-Methyl-1-hexylpyrrolidinium bis(trifluoromethanesulfonyl) amide | 31.70 | 32.92 | 33.20 | -1.50 |
| 1059 | Diethyl sulfite | 29.58 | 28.29 | 28.07 | 1.51 |
| 1060 | 1,3-Diheptylimidazolium bis(trifluoromethylsulfonyl) amide | 29.30 | 30.74 | 30.81 | -1.51 |
| 1061 | Diisopropyl disulfide | 28.37 | 27.36 | 26.85 | 1.52 |
| 1062 | Di-n-propylmaleate | 31.96 | 30.52 | 30.44 | 1.52 |
| 1063 | 3-Methyl-2-butanol | 23.20 | 24.72 | 24.72 | -1.52 |
| 1064 | Diisopentylamine | 23.81 | 22.02 | 22.28 | 1.53 |
| 1065 | 3-(Ethylamino)propionitrile | 32.83 | 31.44 | 31.30 | 1.53 |
| 1066 | 2-Ethoxyethanol | 28.80 | 27.38 | 27.27 | 1.53 |
| 1067 | Ethyl formate | 23.84 | 25.26 | 25.37 | -1.53 |
| 1068 | Dimethyl propylmalonate | 31.33 | 32.80 | 32.87 | -1.54 |
| 1069 | Cyclopentyl acetate | 30.62 | 29.19 | 29.08 | 1.54 |
| 1070 | Dioctyl sulfide | 30.25 | 28.88 | 28.71 | 1.54 |
| 1071 | Diethyl sulfate | 33.52 | 32.36 | 31.98 | 1.54 |
| 1072 | 4-Ethylpyridine | 33.72 | 32.17 | 32.18 | 1.54 |
| 1073 | Dipropyl 1,1-cyclobutanedicarboxylate | 30.67 | 32.22 | 32.21 | -1.54 |
| 1074 | 1-Chlorohexane | 26.24 | 27.79 | 27.78 | -1.54 |
| 1075 | Propyl crotonate | 27.83 | 26.15 | 26.28 | 1.55 |
| 1076 | Perfluoro(propylcyclohexane) | 17.20 | 18.79 | 18.75 | -1.55 |
| 1077 | Dipentyl disulfide | 29.61 | 30.96 | 31.16 | -1.55 |
| 1078 | 1,1,1-Trichloroethane | 25.18 | 26.66 | 26.73 | -1.55 |
| 1079 | N-Propyl-3-methylpyridinium bis(trifluoromethylsulfonyl)amide | 34.70 | 33.40 | 33.15 | 1.55 |
| 1080 | 1-Pentyl-3-methylimidazolium glycine | 41.90 | 43.37 | 43.45 | -1.55 |
| 1081 | (Ethylthio)benzene | 37.04 | 35.40 | 35.48 | 1.56 |
| 1082 | 2-Picoline | 33.62 | 32.21 | 32.06 | 1.56 |
| 1083 | Butyl crotonate | 27.84 | 26.35 | 26.28 | 1.56 |
| 1084 | 3-Ethoxypropanenitrile | 31.52 | 29.98 | 29.96 | 1.56 |
| 1085 | Ethylnitrate | 28.12 | 29.90 | 29.68 | -1.56 |
| 1086 | 1,3-Dibutylimidazolium bis(trifluoromethylsulfonyl) amide | 31.10 | 29.54 | 29.54 | 1.56 |
| 1087 | 1,1-Bis(2-ethoxyethoxy)ethane | 27.44 | 26.04 | 25.88 | 1.56 |
| 1088 | 1,2-Dimethoxycyclopentane | 31.90 | 33.34 | 33.47 | -1.57 |
| 1089 | Bis(1,H,1H-heptafluorobutyl) adipate | 20.40 | 21.92 | 21.97 | -1.57 |
| 1090 | 1-Methyl-4-isopropylcyclohexene | 27.16 | 25.57 | 25.59 | 1.57 |
| 1091 | Bromobenzene | 35.24 | 36.48 | 36.81 | -1.57 |
| 1092 | Propyl decanoate | 27.41 | 25.75 | 25.83 | 1.58 |
| 1093 | 2-Ethyltoluene | 30.20 | 28.70 | 28.62 | 1.58 |
| 1094 | Diethyl acetylenedicarboxylate | 33.31 | 31.60 | 31.73 | 1.58 |
| 1095 | Tetraethyleneglycol hexyl ether | 32.10 | 33.64 | 33.68 | -1.58 |
| 1096 | Dibutyl suberate | 31.69 | 30.12 | 30.11 | 1.58 |
| 1097 | 1-Ethylimidazolium acetate | 37.80 | 39.38 |  | -1.58 |
| 1098 | N-Butyl-4-methylpyridinium bis(trifluoromethylsulfonyl)amide | 35.40 | 33.60 | 33.81 | 1.59 |
| 1099 | 1,9-Diiodononane | 42.51 | 43.80 | 44.10 | -1.59 |
| 1100 | Isopentyl hydrocinnamate | 31.75 | 33.41 | 33.34 | -1.59 |
| 1101 | Ethyl 2-naphthaleneacetate | 40.67 | 39.05 | 39.07 | 1.60 |
| 1102 | 2-Iodobutane | 28.21 | 29.44 | 29.81 | -1.60 |
| 1103 | 1,8-Dibromooctane | 39.84 | 38.36 | 38.23 | 1.61 |
| 1104 | 1-(2-Hydroxyethyl)-3-methylimidazolium tris(pentafluoroethyl)trifluorophosphate | 38.70 | 37.97 | 37.09 | 1.61 |
| 1105 | Isovaleronitrile | 25.93 | 27.44 | 27.54 | -1.61 |
| 1106 | Cyclohexyl formate | 32.22 | 30.70 | 30.60 | 1.62 |
| 1107 | 2-Methyl-2-pentanol | 23.35 | 24.90 | 24.97 | -1.62 |
| 1108 | n-Butyldichloroacetate | 30.49 | 31.98 | 32.11 | -1.62 |
| 1109 | 1,1-Diisopropoxyethane | 20.97 | 22.16 | 22.59 | -1.62 |
| 1110 | Butyric acid methylester | 25.19 | 26.85 | 26.82 | -1.63 |
| 1111 | Dioctyl phosphite | 28.97 | 30.18 | 30.60 | -1.63 |
| 1112 | Trihexyl tetradecyl phosphonium chloride | 30.60 | 31.82 | 32.23 | -1.63 |
| 1113 | Tetraethylmethane | 22.83 | 21.10 | 21.19 | 1.64 |
| 1114 | Methyl 3-methylbutyrate | 24.24 | 25.93 | 25.88 | -1.64 |
| 1115 | Dipropyl oxalate | 30.19 | 28.52 | 28.55 | 1.64 |
| 1116 | Dimethyl cyclobutane-1,1-dicarboxylate | 35.04 | 36.82 | 36.68 | -1.64 |
| 1117 | n-Heptylamine | 24.36 | 25.92 | 26.00 | -1.64 |
| 1118 | Propargyl propionate | 31.25 | 29.67 | 29.61 | 1.64 |
| 1119 | Cyanoethylene | 27.30 | 28.81 | 28.94 | -1.64 |
| 1120 | N-Ethylpyridinium bis(trifluoromethylsulfonyl)amide | 35.60 | 34.24 | 33.95 | 1.65 |
| 1121 | 1-Iodooctane | 30.68 | 32.29 | 32.33 | -1.65 |
| 1122 | Heptyl dihydrogen phosphate | 28.04 | 29.36 | 29.69 | -1.65 |
| 1123 | Butyl 1-naphthylketone | 39.31 | 41.48 | 40.97 | -1.66 |
| 1124 | Dibutyl adipate | 31.37 | 29.72 | 29.71 | 1.66 |
| 1125 | Tris(2-ethoxyethyl) phosphite | 30.62 | 28.86 | 28.96 | 1.66 |
| 1126 | 2,3,3-Trimethylhexane | 21.50 | 19.98 | 19.84 | 1.66 |
| 1127 | Diethyl bromomalonate | 33.08 | 34.49 | 34.74 | -1.66 |
| 1128 | Ethylenedichloride | 32.57 | 34.20 | 34.23 | -1.66 |
| 1129 | Bis(2-methoxyethyl)phosphite | 37.87 | 36.42 | 36.20 | 1.67 |
| 1130 | 1-Phenyl-1-propanone | 37.37 | 35.76 | 35.70 | 1.67 |
| 1131 | 3-Ethylpyridine | 33.77 | 32.17 | 32.10 | 1.67 |
| 1132 | Octamethyltrisiloxane | 17.56 | 16.38 | 15.89 | 1.67 |
| 1133 | 1-Hexyl-3-methylimidazolium dicyanoamide | 43.00 | 44.72 | 44.67 | -1.67 |
| 1134 | Styrene | 32.00 | 30.37 | 30.32 | 1.68 |
| 1135 | s-Butyl nicotinate | 34.42 | 36.22 | 36.10 | -1.68 |
| 1136 | Butyl dihydrogen phosphate | 27.38 | 28.76 | 29.06 | -1.68 |
| 1137 | 1-Nonyne | 25.01 | 26.68 | 26.70 | -1.69 |
| 1138 | Bis(7-H-perfluoroheptyl) pinate | 26.20 | 27.64 | 27.89 | -1.69 |
| 1139 | Isohexanoic acid | 26.60 | 25.06 | 24.90 | 1.70 |
| 1140 | Propyl glycolate | 31.55 | 29.93 | 29.85 | 1.70 |
| 1141 | 2-Isobutyrylthiophene | 37.09 | 35.43 | 35.39 | 1.70 |
| 1142 | Pentyl crotonate | 28.27 | 26.55 | 26.57 | 1.70 |
| 1143 | Perfluoro-n-octane | 13.70 | 11.98 | 12.00 | 1.70 |
| 1144 | 1-Methoxyhexane | 23.21 | 24.90 | 24.91 | -1.70 |
| 1145 | Propylcyclopentane | 24.95 | 26.58 | 26.65 | -1.70 |
| 1146 | Allyl acetate | 26.36 | 24.80 | 24.65 | 1.71 |
| 1147 | Xanthogenic acid ethylester | 33.72 | 32.43 | 32.01 | 1.71 |
| 1148 | Butyl decanoate | 27.66 | 25.95 | 25.94 | 1.72 |
| 1149 | Butyl butylxanthate | 31.89 | 33.23 | 33.61 | -1.72 |
| 1150 | Enanthic acid | 28.15 | 26.38 | 26.43 | 1.72 |
| 1151 | 2,4-Lutidine | 33.17 | 31.17 | 31.45 | 1.72 |
| 1152 | Tripenyl phosphite | 26.92 | 25.20 | 25.20 | 1.72 |
| 1153 | 3-Heptyloxypropanenitrile | 29.38 | 30.98 | 31.10 | -1.72 |
| 1154 | 2-Butylpyridine | 31.02 | 32.81 | 32.74 | -1.72 |
| 1155 | Pentyl fumarate | 30.69 | 32.15 | 32.42 | -1.73 |
| 1156 | Methylenecyclopentane | 24.35 | 25.98 | 26.08 | -1.73 |
| 1157 | Ethyl decanoate | 27.23 | 25.55 | 25.49 | 1.74 |
| 1158 | Methyl isobutyl ketone | 23.64 | 25.46 | 25.38 | -1.74 |
| 1159 | 3-(Dipropylamino)propionitrile | 28.99 | 30.63 | 30.73 | -1.74 |
| 1160 | Isobutylbenzene | 27.47 | 29.04 | 29.21 | -1.74 |
| 1161 | cis-2-Heptene | 20.81 | 22.52 | 22.56 | -1.75 |
| 1162 | n-Octylamine | 27.73 | 26.12 | 25.98 | 1.75 |
| 1163 | 1,2,4-Trimethylbenzene | 29.20 | 27.46 | 27.45 | 1.75 |
| 1164 | 1-Ethyl-3-methylimidazolium pentyltrifluoroborate | 33.80 | 35.55 |  | -1.75 |
| 1165 | 2-Butanone | 24.52 | 26.18 | 26.28 | -1.76 |
| 1166 | 1-Fluoroheptane | 22.82 | 21.41 | 21.06 | 1.76 |
| 1167 | 2-Chloronitrobenzene | 45.77 | 44.06 | 44.01 | 1.76 |
| 1168 | 1,2,3-Trichloropropane | 37.05 | 38.88 | 38.82 | -1.77 |
| 1169 | Triheptyl phosphite | 28.15 | 26.40 | 26.37 | 1.78 |
| 1170 | Methyl 3-methyl-1,1-cyclohexanedicarboxylate | 33.36 | 35.23 | 35.14 | -1.78 |
| 1171 | cis-2,4,6-Trimethyl-1,3,5-trioxane | 26.16 | 26.74 | 27.95 | -1.79 |
| 1172 | Trihexyl phosphite | 27.54 | 25.80 | 25.75 | 1.79 |
| 1173 | 1-Ethyl-3-methylimidazolium thiocyanate | 49.00 | 47.58 | 47.20 | 1.80 |
| 1174 | Isopentyl fumarate | 29.33 | 31.03 | 31.13 | -1.80 |
| 1175 | 1-Iodoheptane | 30.38 | 32.09 | 32.18 | -1.80 |
| 1176 | Butoxybenzene | 31.45 | 33.13 | 33.25 | -1.80 |
| 1177 | Butyl isonicotinate | 34.77 | 36.54 | 36.57 | -1.80 |
| 1178 | 2-Pentylpyridine | 30.98 | 33.01 | 32.78 | -1.80 |
| 1179 | 3,4-Dimethylisoxazole | 35.76 | 34.68 | 33.95 | 1.81 |
| 1180 | Diamylamine | 26.15 | 24.26 | 24.34 | 1.81 |
| 1181 | 3-Methylcyclopentanol | 28.24 | 29.88 | 30.05 | -1.81 |
| 1182 | Butyl 1-naphthaleneacetate | 37.61 | 39.45 | 39.42 | -1.81 |
| 1183 | 1-Propyl-3-methylimidazolium tetrafluoroborate | 47.00 | 44.77 | 45.19 | 1.81 |
| 1184 | 4-(1-Ethylpropyl)pyridine | 33.22 | 31.65 | 31.40 | 1.82 |
| 1185 | Bis(2-chloroethyl) ether | 37.96 | 36.02 | 36.14 | 1.82 |
| 1186 | 2-Hydroxybicyclopentyl | 36.18 | 34.52 | 34.36 | 1.82 |
| 1187 | Isopentyl crotonate | 27.25 | 25.43 | 25.42 | 1.83 |
| 1188 | 1-Pentanesulfonylchloride | 33.10 | 34.47 | 34.93 | -1.83 |
| 1189 | Ethyl nicotinate | 37.80 | 36.14 | 35.97 | 1.83 |
| 1190 | 1,1-Dichloroacetone | 31.91 | 33.61 | 33.74 | -1.83 |
| 1191 | Mustard gas | 42.82 | 40.90 | 40.98 | 1.84 |
| 1192 | Dipropyl succinate | 30.68 | 28.92 | 28.84 | 1.84 |
| 1193 | Isopropyl nicotinate | 33.82 | 36.02 | 35.66 | -1.84 |
| 1194 | 2-Methylheptane | 19.79 | 21.62 | 21.64 | -1.85 |
| 1195 | 2-Pentanol | 23.95 | 25.84 | 25.80 | -1.85 |
| 1196 | Methylchloroacetate | 35.29 | 33.66 | 33.44 | 1.85 |
| 1197 | Bis(2-pentoxyethyl)phosphite | 30.94 | 32.62 | 32.80 | -1.86 |
| 1198 | Acetic acid | 27.10 | 25.38 | 25.24 | 1.86 |
| 1199 | Pentyloxybenzene | 31.72 | 33.33 | 33.58 | -1.86 |
| 1200 | 1-Dodecanol | 29.41 | 27.56 | 27.55 | 1.86 |
| 1201 | 1,4-Bis(hexanoyloxy)-2-butyne | 31.50 | 33.20 | 33.36 | -1.86 |
| 1202 | N-Ethylaniline | 36.86 | 35.02 | 35.00 | 1.86 |
| 1203 | 1,3-Dimethylimidazolium bis(trifluoromethylsulfonyl) amide | 36.30 | 34.74 | 34.44 | 1.86 |
| 1204 | 2-Methylhexane | 19.29 | 21.20 | 21.16 | -1.87 |
| 1205 | 4-Heptanone oxime | 27.93 | 29.40 | 29.80 | -1.87 |
| 1206 | Diethyl ethylmalonate | 29.51 | 27.60 | 27.63 | 1.88 |
| 1207 | Cyclohexyl iodide | 36.25 | 34.80 | 34.37 | 1.88 |
| 1208 | 4-Methyl-2-pentanol | 23.03 | 24.92 | 24.92 | -1.89 |
| 1209 | Ethyl 3-ethoxypropionate | 27.84 | 25.97 | 25.94 | 1.90 |
| 1210 | Hexa(2-ethyl-1-butoxy)disiloxane | 26.10 | 25.06 | 24.20 | 1.90 |
| 1211 | Triacontane | 30.19 | 31.98 | 32.09 | -1.90 |
| 1212 | Cyclohexanol | 32.92 | 31.00 | 31.01 | 1.91 |
| 1213 | 2,4-Dimethylpentane | 18.15 | 20.08 | 20.06 | -1.91 |
| 1214 | Isobutyl fumarate | 28.93 | 30.83 | 30.84 | -1.91 |
| 1215 | 3-Hexylpyridine | 31.11 | 32.97 | 33.02 | -1.91 |
| 1216 | Ethyl 4-methylcyclohexane-1,1-diacetate | 31.30 | 33.13 | 33.22 | -1.92 |
| 1217 | Oxirane | 24.33 | 26.16 | 26.25 | -1.92 |
| 1218 | Bromocyclohexane | 33.90 | 31.71 | 31.97 | 1.93 |
| 1219 | 2,2,3,3-Tetrafluoro-1-propanol | 27.60 | 25.82 | 25.67 | 1.93 |
| 1220 | 6-Chlorododecane | 27.94 | 26.26 | 26.01 | 1.93 |
| 1221 | 3,3'-Bitolyl | 39.00 | 37.14 | 37.07 | 1.93 |
| 1222 | Butanoic anhydride | 28.44 | 29.89 | 30.37 | -1.93 |
| 1223 | 1-Ethyl-2-methoxycyclohexanol | 36.10 | 34.26 | 34.16 | 1.94 |
| 1224 | 2-Methyl-1-propanol | 22.98 | 24.84 | 24.92 | -1.94 |
| 1225 | N,N-Diethylbenzylamine | 29.79 | 31.43 | 31.74 | -1.95 |
| 1226 | Methyl 4-methyl-1,1-cyclohexanedicarboxylate | 33.29 | 35.23 | 35.24 | -1.95 |
| 1227 | 1,3-Diethylimidazolium diethylphosphate | 35.50 | 33.90 | 33.55 | 1.95 |
| 1228 | 1-Butyl-3-methylimidazolium methosulfate | 43.30 | 41.66 | 41.34 | 1.96 |
| 1229 | Di-s-butylsulfide | 25.81 | 24.48 | 23.85 | 1.96 |
| 1230 | Methyl hexanoate | 25.33 | 27.25 | 27.29 | -1.96 |
| 1231 | 3-Fluorotoluene | 29.80 | 27.95 | 27.84 | 1.96 |
| 1232 | Bis(perfluorooctyl) 3-methylglutarate | 19.50 | 17.74 | 17.54 | 1.96 |
| 1233 | N-Nitrosodipentylamine | 30.24 | 31.83 | 32.20 | -1.96 |
| 1234 | n-Amyl ether | 24.80 | 22.80 | 22.83 | 1.97 |
| 1235 | Tridecyl borate | 29.38 | 27.58 | 27.41 | 1.97 |
| 1236 | 1,3-Dioctylimidazolium bis(trifluoromethylsulfonyl) amide | 29.20 | 31.14 | 31.17 | -1.97 |
| 1237 | Ethyl 3-methylcyclohexane-1,1-diacetate | 31.26 | 33.13 | 33.23 | -1.97 |
| 1238 | 1-Methyl-1-propylpiperidinium bis(trifluoromethylsulfonyl)amide | 34.50 | 32.52 | 32.52 | 1.98 |
| 1239 | 2,4-Dimethyl-3-ethylpentane | 21.87 | 19.80 | 19.89 | 1.98 |
| 1240 | 4-(1-Hexylheptyl)pyridine | 31.27 | 33.25 | 33.25 | -1.98 |
| 1241 | 1-Undecanol | 25.46 | 27.36 | 27.44 | -1.98 |
| 1242 | 1-Propyl-3-methylimidazolium tris(pentafluoroethyl)trifluorophosphate | 34.10 | 32.39 | 32.12 | 1.98 |
| 1243 | Muscone | 34.59 | 32.42 | 32.59 | 2.00 |
| 1244 | 4-Chlorododecane | 28.01 | 26.26 | 26.01 | 2.00 |
| 1245 | Cyclohexyl acetate | 31.27 | 29.39 | 29.26 | 2.01 |
| 1246 | Bromocyclopentane | 33.31 | 31.51 | 31.30 | 2.01 |
| 1247 | 3-Methylthiophene | 32.33 | 30.90 | 30.32 | 2.01 |
| 1248 | 3-Fluoronitrobenzene | 37.88 | 39.71 | 39.89 | -2.01 |
| 1249 | 1-Pentyl-3-methylimidazolium L-alanine | 44.40 | 42.32 | 42.38 | 2.02 |
| 1250 | Hexyl perfluorobutyrate | 19.20 | 21.25 | 21.22 | -2.02 |
| 1251 | Isopentyl bromide | 26.11 | 27.95 | 28.13 | -2.02 |
| 1252 | 1-Iodohexane | 29.93 | 31.89 | 31.96 | -2.03 |
| 1253 | Methyl phenylacetate | 38.32 | 36.23 | 36.28 | 2.04 |
| 1254 | Heptane | 20.28 | 22.32 | 22.32 | -2.04 |
| 1255 | trans-2-Hexahydroindanone | 33.03 | 34.86 | 35.07 | -2.04 |
| 1256 | Trihexyl tetradecyl phosphonium methylsulfonate | 31.00 | 31.52 | 33.05 | -2.05 |
| 1257 | 2-(Bromomethyl)tetrahydrofuran | 37.11 | 35.13 | 35.06 | 2.05 |
| 1258 | 1,6-Hexanediol bis(perfluorooctanoate) | 20.60 | 18.72 | 18.55 | 2.05 |
| 1259 | 1-Butyl-1-methylpyrrolidinium thiocyanate | 49.80 | 48.36 | 47.75 | 2.05 |
| 1260 | 2-Propoxycyclohexanol | 34.70 | 32.70 | 32.65 | 2.05 |
| 1261 | 1,1-Di-p-tolylethane | 34.50 | 36.46 | 36.55 | -2.05 |
| 1262 | Butyl 2-naphthaleneacetate | 37.75 | 39.45 | 39.80 | -2.05 |
| 1263 | Isopropylphenylether | 30.81 | 32.61 | 32.87 | -2.06 |
| 1264 | alpha-Ethyl-beta-methylstyrene | 33.87 | 31.65 | 31.81 | 2.06 |
| 1265 | Dipropyl suberate | 31.87 | 29.72 | 29.81 | 2.06 |
| 1266 | Methyl 3-methylglutarate | 33.70 | 31.73 | 31.64 | 2.06 |
| 1267 | Cycloheptanol | 33.17 | 31.20 | 31.11 | 2.06 |
| 1268 | Isopentyliodide | 28.54 | 30.57 | 30.60 | -2.06 |
| 1269 | Methyl isobutanoate | 23.73 | 25.73 | 25.80 | -2.07 |
| 1270 | Dimethyl adipate | 35.86 | 33.92 | 33.79 | 2.07 |
| 1271 | N-Nitrosodimethylamine | 38.36 | 36.63 | 36.29 | 2.07 |
| 1272 | 3-Butenoic acid | 28.82 | 26.43 | 26.74 | 2.08 |
| 1273 | 2-Dodecanol | 29.16 | 27.24 | 27.08 | 2.08 |
| 1274 | Tetraethylene glycol octyl ether | 32.00 | 34.04 | 34.08 | -2.08 |
| 1275 | 3-Heptylpyridine | 31.25 | 33.17 | 33.33 | -2.08 |
| 1276 | 1-Iodopropane | 29.28 | 31.29 | 31.36 | -2.08 |
| 1277 | Methyl trioctylammonium bis(trifluoromethylsulfonyl)amide | 29.06 | 30.76 | 31.14 | -2.08 |
| 1278 | 2-Hexylnaphthalene | 33.95 | 36.00 | 36.04 | -2.09 |
| 1279 | Trioctyl borate | 28.18 | 26.38 | 26.09 | 2.09 |
| 1280 | Ethyl-n-propylether | 19.80 | 21.80 | 21.89 | -2.09 |
| 1281 | N-Butylaniline | 33.91 | 35.42 | 36.01 | -2.10 |
| 1282 | 3-(Dibutylamino)propionitrile | 29.19 | 31.03 | 31.29 | -2.10 |
| 1283 | 5-Chlorododecane | 28.08 | 26.26 | 25.98 | 2.10 |
| 1284 | Ethyl 2-O-benzoyllactate | 35.61 | 37.58 | 37.71 | -2.10 |
| 1285 | 2-Methyl-2-butanol | 22.68 | 24.70 | 24.78 | -2.10 |
| 1286 | Perfluoro-1,2-dimethylcyclohexane | 15.50 | 17.24 | 17.60 | -2.10 |
| 1287 | Heptyl nonanoate | 28.57 | 26.35 | 26.46 | 2.11 |
| 1288 | Methyl dipropylmalonate | 29.34 | 31.39 | 31.45 | -2.11 |
| 1289 | Octacosane | 29.71 | 31.14 | 31.82 | -2.11 |
| 1290 | Triethyl phosphate | 29.95 | 28.20 | 27.83 | 2.12 |
| 1291 | 1-Ethyl-3-methylimidazolium tricyanomethide | 47.90 | 45.78 |  | 2.12 |
| 1292 | Ethyl thiocyanate | 34.83 | 33.26 | 32.70 | 2.13 |
| 1293 | Diethyl O-butyrylmalate | 30.77 | 32.77 | 32.90 | -2.13 |
| 1294 | Ethyl acetoacetate | 32.40 | 30.35 | 30.27 | 2.13 |
| 1295 | Diethyleneglycol diethyl ether | 27.39 | 25.24 | 25.25 | 2.14 |
| 1296 | 3-Chloro-3-methylheptane | 23.53 | 25.62 | 25.67 | -2.14 |
| 1297 | 1-Propanol | 23.71 | 25.76 | 25.86 | -2.15 |
| 1298 | Iodocyclopentane | 36.19 | 34.60 | 34.03 | 2.16 |
| 1299 | Pentyl undecanoate | 28.62 | 26.35 | 26.46 | 2.16 |
| 1300 | 2,3,4-Trimethylhexane | 21.87 | 19.80 | 19.69 | 2.18 |
| 1301 | o-Cresol | 35.39 | 33.12 | 33.21 | 2.18 |
| 1302 | 2,2-Dimethylpentane | 18.03 | 20.26 | 20.22 | -2.19 |
| 1303 | Cyclohexane | 24.65 | 26.86 | 26.84 | -2.19 |
| 1304 | Dimethyl carbonate | 29.25 | 31.26 | 31.44 | -2.19 |
| 1305 | 1-Octylnaphthalene | 34.23 | 36.44 | 36.42 | -2.19 |
| 1306 | 2-Hexanoylthiophene | 34.83 | 36.95 | 37.02 | -2.19 |
| 1307 | Carvone | 34.70 | 32.81 | 32.50 | 2.20 |
| 1308 | 1,7-Dibromoheptane | 40.28 | 38.16 | 38.08 | 2.20 |
| 1309 | 1-Iodopentane | 29.38 | 31.69 | 31.58 | -2.20 |
| 1310 | Cyclopentanol | 33.02 | 30.80 | 30.81 | 2.21 |
| 1311 | 1-Methyl-1-decylpyrrolidinium bis(trifluoromethanesulfonyl) amide | 31.40 | 33.72 | 33.61 | -2.21 |
| 1312 | Isobutyl ricinoleate | 31.54 | 33.49 | 33.76 | -2.22 |
| 1313 | Butyl dodecanoate | 28.73 | 26.35 | 26.51 | 2.22 |
| 1314 | Pelargonic acid | 28.90 | 26.78 | 26.67 | 2.23 |
| 1315 | 1,3-Dinonylimidazolium bis(trifluoromethylsulfonyl) amide | 29.50 | 31.54 | 31.73 | -2.23 |
| 1316 | Cyclooctane | 29.84 | 27.70 | 27.61 | 2.23 |
| 1317 | Ethyl ethanesulfonate | 36.04 | 38.27 |  | -2.23 |
| 1318 | Trimethyl phosphate | 37.48 | 35.70 | 35.24 | 2.24 |
| 1319 | Pentyl phenyl sulfide | 34.16 | 36.00 | 36.40 | -2.24 |
| 1320 | beta-Ethyl-alpha-propylstyrene | 34.20 | 32.09 | 31.96 | 2.24 |
| 1321 | 2-Ethoxycyclohexanol | 34.70 | 32.50 | 32.45 | 2.25 |
| 1322 | Methyl isonicotinate | 40.83 | 38.64 | 38.58 | 2.25 |
| 1323 | trans-(1-Methyl-1-propenyl)benzene | 33.61 | 31.43 | 31.35 | 2.26 |
| 1324 | Nonyl heptanoate | 28.63 | 26.35 | 26.37 | 2.26 |
| 1325 | 1-Butyl-3-methylimidazolium hydrosulfate | 43.60 | 45.86 |  | -2.26 |
| 1326 | 11-Decyldocosane | 30.20 | 31.70 | 32.46 | -2.26 |
| 1327 | 1-Bromopentane | 26.80 | 29.07 | 29.06 | -2.26 |
| 1328 | 1,5-Cyclooctadiene | 31.46 | 29.30 | 29.19 | 2.27 |
| 1329 | 4-Benzyl-2-chlorophenol | 44.20 | 45.84 | 46.47 | -2.27 |
| 1330 | Pentyl 2-naphthoate | 37.20 | 39.45 | 39.47 | -2.27 |
| 1331 | Dipropyl sebacate | 32.38 | 30.12 | 30.11 | 2.27 |
| 1332 | Butylnitrate | 28.10 | 30.30 | 30.37 | -2.27 |
| 1333 | Methyl cis-cinnamate | 40.17 | 38.03 | 37.89 | 2.28 |
| 1334 | Methyl acetate | 24.08 | 26.45 | 26.36 | -2.28 |
| 1335 | Hexyl nitrite | 23.86 | 26.14 |  | -2.28 |
| 1336 | Bis(2-hexoxyethyl)phosphite | 31.18 | 33.02 | 33.46 | -2.28 |
| 1337 | 1-Tetradecyl-1,8-diazabicyclo(5,4,0)undec-7-enium dicyanamide | 30.25 | 32.01 | 32.53 | -2.28 |
| 1338 | 1-Methoxynaphthalene | 42.84 | 40.55 | 40.55 | 2.29 |
| 1339 | Diisopentyl adipate | 30.10 | 27.88 | 27.81 | 2.29 |
| 1340 | 2,2,3,4-Tetramethylpentane | 21.12 | 18.86 | 18.83 | 2.29 |
| 1341 | trans-4-Methylcyclohexanol | 27.69 | 30.08 | 29.98 | -2.29 |
| 1342 | Benzalchloride | 39.19 | 36.99 | 36.90 | 2.29 |
| 1343 | 1-Fluoropentane | 20.06 | 21.01 | 22.35 | -2.29 |
| 1344 | Phenylacetone | 38.00 | 35.76 | 35.70 | 2.30 |
| 1345 | Dimethyl sulfate | 38.93 | 37.36 | 36.63 | 2.30 |
| 1346 | 1,1-Dipentyloxyethane | 25.67 | 23.60 | 23.37 | 2.30 |
| 1347 | 4-(2-Chlorobenzyl)-phenol | 43.63 | 45.84 | 45.93 | -2.30 |
| 1348 | Ethyl dodecanoate | 28.32 | 25.95 | 26.01 | 2.31 |
| 1349 | 3-(Hexylamino)propionitrile | 29.93 | 32.24 | 32.24 | -2.31 |
| 1350 | 3,3,4-Trimethylhexane | 22.31 | 19.98 | 19.99 | 2.32 |
| 1351 | 2-Chloro-2-methylpentane | 22.93 | 25.22 | 25.25 | -2.32 |
| 1352 | Isobutyronitrile | 24.93 | 27.24 | 27.25 | -2.32 |
| 1353 | 1,3-Didecylimidazolium bis(trifluoromethylsulfonyl) amide | 29.60 | 31.94 | 31.93 | -2.33 |
| 1354 | Methylcyclohexane | 23.85 | 26.16 | 26.19 | -2.34 |
| 1355 | Diethyl benzylmalonate | 35.04 | 37.18 | 37.38 | -2.34 |
| 1356 | 2-Ethylnaphthalene | 37.10 | 35.12 | 34.75 | 2.35 |
| 1357 | Methyl 2-naphthaleneacetate | 43.85 | 41.55 | 41.50 | 2.35 |
| 1358 | 1-Butyl-4-methylpyridinium thiocyanate | 47.70 | 49.44 | 50.05 | -2.35 |
| 1359 | Ethyl 3-methylcyclopentane-1,1-diacetate | 30.68 | 32.93 | 33.05 | -2.37 |
| 1360 | alpha-Methylstyrene | 32.56 | 30.26 | 30.19 | 2.37 |
| 1361 | 2-Heptylnaphthalene | 33.85 | 36.22 | 36.22 | -2.37 |
| 1362 | 2,3-Dimethylbutane | 17.38 | 19.66 | 19.75 | -2.37 |
| 1363 | 2-Methylpropylamine | 22.30 | 24.20 | 24.67 | -2.37 |
| 1364 | 1-Decyl-1,8-diazabicyclo(5,4,0)undec-7-enium thiocyanate | 32.94 | 34.87 | 35.31 | -2.37 |
| 1365 | Pentyl 1-naphthoate | 37.20 | 39.45 | 39.58 | -2.38 |
| 1366 | Thiolane | 33.82 | 31.44 | 31.44 | 2.38 |
| 1367 | Diisopropyl sulfite | 26.14 | 28.05 | 28.52 | -2.38 |
| 1368 | Allyl bromide | 26.94 | 29.32 | 29.32 | -2.38 |
| 1369 | 2-Iodopropane | 27.42 | 29.24 | 29.80 | -2.38 |
| 1370 | Dimethyl decyl isopropylammonium bis(trifluoromethylsulfonyl)amide | 31.30 | 33.17 | 33.69 | -2.39 |
| 1371 | 2-Propanethiol | 21.91 | 24.09 | 24.30 | -2.39 |
| 1372 | Ethyl trans-cinnamate | 37.90 | 35.53 | 35.51 | 2.39 |
| 1373 | Dibutyl 1,1-cyclobutanedicarboxylate | 30.28 | 32.62 | 32.68 | -2.40 |
| 1374 | 3-Iodonitrobenzene | 47.40 | 49.80 |  | -2.40 |
| 1375 | 1-Butyl-3-methylimidazolium tetracyanoborate | 44.40 | 42.51 | 41.99 | 2.41 |
| 1376 | 1-Undecene | 24.86 | 22.45 | 22.44 | 2.42 |
| 1377 | Ethylcyclopentane | 23.88 | 26.16 | 26.30 | -2.42 |
| 1378 | trans-1,4-Dimethylcyclohexane | 23.02 | 25.46 | 25.45 | -2.43 |
| 1379 | 2-Ethoxyethyl dihydrogen phosphate | 32.20 | 30.18 | 29.76 | 2.44 |
| 1380 | Benzyl ether | 38.20 | 40.76 | 40.64 | -2.44 |
| 1381 | Pentyl 2-thiophenecarboxylate | 32.52 | 34.72 | 34.96 | -2.44 |
| 1382 | 4-Isopropylpyridine | 33.69 | 31.25 | 31.24 | 2.45 |
| 1383 | 2-Methoxycyclohexanol | 37.70 | 35.00 | 35.25 | 2.45 |
| 1384 | Propanoic anhydride | 29.70 | 29.49 | 27.25 | 2.45 |
| 1385 | 1,8-Octanediol dinitrate | 39.00 | 41.22 | 41.45 | -2.45 |
| 1386 | N-Pentylpyridinium bis(trifluoromethanesulfonyl) amide | 32.50 | 34.84 | 34.95 | -2.45 |
| 1387 | 2-Methoxy-1-methylcyclohexanol | 36.40 | 34.06 | 33.94 | 2.46 |
| 1388 | Diisopentyl succinate | 29.92 | 27.48 | 27.45 | 2.47 |
| 1389 | Tributylmethylammonium bis(trifluoromethylsulfonyl)amide | 30.60 | 28.36 | 28.13 | 2.47 |
| 1390 | Benzene | 28.22 | 30.52 | 30.69 | -2.47 |
| 1391 | 2,3-Lutidine | 33.24 | 31.17 | 30.76 | 2.48 |
| 1392 | Isobutyl iodide | 27.97 | 30.37 | 30.45 | -2.48 |
| 1393 | 2-Bromopropane | 23.84 | 26.15 | 26.32 | -2.48 |
| 1394 | Hexyl decanoate | 28.75 | 26.35 | 26.27 | 2.48 |
| 1395 | trans-2-Heptene | 20.10 | 22.52 | 22.58 | -2.48 |
| 1396 | Diisopentyl sebacate | 31.11 | 28.68 | 28.62 | 2.49 |
| 1397 | Ethyl iodide | 28.46 | 31.09 | 30.95 | -2.49 |
| 1398 | Methylcyclopentadecane | 32.33 | 29.94 | 29.83 | 2.50 |
| 1399 | 1-Naphthoic acid ethylester | 41.37 | 38.85 | 38.87 | 2.50 |
| 1400 | 4-(2,2,6-Trimethylcyclohexyl)-2-butanol | 30.57 | 28.10 | 28.06 | 2.51 |
| 1401 | 2,2,4,6,6-Pentamethylheptane | 21.40 | 19.18 | 18.89 | 2.51 |
| 1402 | 3-Chlorododecane | 28.58 | 26.26 | 26.07 | 2.51 |
| 1403 | Methyl 3-methyl-1,1-cyclopentanedicarboxylate | 32.56 | 35.03 | 35.07 | -2.51 |
| 1404 | Hexyloxybenzene | 31.23 | 33.53 | 33.74 | -2.51 |
| 1405 | Pentyl dihydrogen phosphate | 26.87 | 28.96 | 29.39 | -2.52 |
| 1406 | N-Nitrosodihexylamine | 30.07 | 32.23 | 32.60 | -2.53 |
| 1407 | Diethyl methylmalonate | 29.95 | 27.40 | 27.40 | 2.55 |
| 1408 | Ethyl 1-naphthaleneacetate | 41.09 | 39.05 | 38.54 | 2.55 |
| 1409 | Ethyl 2-cyano-3,3-dipropylacrylate | 32.43 | 34.67 | 34.98 | -2.55 |
| 1410 | 2-Chlorododecane | 28.62 | 26.26 | 26.06 | 2.56 |
| 1411 | 1-Dodecene | 25.15 | 22.67 | 22.58 | 2.57 |
| 1412 | 1,5-Diiodopentane | 45.54 | 43.00 | 42.97 | 2.57 |
| 1413 | 1-Butyl-3-ethylimidazolium diethylphosphate | 32.20 | 34.30 | 34.77 | -2.57 |
| 1414 | alpha-Pinene | 26.10 | 28.47 | 28.68 | -2.58 |
| 1415 | Bromocycloheptane | 34.42 | 31.91 | 31.84 | 2.58 |
| 1416 | 1,4-Bis(heptanoyloxy)-2-butyne | 31.23 | 33.60 | 33.81 | -2.58 |
| 1417 | n-Butylmethylsulfide | 26.23 | 29.30 | 28.81 | -2.58 |
| 1418 | Dipropyl adipate | 31.83 | 29.32 | 29.24 | 2.59 |
| 1419 | Pentyl nicotinate | 34.23 | 36.74 | 36.82 | -2.59 |
| 1420 | 3-Methylpentane | 18.14 | 20.78 | 20.73 | -2.59 |
| 1421 | Undecyl valerate | 28.86 | 26.35 | 26.27 | 2.59 |
| 1422 | Nitroethane | 32.13 | 29.89 | 29.53 | 2.60 |
| 1423 | Ethyl 3-phenylpropiolate | 39.18 | 37.01 | 36.56 | 2.62 |
| 1424 | Glyceryl tributanoate | 30.85 | 33.37 | 33.47 | -2.62 |
| 1425 | Di-n-propylsulfoxide | 32.60 | 35.22 |  | -2.62 |
| 1426 | trans-1-Propenylbenzene | 34.12 | 31.54 | 31.50 | 2.62 |
| 1427 | Hexyl 2-thiophenecarboxylate | 32.55 | 34.92 | 35.17 | -2.62 |
| 1428 | 1-Pentyl-3-methylimidazolium tetrafluoroborate | 42.90 | 45.17 | 45.52 | -2.62 |
| 1429 | 1-Chloronaphthalene | 41.04 | 38.66 | 38.41 | 2.63 |
| 1430 | 1,3-Dimethylimidazolium dimethylphosphate | 46.98 | 44.90 | 44.35 | 2.63 |
| 1431 | N-Butyl-3-methyl-pyridinium dicyanoamide | 43.40 | 45.78 | 46.03 | -2.63 |
| 1432 | 2-Chloro-2-methylbutane | 22.47 | 25.02 | 25.10 | -2.63 |
| 1433 | Dimethyl glutarate | 36.21 | 33.72 | 33.58 | 2.63 |
| 1434 | 2-Heptanoylthiophene | 34.36 | 37.15 | 36.99 | -2.63 |
| 1435 | Dicumenylmethane | 33.50 | 36.00 | 36.13 | -2.63 |
| 1436 | Methyl citraconate | 36.34 | 33.92 | 33.70 | 2.64 |
| 1437 | 1-Propyl-3-methylimidazolium aminoacetate | 45.60 | 42.97 | 42.96 | 2.64 |
| 1438 | Dibutylsulfate | 31.15 | 33.16 | 33.79 | -2.64 |
| 1439 | Bis(2-heptoxyethyl)phosphite | 30.95 | 33.42 | 33.59 | -2.64 |
| 1440 | 2-Chlorophenol | 39.70 | 37.10 | 37.05 | 2.65 |
| 1441 | Diethyl maleate | 32.59 | 30.12 | 29.94 | 2.65 |
| 1442 | Propyl 10-undecenoate | 29.49 | 26.60 | 26.84 | 2.65 |
| 1443 | s-Butylamine | 21.64 | 24.07 | 24.30 | -2.66 |
| 1444 | 3-(Pentylamino)propionitrile | 29.51 | 32.04 | 32.17 | -2.66 |
| 1445 | Diethylamine | 20.42 | 23.06 | 23.08 | -2.66 |
| 1446 | Ethyl undecanoate | 28.34 | 25.75 | 25.67 | 2.67 |
| 1447 | Tris(2-methoxyethyl) phosphite | 33.94 | 36.36 | 36.62 | -2.68 |
| 1448 | 1-Chloropentane | 24.94 | 27.59 | 27.62 | -2.68 |
| 1449 | 2,3,3,4-Tetramethylpentane | 21.55 | 18.86 | 18.86 | 2.69 |
| 1450 | 1-Hexyl-1,8-diazabicyclo(5,4,0)undec-7-enium dicyanamide | 32.57 | 30.41 | 29.88 | 2.69 |
| 1451 | Propyl dodecanoate | 28.81 | 26.15 | 26.11 | 2.70 |
| 1452 | 1,4-Di-s-butylbenzene | 29.98 | 27.56 | 27.27 | 2.71 |
| 1453 | 1-Propylamine | 22.37 | 25.12 | 25.08 | -2.71 |
| 1454 | Butyl iodoacetate | 33.09 | 35.66 | 35.80 | -2.71 |
| 1455 | Dimethyl pentylmalonate | 30.50 | 33.20 | 33.22 | -2.72 |
| 1456 | 2-Pentyl 2-bromobutyrate | 28.35 | 30.80 | 31.07 | -2.72 |
| 1457 | 1-Octyne | 23.86 | 26.46 | 26.58 | -2.72 |
| 1458 | Pentyl 2-picolinate | 34.62 | 36.98 | 37.34 | -2.72 |
| 1459 | Bis(2-ethoxyethyl)phosphite | 33.96 | 31.42 | 31.23 | 2.73 |
| 1460 | 1,5-Pentanediol dinitrate | 43.05 | 40.62 | 40.32 | 2.73 |
| 1461 | Pentyl 2-naphthaleneacetate | 37.04 | 39.65 | 39.77 | -2.73 |
| 1462 | Perfluoro-1,3,5-trimethylcyclohexane | 17.20 | 14.89 | 14.46 | 2.74 |
| 1463 | 2-Hexylpyridine | 30.62 | 33.21 | 33.36 | -2.74 |
| 1464 | 1-Hexyl-3-methylimidazolium glycine | 40.60 | 43.57 | 43.34 | -2.74 |
| 1465 | Diisoamyl ether | 23.02 | 20.56 | 20.27 | 2.75 |
| 1466 | 1-Iodonaphthalene | 47.15 | 44.40 |  | 2.75 |
| 1467 | Butyl 10-undecenoate | 29.61 | 26.80 | 26.85 | 2.76 |
| 1468 | 1-(2-Methoxyethyl)-1-methylpyrrolidinium tris(pentafluoroethyl)trifluorophosphate | 34.42 | 36.89 | 37.18 | -2.76 |
| 1469 | 1,2-Dibromoethane | 39.55 | 37.16 | 36.79 | 2.76 |
| 1470 | Methyl 1-naphthoate | 44.09 | 41.35 | 41.33 | 2.76 |
| 1471 | 2-Hydroxyacetophenone | 41.94 | 39.32 | 39.17 | 2.77 |
| 1472 | Ethyl 10-undecenoate | 29.24 | 26.40 | 26.47 | 2.77 |
| 1473 | 1-Iodobutane | 28.76 | 31.49 | 31.53 | -2.77 |
| 1474 | 1,6-Dibromohexane | 40.33 | 37.96 | 37.55 | 2.78 |
| 1475 | Triisobutylamine | 22.30 | 19.69 | 19.52 | 2.78 |
| 1476 | Methyl-n-propylketone | 23.59 | 26.38 | 26.37 | -2.78 |
| 1477 | 2-Ethoxycyclopentanol | 35.00 | 32.30 | 32.21 | 2.79 |
| 1478 | Decyl hexanoate | 29.06 | 26.35 | 26.27 | 2.79 |
| 1479 | 1-Bromopropane | 25.86 | 28.67 | 28.65 | -2.79 |
| 1480 | Methyl propanoate | 23.81 | 26.65 | 26.61 | -2.80 |
| 1481 | 1,1-Diisopentyloxyethane | 23.98 | 21.36 | 21.18 | 2.80 |
| 1482 | 1,9-Nonanediol dinitrate | 38.80 | 41.42 | 41.60 | -2.80 |
| 1483 | Benzyl benzoate | 45.95 | 43.11 | 43.15 | 2.80 |
| 1484 | 2,5-Dimethylthiophene | 30.45 | 28.87 | 27.64 | 2.81 |
| 1485 | Dihexylamine | 27.45 | 24.66 | 24.64 | 2.81 |
| 1486 | Icosafluorononane | 14.40 | 11.58 | 11.58 | 2.82 |
| 1487 | Methyl 4-methylcyclohexane-1,1-diacetate | 32.89 | 35.63 | 35.71 | -2.82 |
| 1488 | Dichloroacetic acid | 35.90 | 33.01 | 33.08 | 2.82 |
| 1489 | Pentyl isonicotinate | 33.92 | 36.74 | 36.74 | -2.82 |
| 1490 | trans-2-Butene-1,4-diol | 38.58 | 36.60 | 35.76 | 2.82 |
| 1491 | 1,2-Propanediol | 36.51 | 34.48 | 33.69 | 2.82 |
| 1492 | 2-Chlorobenzyl phenyl ether | 42.31 | 45.25 | 45.13 | -2.82 |
| 1493 | DMSO | 42.50 | 39.66 |  | 2.84 |
| 1494 | Aniline | 42.12 | 39.98 | 39.28 | 2.84 |
| 1495 | Methyl 3-methylcyclohexane-1,1-diacetate | 32.94 | 35.63 | 35.78 | -2.84 |
| 1496 | Methyl formate | 25.15 | 27.76 | 27.99 | -2.84 |
| 1497 | 1,5-Dibromopentane | 40.56 | 37.76 | 37.71 | 2.85 |
| 1498 | N,N-Diethylaniline | 34.51 | 32.02 | 31.66 | 2.85 |
| 1499 | 1-Ethyl-3-methylimidazolium tris(pentafluoroethyl)trifluorophosphate | 34.80 | 32.19 | 31.95 | 2.85 |
| 1500 | Lactonitrile | 36.39 | 33.62 | 33.54 | 2.85 |
| 1501 | 1-Ethyl-3-methylimidazolium propyltrifluoroborate | 38.00 | 35.15 |  | 2.85 |
| 1502 | Propenal | 23.14 | 25.65 | 25.99 | -2.85 |
| 1503 | (9Z)-Octadecenoic acid | 32.80 | 30.18 | 29.94 | 2.86 |
| 1504 | Methyl-n-pentylether | 21.92 | 24.70 | 24.78 | -2.86 |
| 1505 | 2-Heptylpyridine | 30.84 | 33.41 | 33.70 | -2.86 |
| 1506 | Hexyl 2-naphthoate | 36.79 | 39.65 | 39.67 | -2.88 |
| 1507 | 1-Ethyl-3-methylimidazolium dimethylphosphate | 44.30 | 41.90 | 41.41 | 2.89 |
| 1508 | Allyl succinate | 33.70 | 30.80 | 30.81 | 2.89 |
| 1509 | 1,2-Dichloropropane | 28.94 | 31.67 | 31.83 | -2.89 |
| 1510 | Methyl 2-thiophenecarboxylate | 39.33 | 36.62 | 36.42 | 2.91 |
| 1511 | 1-Propionaphthone | 43.49 | 41.08 | 40.57 | 2.92 |
| 1512 | Ethyl 2-naphthoate | 41.64 | 38.85 | 38.72 | 2.92 |
| 1513 | Isobutyl bromide | 24.84 | 27.75 | 27.76 | -2.92 |
| 1514 | 3,5-Lutidine | 33.82 | 30.93 | 30.89 | 2.93 |
| 1515 | Tri-n-pentylamine | 26.24 | 23.65 | 23.30 | 2.94 |
| 1516 | 2,4-Dichlorotoluene | 37.94 | 35.24 | 35.00 | 2.94 |
| 1517 | 1-Bromobutane | 25.90 | 28.87 | 28.86 | -2.96 |
| 1518 | 2,3-Dimethyl-3-ethylpentane | 22.94 | 19.98 | 19.98 | 2.96 |
| 1519 | Propyl tridecanoate | 29.30 | 26.35 | 26.34 | 2.96 |
| 1520 | Nitrobenzene | 44.02 | 41.12 | 41.05 | 2.97 |
| 1521 | Pentaerythritol tetra(perfluorobutyrate) | 18.50 | 21.00 | 21.47 | -2.97 |
| 1522 | 1,1-Dichloroethane | 24.66 | 27.41 | 27.63 | -2.97 |
| 1523 | Epichlorohydrin | 36.04 | 33.25 | 33.06 | 2.98 |
| 1524 | 3-(1-Methyl-2-pyrrolidinyl)pyridine | 38.85 | 40.95 | 41.83 | -2.98 |
| 1525 | N-Nitrosodiethylamine | 33.07 | 30.63 | 30.09 | 2.98 |
| 1526 | Perfluorotripropylamine | 15.20 | 18.19 |  | -2.99 |
| 1527 | Vinylidenechloride | 21.66 | 24.65 |  | -2.99 |
| 1528 | Diethyl sebacate | 32.76 | 29.72 | 29.76 | 3.00 |
| 1529 | cis-1,3-Dimethylcyclohexane | 22.63 | 25.46 | 25.63 | -3.00 |
| 1530 | Di-s-butylamine | 22.70 | 21.36 | 19.70 | 3.00 |
| 1531 | 1-Hexyl-3-methylimidazolium chloride | 41.80 | 43.70 | 44.80 | -3.00 |
| 1532 | Trimethyl decylammonium bis(trifluoromethylsulfonyl)amide | 35.42 | 37.60 | 38.43 | -3.01 |
| 1533 | 1-Propyl-3-methylimidazolium bis(trifluoromethanesulfonyl) amide | 34.87 | 31.94 | 31.86 | 3.01 |
| 1534 | Cyclopentyl methyl ether | 26.43 | 29.34 | 29.44 | -3.01 |
| 1535 | 1-Tridecene | 25.80 | 22.89 | 22.78 | 3.02 |
| 1536 | Perfluoro-1,2,4-trimethylcyclohexane | 17.30 | 14.89 | 14.28 | 3.02 |
| 1537 | 1-Hexyl-3-methylimidazolium tricyanomethanide | 43.55 | 46.58 |  | -3.03 |
| 1538 | Dodecyl butyrate | 29.32 | 26.35 | 26.29 | 3.03 |
| 1539 | 1-Fluorooctane | 24.03 | 21.61 | 20.99 | 3.04 |
| 1540 | Diethyl malate | 37.14 | 33.98 | 34.10 | 3.04 |
| 1541 | 3-(Dipentylamino)propionitrile | 28.73 | 31.43 | 31.77 | -3.04 |
| 1542 | Thioanisole | 40.33 | 37.82 | 37.28 | 3.05 |
| 1543 | Suberone | 34.66 | 31.74 | 31.61 | 3.05 |
| 1544 | Cyclohexanone | 34.57 | 31.54 | 31.51 | 3.06 |
| 1545 | 4-t-Butylpyridine | 33.58 | 30.51 | 30.49 | 3.09 |
| 1546 | cis-2-Hexene | 19.10 | 22.30 | 22.19 | -3.09 |
| 1547 | 1-Methylnaphthalene | 38.09 | 34.90 | 34.99 | 3.10 |
| 1548 | 1-Hexene | 18.52 | 21.35 | 21.62 | -3.10 |
| 1549 | Tris(2-ethylhexyl) tricarballylate | 29.60 | 32.21 | 32.70 | -3.10 |
| 1550 | 3,8-Diethyldecane | 26.10 | 23.02 | 22.99 | 3.11 |
| 1551 | Methanesulfonic acid | 50.50 | 47.39 |  | 3.11 |
| 1552 | 2-Octylnaphthalene | 33.68 | 36.44 | 36.80 | -3.12 |
| 1553 | Dimethyl succinate | 36.62 | 33.52 | 33.49 | 3.13 |
| 1554 | Tetrachloroethylene | 31.30 | 29.82 | 28.16 | 3.14 |
| 1555 | Methyl acrylate | 24.23 | 27.30 | 27.38 | -3.15 |
| 1556 | Tetraethylene glycol dodecyl ether | 31.70 | 34.84 | 34.86 | -3.16 |
| 1557 | Isopentyl chloride | 23.36 | 26.47 | 26.52 | -3.16 |
| 1558 | Trimethyl butylammonium bis(trifluoromethylsulfonyl)amide | 38.05 | 36.40 | 34.88 | 3.17 |
| 1559 | Ethanol | 22.39 | 25.56 | 25.56 | -3.17 |
| 1560 | Isopropyl formate | 22.27 | 25.14 | 25.45 | -3.18 |
| 1561 | Diethyl O-hexanoylmalate | 29.85 | 33.17 | 33.03 | -3.18 |
| 1562 | 1-(3-Aminopropyl)-3-methylimidazolium tetrafluoroborate | 47.10 | 49.91 | 50.28 | -3.18 |
| 1563 | 1-(2-Methoxyethyl)-1-methylpiperidinium tris(pentafluoroethyl)trifluorophosphate | 34.63 | 37.09 | 37.81 | -3.18 |
| 1564 | 3-Chloronitrobenzene | 46.87 | 44.06 | 43.68 | 3.19 |
| 1565 | Pentyl nitrite | 22.75 | 25.94 |  | -3.19 |
| 1566 | Hexyl 1-naphthoate | 36.58 | 39.65 | 39.78 | -3.20 |
| 1567 | Nitromethane | 36.53 | 34.31 | 33.32 | 3.21 |
| 1568 | n-Propylnitrate | 27.20 | 30.10 | 30.41 | -3.21 |
| 1569 | Ethyl thiophene-2-carboxylate | 36.97 | 34.12 | 33.76 | 3.21 |
| 1570 | Acetone O-methyloxime | 22.85 | 25.87 | 26.07 | -3.22 |
| 1571 | 3-Methylpyridine | 35.04 | 31.97 | 31.81 | 3.23 |
| 1572 | Pentyl 1-naphthylketone | 38.56 | 41.68 | 41.79 | -3.23 |
| 1573 | Methyl 3-phenylhydracrylate | 38.91 | 41.89 | 42.15 | -3.24 |
| 1574 | 1,2-Dicyclohexyloxycyclohexane | 36.50 | 39.42 | 39.74 | -3.24 |
| 1575 | 1-Octyl-3-methylimidazolium hexafluorophosphate | 36.50 | 39.18 | 39.74 | -3.24 |
| 1576 | Diethyl suberate | 32.60 | 29.32 | 29.35 | 3.25 |
| 1577 | Trihexyl tetradecyl phosphonium dicyanamide | 34.85 | 32.84 | 31.60 | 3.25 |
| 1578 | Dimethylamine | 26.79 | 29.06 | 30.04 | -3.25 |
| 1579 | Benzenesulfonylchloride | 43.25 | 43.37 | 39.99 | 3.26 |
| 1580 | 3,4-Xylenol | 28.92 | 32.08 | 32.18 | -3.26 |
| 1581 | m-Cresol | 35.69 | 33.12 | 32.43 | 3.26 |
| 1582 | Dimethylpropylisopropylammonium bis(trifluoromethylsulfonyl)amide | 34.30 | 31.77 | 31.04 | 3.26 |
| 1583 | N,N-Dimethylaniline | 36.04 | 38.02 | 39.30 | -3.26 |
| 1584 | 1-Butyl-3-methylimidazolium ethosulfate | 36.38 | 39.16 | 39.65 | -3.27 |
| 1585 | Pentadecyl formate | 30.89 | 27.86 | 27.62 | 3.27 |
| 1586 | 2-(2-Methoxyethoxy)ethanol | 28.49 | 31.70 | 31.76 | -3.27 |
| 1587 | 1,3-Dipropylimidazolium bis(trifluoromethylsulfonyl) amide | 32.40 | 29.14 | 29.12 | 3.28 |
| 1588 | Bis(cyclohexyl)amine | 33.74 | 32.08 | 30.46 | 3.28 |
| 1589 | Perfluoropentane | 9.89 | 13.18 | 13.17 | -3.28 |
| 1590 | Butylisothiocyanate | 30.42 | 33.71 |  | -3.29 |
| 1591 | Diethyl O-valerylmalate | 29.81 | 32.97 | 33.10 | -3.29 |
| 1592 | 2-Butanol | 22.54 | 25.64 | 25.83 | -3.29 |
| 1593 | 1-Propyl-2,3-dimethylimidazolium bis(trifluoromethanesulfonyl) amide | 41.00 | 37.70 |  | 3.30 |
| 1594 | Ethyl trans-decalin-2,2-diacetate | 34.39 | 37.57 | 37.69 | -3.30 |
| 1595 | 1-Octyl-1,8-diazabicyclo(5,4,0)undec-7-enium thiocyanate | 31.85 | 34.47 | 35.15 | -3.30 |
| 1596 | Dimethyl maleate | 38.29 | 35.12 | 34.98 | 3.31 |
| 1597 | Isobutyl propanoate | 26.64 | 23.43 | 23.33 | 3.31 |
| 1598 | Diethyl succinate | 31.89 | 28.52 | 28.57 | 3.32 |
| 1599 | Pentyl cyanoacetate | 29.77 | 32.93 | 33.11 | -3.34 |
| 1600 | Hexyl nicotinate | 33.71 | 36.94 | 37.05 | -3.34 |
| 1601 | 1-Ethylnaphthalene | 38.47 | 35.12 | 35.12 | 3.35 |
| 1602 | 2-Methoxycyclopentanol | 38.10 | 34.80 | 34.74 | 3.36 |
| 1603 | Ethyl 2-picolinate | 39.72 | 36.38 | 36.36 | 3.36 |
| 1604 | Diheptyl ether | 26.99 | 23.60 | 23.63 | 3.36 |
| 1605 | Methyl 3-methylcyclopentane-1,1-diacetate | 32.12 | 35.43 | 35.49 | -3.37 |
| 1606 | Ethyl myristate | 29.78 | 26.35 | 26.40 | 3.38 |
| 1607 | Diethyl O-heptanoylmalate | 29.84 | 33.37 | 33.22 | -3.38 |
| 1608 | 2-Methylpentane | 17.38 | 20.78 | 20.76 | -3.38 |
| 1609 | Diethyl malonate | 31.71 | 28.32 | 28.31 | 3.40 |
| 1610 | 1,3-Butyleneglycol | 37.80 | 34.68 | 34.40 | 3.40 |
| 1611 | 2-Propionylthiophene | 39.55 | 36.35 | 36.15 | 3.40 |
| 1612 | 1,4-Dibromobutane | 40.86 | 37.56 | 37.45 | 3.41 |
| 1613 | Diethyl phosphite | 30.96 | 27.78 | 27.55 | 3.41 |
| 1614 | 1-Pentyl-3-methylimidazolium hexafluorophosphate | 41.24 | 38.58 | 37.83 | 3.41 |
| 1615 | Benzyl 2-chlorophenyl ether | 42.16 | 45.25 | 45.58 | -3.42 |
| 1616 | Hexyl 2-picolinate | 34.12 | 37.18 | 37.54 | -3.42 |
| 1617 | 1-(2-Hydroxyethyl)-3-methylimidazolium bis(trifluoromethanesulfonyl) amide | 34.00 | 37.52 | 37.44 | -3.44 |
| 1618 | Bis(9-H-perfluorononyl) 3-t-butyladipate | 24.90 | 22.40 | 21.46 | 3.44 |
| 1619 | 3-Methylcyclopentene | 22.68 | 26.14 | 26.13 | -3.45 |
| 1620 | 4-Methylpyridine | 35.43 | 31.97 | 31.98 | 3.45 |
| 1621 | Pristane | 26.24 | 22.88 | 22.79 | 3.45 |
| 1622 | Diethyl adipate | 32.38 | 28.92 | 28.91 | 3.47 |
| 1623 | 1-Butyl-3-methylimidazolium hexafluorophosphate | 40.90 | 38.38 | 37.43 | 3.47 |
| 1624 | 1-Cyanonaphthalene | 46.40 | 42.86 | 42.92 | 3.48 |
| 1625 | 3-Methoxypropionitrile | 35.88 | 32.48 | 32.40 | 3.48 |
| 1626 | Ethyl trans-hexahydroindane-2,2-diacetate | 33.91 | 37.37 | 37.40 | -3.49 |
| 1627 | p-Toluidine | 36.06 | 38.94 | 39.55 | -3.49 |
| 1628 | 1,5-Hexadiene | 18.87 | 22.00 | 22.37 | -3.50 |
| 1629 | Diethyl O-nonanoylmalate | 30.41 | 33.77 | 33.92 | -3.51 |
| 1630 | 4-s-Butylpyridine | 34.71 | 31.45 | 31.19 | 3.52 |
| 1631 | Tetramethylsilane | 12.85 | 13.83 | 16.37 | -3.52 |
| 1632 | Hexane | 18.40 | 21.90 | 21.92 | -3.52 |
| 1633 | Acetaldehyde | 21.18 | 24.80 | 24.71 | -3.53 |
| 1634 | 2,2,3,3,4,4,5,5-Octafluoro-1,6-hexanediol dibutyrate | 23.90 | 27.32 | 27.45 | -3.55 |
| 1635 | Heptyl isonicotinate | 33.81 | 37.14 | 37.37 | -3.56 |
| 1636 | 1-Ethyl-3-methylimidazolium bis(trifluoromethanesulfonyl) amide | 35.10 | 31.74 | 31.53 | 3.57 |
| 1637 | Tridecyl propionate | 29.84 | 26.35 | 26.27 | 3.57 |
| 1638 | Phenylacetonitrile | 41.36 | 37.74 | 37.79 | 3.57 |
| 1639 | Diisopropyl ether | 17.80 | 21.36 | 21.37 | -3.57 |
| 1640 | Methylcyclopentane | 22.30 | 25.74 | 25.88 | -3.58 |
| 1641 | Methyl trans-decalin-2,2-diacetate | 36.41 | 40.07 | 40.00 | -3.59 |
| 1642 | Propargyl acetate | 32.81 | 29.47 | 29.22 | 3.59 |
| 1643 | Heptyl 2-thiophenecarboxylate | 31.72 | 35.12 | 35.33 | -3.61 |
| 1644 | Heptaethylene glycol | 48.39 | 45.52 | 44.78 | 3.61 |
| 1645 | Acetophenone | 39.04 | 35.56 | 35.43 | 3.61 |
| 1646 | 2,2,3,3-Tetramethylpentane | 22.49 | 19.04 | 18.88 | 3.61 |
| 1647 | Hexyl isonicotinate | 33.46 | 36.94 | 37.08 | -3.62 |
| 1648 | Neohexane | 16.31 | 19.84 | 19.93 | -3.62 |
| 1649 | 1,1-Dimethylcyclopentane | 21.75 | 25.22 | 25.37 | -3.62 |
| 1650 | 1-Butyl-2,3-dimethylimidazolium bis(trifluoromethanesulfonyl) amide | 34.26 | 37.90 |  | -3.64 |
| 1651 | 1-Heptyne | 22.68 | 26.24 | 26.32 | -3.64 |
| 1652 | Perfluoro-1-methyl-4-isopropylcyclohexane | 17.50 | 14.49 | 13.86 | 3.64 |
| 1653 | 1-Bromonaphthalene | 44.19 | 41.80 | 40.54 | 3.65 |
| 1654 | 1,2-Diethoxycyclopentane | 31.90 | 28.34 | 28.24 | 3.66 |
| 1655 | 1-Butyl-3-methylimidazolium iodide | 46.40 | 43.30 | 42.74 | 3.66 |
| 1656 | 1,4-Butanediol dinitrate | 43.80 | 40.42 | 40.14 | 3.66 |
| 1657 | Pentyl 1-naphthaleneacetate | 36.34 | 39.65 | 40.00 | -3.66 |
| 1658 | Diethyl glutarate | 32.32 | 28.72 | 28.64 | 3.68 |
| 1659 | 2-Methyloctanal | 21.44 | 25.08 | 25.12 | -3.68 |
| 1660 | Perfluorotrihexylamine | 18.30 | 14.59 |  | 3.71 |
| 1661 | 1-Chlorobutane | 23.74 | 27.39 | 27.46 | -3.72 |
| 1662 | 4-Methyl-2-thiapentane | 25.07 | 28.18 | 28.80 | -3.73 |
| 1663 | 1-Octyl-3-methylimidazolium tris(pentafluoroethyl)trifluorophosphate | 30.30 | 33.39 | 34.03 | -3.73 |
| 1664 | Heptyl nicotinate | 33.28 | 37.14 | 37.02 | -3.74 |
| 1665 | 1-Heptyl-3-methylimidazolium hexafluorophosphate | 35.89 | 38.98 | 39.65 | -3.76 |
| 1666 | 1,2-Dibutoxycyclohexane | 33.10 | 29.34 | 29.34 | 3.76 |
| 1667 | Hexyl 2-naphthaleneacetate | 36.22 | 39.85 | 39.98 | -3.76 |
| 1668 | Diethyl perfluoroadipate | 22.70 | 26.52 | 26.47 | -3.77 |
| 1669 | 1,6-Dimethylnaphthalene | 37.42 | 33.88 | 33.65 | 3.77 |
| 1670 | Diisopropoxymethane | 20.97 | 23.52 | 24.79 | -3.82 |
| 1671 | Cyclopentane | 22.61 | 26.44 | 26.43 | -3.82 |
| 1672 | 1-(Chloromethyl)naphthalene | 45.46 | 41.89 | 41.60 | 3.86 |
| 1673 | Octyl vinyl ether | 26.32 | 23.00 | 22.46 | 3.86 |
| 1674 | Tetradecyl acetate | 30.28 | 26.35 | 26.40 | 3.88 |
| 1675 | 1-Tetradecene | 26.75 | 23.11 | 22.87 | 3.88 |
| 1676 | Diethyl oxalate | 32.08 | 28.12 | 28.20 | 3.88 |
| 1677 | Trimethylphosphite | 27.18 | 30.90 | 31.06 | -3.88 |
| 1678 | 1,10-Decanediol dinitrate | 38.25 | 41.62 | 42.13 | -3.88 |
| 1679 | Cyclobutanecarboxylic acid | 33.62 | 29.82 | 29.72 | 3.90 |
| 1680 | Glyme | 24.61 | 28.42 | 28.52 | -3.91 |
| 1681 | Di-n-octylether | 27.72 | 24.00 | 23.79 | 3.93 |
| 1682 | 1-Pentadecene | 27.31 | 23.33 | 23.38 | 3.93 |
| 1683 | 2-Chlorobutane | 21.51 | 24.66 | 25.45 | -3.94 |
| 1684 | 2-Chloroethanol | 36.55 | 32.77 | 32.61 | 3.94 |
| 1685 | Dimethyl malonate | 37.37 | 33.32 | 33.40 | 3.97 |
| 1686 | trans-1,3-Butadienylbenzene | 36.21 | 32.41 | 32.23 | 3.98 |
| 1687 | Ethyl methyl sulfide | 25.06 | 28.90 | 29.05 | -3.99 |
| 1688 | Heptyl 2-picolinate | 33.28 | 37.38 | 37.27 | -3.99 |
| 1689 | Pentaethylene glycol octyl ether | 32.17 | 35.86 | 36.16 | -3.99 |
| 1690 | Diethyl O-octanoylmalate | 29.70 | 33.57 | 33.70 | -4.00 |
| 1691 | Ethyl vinyl ether | 19.00 | 21.80 | 23.01 | -4.01 |
| 1692 | Benzaldehyde | 38.00 | 34.38 | 33.98 | 4.02 |
| 1693 | Methylthiocyanate | 38.05 | 35.68 | 34.03 | 4.02 |
| 1694 | Methyl 2-methylacetoacetate | 36.03 | 31.93 | 32.01 | 4.02 |
| 1695 | 2-Methylpropanal | 20.31 | 24.08 | 24.34 | -4.03 |
| 1696 | Ethyl isothiocyanate | 36.04 | 33.31 | 32.00 | 4.04 |
| 1697 | Isobutyl chloride | 22.20 | 26.27 | 26.24 | -4.04 |
| 1698 | trans-2-Hexene | 18.08 | 22.30 | 22.14 | -4.06 |
| 1699 | Dibutyl phthalate | 33.40 | 37.46 | 37.47 | -4.07 |
| 1700 | Trimethylethylene | 17.64 | 21.75 | 21.71 | -4.07 |
| 1701 | 1-Ethyl-3-methylimidazolium ethosulfate | 42.50 | 38.76 | 38.38 | 4.12 |
| 1702 | Methyl 1-naphthaleneacetate | 45.14 | 41.55 | 41.01 | 4.13 |
| 1703 | Methyl trans-hexahydroindane-2,2-diacetate | 35.67 | 39.87 | 39.80 | -4.13 |
| 1704 | Cyclopentene | 22.95 | 27.04 | 27.10 | -4.15 |
| 1705 | 3-Methyl-4-(2,2,6-trimethylcyclohexyl)-2-butanol | 31.26 | 27.18 | 27.10 | 4.16 |
| 1706 | 2-Methyl-2-propanol | 20.41 | 24.50 | 24.58 | -4.17 |
| 1707 | Perfluoromethylcyclohexane | 15.70 | 19.59 | 19.88 | -4.18 |
| 1708 | Methylcyanoacetate | 39.17 | 34.83 | 34.98 | 4.19 |
| 1709 | 2-Methyl-1-penten-4-one | 23.00 | 26.90 | 27.19 | -4.19 |
| 1710 | 2-Propanol | 21.35 | 25.44 | 25.55 | -4.20 |
| 1711 | Vinyl acetate | 23.95 | 26.94 | 28.15 | -4.20 |
| 1712 | Isopentene | 16.51 | 20.80 | 20.72 | -4.21 |
| 1713 | 1-Butyl-3-methylimidazolium threoninate | 51.10 | 47.78 | 46.88 | 4.22 |
| 1714 | Glyceryltriacetate | 36.26 | 32.17 | 32.02 | 4.24 |
| 1715 | Bromoethane | 24.20 | 28.47 | 28.49 | -4.29 |
| 1716 | Diethyl O-decanoylmalate | 29.91 | 33.97 | 34.22 | -4.31 |
| 1717 | Heptyl 2-naphthaleneacetate | 35.69 | 40.05 | 40.01 | -4.32 |
| 1718 | Tris(5-H-perfluoropentyl) tricarballylate | 27.20 | 30.56 | 31.53 | -4.33 |
| 1719 | Heptyl 1-naphthoate | 35.87 | 39.85 | 40.20 | -4.33 |
| 1720 | 3,5-Xylenol | 28.04 | 32.08 | 32.39 | -4.35 |
| 1721 | 1-Butyl-3-methylimidazolium L-alanine | 46.10 | 42.12 | 41.73 | 4.37 |
| 1722 | Allyl chloride | 23.61 | 27.84 | 27.98 | -4.37 |
| 1723 | 2-Methylacrylonitrile | 24.40 | 28.68 | 28.78 | -4.38 |
| 1724 | 1-Pentyl-3-methylimidazolium acetate | 34.10 | 38.23 | 38.50 | -4.40 |
| 1725 | Butyl thiocyanate | 30.42 | 33.66 | 34.82 | -4.40 |
| 1726 | 3-Methoxyphenol | 42.20 | 38.99 | 37.79 | 4.41 |
| 1727 | Hexyl 1-naphthaleneacetate | 35.79 | 39.85 | 40.20 | -4.41 |
| 1728 | 1,2-Dipropoxycyclohexane | 33.50 | 28.94 | 29.08 | 4.42 |
| 1729 | Triisopropyl borate | 19.02 | 22.42 | 23.45 | -4.43 |
| 1730 | 1-Fluorobutane | 17.72 | 20.81 | 22.15 | -4.43 |
| 1731 | 1-Allylglycerol | 33.30 | 37.15 | 37.76 | -4.46 |
| 1732 | 1,6-Bis(7-H-perfluoroheptoxy)hexane | 25.30 | 21.68 | 20.82 | 4.48 |
| 1733 | Cetene | 27.75 | 23.55 | 23.27 | 4.48 |
| 1734 | 1,4-Diiodobutane | 47.08 | 42.80 | 42.59 | 4.49 |
| 1735 | 2-(2-Hydroxyethyl)-thiophene | 40.30 | 35.93 | 35.80 | 4.50 |
| 1736 | 1,2-Bis(4-tolyl)ethane | 33.60 | 37.58 | 38.10 | -4.50 |
| 1737 | Ethyl cyanoacetate | 36.62 | 32.33 | 32.12 | 4.50 |
| 1738 | Pyridine | 37.21 | 33.01 | 32.70 | 4.51 |
| 1739 | 1-Propyl-3-methylimidazolium tetracyanoborate | 46.00 | 42.31 | 41.49 | 4.51 |
| 1740 | 1-Octadecene | 28.45 | 23.99 | 23.93 | 4.52 |
| 1741 | 1-Ethyl-3-methylimidazolium methylsulfate | 45.10 | 41.26 | 40.57 | 4.53 |
| 1742 | 1-Heptadecene | 28.14 | 23.77 | 23.60 | 4.54 |
| 1743 | n-Butyl methyl ether | 20.05 | 24.50 | 24.59 | -4.54 |
| 1744 | Ethyl ether | 17.11 | 21.60 | 21.66 | -4.55 |
| 1745 | 2-Propyn-1-formate | 35.00 | 30.78 | 30.44 | 4.56 |
| 1746 | Campholanic acid | 32.21 | 27.64 | 27.64 | 4.57 |
| 1747 | N-Propylpyridinium tetrafluoroborate | 50.97 | 47.27 | 46.40 | 4.57 |
| 1748 | 1-(2-Aminoethyl)-3-methylimidazolium tetrafluoroborate | 53.50 | 49.71 | 48.91 | 4.59 |
| 1749 | 2-Pyridinecarboxaldehyde | 41.53 | 37.23 | 36.94 | 4.59 |
| 1750 | Formaldehyde diethylacetal | 20.29 | 23.76 | 24.89 | -4.60 |
| 1751 | cis-3-Hexene | 17.93 | 22.30 | 22.53 | -4.60 |
| 1752 | Triisoamylamine | 24.34 | 20.29 | 19.73 | 4.61 |
| 1753 | 1,4-Bis(propanoyloxy)-2-butyne | 36.16 | 32.00 | 31.49 | 4.67 |
| 1754 | Dimethyl ethylphosphonate | 36.63 | 32.46 | 31.91 | 4.72 |
| 1755 | 1-Hexanesulfonylchloride | 31.10 | 34.67 | 35.85 | -4.75 |
| 1756 | 2-Methoxyethyl dihydrogen phosphate | 36.61 | 32.68 | 31.85 | 4.76 |
| 1757 | cis-2-Pentene | 17.39 | 22.08 | 22.15 | -4.76 |
| 1758 | Glycerol tricaproate | 29.93 | 34.57 | 34.70 | -4.77 |
| 1759 | Heptyl 1-naphthaleneacetate | 35.18 | 40.05 | 39.97 | -4.79 |
| 1760 | 1-Hexyl-3-methylimidazolium tetrafluoroborate | 41.00 | 45.37 | 45.79 | -4.79 |
| 1761 | 1-Ethyl-3-propylimidazolium bis(trifluoromethanesulfonyl) amide | 33.41 | 28.94 | 28.61 | 4.80 |
| 1762 | 1-Ethyl-3-methylimidazolium trifluoromethylsulfonate | 39.20 | 35.58 | 34.38 | 4.82 |
| 1763 | Benzylamine | 39.07 | 34.50 | 34.24 | 4.83 |
| 1764 | Methyl picolinate | 43.48 | 38.88 | 38.65 | 4.83 |
| 1765 | Hexaethylene glycol octyl ether | 32.85 | 37.68 | 37.69 | -4.84 |
| 1766 | Ethylamine | 19.89 | 24.92 | 24.75 | -4.86 |
| 1767 | Thiane | 36.11 | 31.64 | 31.25 | 4.86 |
| 1768 | 1-Fluoronaphthalene | 38.21 | 34.31 | 33.34 | 4.87 |
| 1769 | trans-3-Hexene | 17.64 | 22.30 | 22.53 | -4.89 |
| 1770 | Ethanediol diacetate | 33.01 | 28.12 | 28.11 | 4.90 |
| 1771 | Pentaethylene glycol dodecyl ether | 32.00 | 36.66 | 36.91 | -4.91 |
| 1772 | Tetrahydrofurfuryl alcohol | 37.00 | 32.22 | 32.07 | 4.93 |
| 1773 | trans-2-Pentene | 17.14 | 22.08 | 22.15 | -5.01 |
| 1774 | Isopropylamine | 19.53 | 23.87 | 24.56 | -5.03 |
| 1775 | 1-Pentene | 16.00 | 21.13 | 21.08 | -5.08 |
| 1776 | 1-Hexyl-1,8-diazabicyclo(5,4,0)undec-7-enium thiocyanate | 37.98 | 34.07 | 32.89 | 5.09 |
| 1777 | Acetic anhydride | 31.93 | 29.09 | 26.83 | 5.10 |
| 1778 | 1-Hexyl-3-methylimidazolium trifluoromethylsulfonate | 32.58 | 36.38 | 37.68 | -5.10 |
| 1779 | Cyclopropanecarboxylic acid | 34.48 | 29.62 | 29.36 | 5.12 |
| 1780 | Trichlorotoluene | 40.84 | 36.24 | 35.72 | 5.12 |
| 1781 | 2-Propyne-1-ol | 36.05 | 31.08 | 30.92 | 5.13 |
| 1782 | Octyl octanoate | 31.43 | 26.35 | 26.27 | 5.16 |
| 1783 | 1,3-Dioxolane | 32.61 | 28.32 | 27.45 | 5.16 |
| 1784 | Aziridine | 32.80 | 27.62 | 27.62 | 5.18 |
| 1785 | 3-Ethyl-4-methylpyridine | 36.02 | 31.13 | 30.84 | 5.18 |
| 1786 | Dimethyl phosphite | 37.58 | 32.78 | 32.38 | 5.20 |
| 1787 | 1-Butyl-3-methylimidazolium dimethylphosphate | 38.00 | 42.30 | 43.23 | -5.23 |
| 1788 | 1-Propanesulfonylchloride | 38.00 | 34.07 | 32.76 | 5.24 |
| 1789 | n-Butylstearate | 32.70 | 27.55 | 27.44 | 5.26 |
| 1790 | 1-Hexyne | 20.98 | 26.02 | 26.26 | -5.28 |
| 1791 | Campholenic acid | 35.12 | 30.03 | 29.82 | 5.30 |
| 1792 | 1-Chloropropane | 21.92 | 27.19 | 27.24 | -5.32 |
| 1793 | 2-Hydroxybenzaldehyde | 42.90 | 38.14 | 37.54 | 5.36 |
| 1794 | Diethyl bromomethylmalonate | 30.77 | 36.09 | 36.13 | -5.36 |
| 1795 | Isopentane | 14.99 | 20.36 | 20.38 | -5.39 |
| 1796 | Diethyl tartrate | 37.97 | 42.70 | 43.37 | -5.40 |
| 1797 | Tributyl phosphate | 25.14 | 29.40 | 30.54 | -5.40 |
| 1798 | 1,2-Diethoxycyclohexane | 33.90 | 28.54 | 28.47 | 5.43 |
| 1799 | 2-(2-Chloroethoxy)ethyl-2-(2-hydroxyethoxy)ethyl ether | 43.62 | 38.23 | 38.17 | 5.45 |
| 1800 | Dibutyl perfluoroadipate | 22.20 | 27.32 | 27.65 | -5.45 |
| 1801 | n-Butylnitrite | 31.21 | 25.74 |  | 5.47 |
| 1802 | Pentane | 16.05 | 21.48 | 21.55 | -5.50 |
| 1803 | Hexyl 1-naphthylketone | 36.47 | 41.88 | 41.99 | -5.52 |
| 1804 | 1-Hexyl-3-methylimidazolium acetate | 33.00 | 38.43 | 38.54 | -5.54 |
| 1805 | Heptyl 1-naphthylketone | 36.77 | 42.08 | 42.32 | -5.55 |
| 1806 | 1,2-Dimethylnaphthalene | 39.20 | 33.88 | 33.65 | 5.55 |
| 1807 | 2-Chloropropane | 19.69 | 24.46 | 25.25 | -5.56 |
| 1808 | 1-Ethyl-3-methylimidazolium aminoacetate | 48.10 | 42.77 | 42.54 | 5.56 |
| 1809 | Perfluoro-2-methyldecalin | 19.20 | 23.25 | 24.76 | -5.56 |
| 1810 | 1-Ethyl-3-(6-hydroxyhexyl)-imidazolium chloride | 42.30 | 46.48 | 47.91 | -5.61 |
| 1811 | 1,4-Dioxane | 33.45 | 27.98 | 27.78 | 5.67 |
| 1812 | Methanol | 22.50 | 28.06 | 28.20 | -5.70 |
| 1813 | 1-Ethyl-3-methylimidazolium hexylsulfate | 34.77 | 39.56 | 40.50 | -5.73 |
| 1814 | DEHP | 31.20 | 36.82 | 36.95 | -5.75 |
| 1815 | 1-Butyl-3-methylimidazolium chloride | 48.20 | 43.30 | 42.43 | 5.77 |
| 1816 | Benzylbromide | 32.33 | 38.05 | 38.12 | -5.79 |
| 1817 | 1-Acetonaphthone | 46.16 | 40.88 | 40.37 | 5.79 |
| 1818 | 1-Butyl-1-methylpyrrolidinium tris(pentafluoroethyl)trifluorophosphate | 38.00 | 32.97 | 32.20 | 5.80 |
| 1819 | Azelaonitrile | 42.83 | 37.54 | 37.03 | 5.80 |
| 1820 | 1,3-Diiodopropane | 47.87 | 42.60 | 42.07 | 5.80 |
| 1821 | 1,2-Diisopropoxycyclohexane | 33.40 | 28.30 | 27.58 | 5.82 |
| 1822 | Isobutyl isobutyrate | 28.38 | 22.51 | 22.53 | 5.85 |
| 1823 | 2,2,4,4,6,8,8-Heptamethylnonane | 24.32 | 18.80 | 18.45 | 5.87 |
| 1824 | 1,3-Propanediol dinitrate | 45.60 | 40.22 | 39.72 | 5.88 |
| 1825 | 3-Methyl-1-butene | 14.36 | 20.01 | 20.24 | -5.88 |
| 1826 | 1-Octyl-3-methylimidazolium tetracyanoborate | 38.60 | 43.31 | 44.49 | -5.89 |
| 1827 | 1-Ethyl-3-methylimidazolium L-proline | 39.60 | 44.82 | 45.55 | -5.95 |
| 1828 | 1-Naphthaleneacetonitrile | 48.95 | 43.06 | 42.98 | 5.97 |
| 1829 | 2-Ethoxyethylacetate | 31.80 | 25.77 | 25.71 | 6.09 |
| 1830 | 1-Ethyl-3-methylimidazolium lactate | 48.80 | 43.29 | 42.70 | 6.10 |
| 1831 | 2,4-Dichloronitrobenzene | 42.77 | 47.00 | 48.93 | -6.16 |
| 1832 | 4-Acetylpyridine | 44.01 | 38.17 | 37.81 | 6.20 |
| 1833 | Dimethylacetal | 21.58 | 27.40 | 27.84 | -6.26 |
